# Electron‐Deficient Imidazolium Substituted Cp Ligands and their Ru Complexes

**DOI:** 10.1002/chem.202002801

**Published:** 2020-09-30

**Authors:** Fabio Mazzotta, Georg Zitzer, Bernd Speiser, Doris Kunz

**Affiliations:** ^1^ Institut für Anorganische Chemie Eberhard Karls Universität Tübingen Auf der Morgenstelle 18 72076 Tübingen Germany; ^2^ Institut für Organische Chemie Eberhard Karls Universität Tübingen Auf der Morgenstelle 18 72076 Tübingen Germany

**Keywords:** cyclopentadienides, electron-poor ligands, imidazoliums, ruthenocenes, ylides

## Abstract

The synthesis of electron‐poor mono‐, di‐ and tri(imidazolium)‐substituted Cp‐ylides is presented and their electronic properties are discussed based on NMR spectroscopy, X‐ray structure analyses, electrochemical investigations and DFT calculations as well as by their reactivity toward [Ru(CH_3_CN)_3_Cp*](PF_6_). With mono‐ and di(imidazolium)‐substituted cyclopentadienides the respective monocationic and dicationic ruthenocences are formed (X‐ray), whereas tri(imidazolium) cyclopentadienides are too electron‐poor to form the ruthenocenes. Cyclic voltammetric analysis of the ruthenocenes shows reversible oxidation at a potential that increases with every additional electron‐withdrawing imidazolium substituent at the Cp ligand by 0.53–0.55 V in an electrolyte based on a weakly coordinating anion. A reversible oxidation can be observed for the free 1,3‐disubstituted ligand as well.

## Graphical abstract

**Ligand design and application**: The synthesis of electron‐poor imidazolium substituted Cp‐ylides is presented. Their electronic properties are elucidated based on NMR spectroscopy, DFT and reactivity studies. The first η^5^‐Cp coordination of electron‐poor cationic ylides is confirmed by X‐ray structure analyses and an equilibrium for the coordination of a Cp ligand is observed. Electrochemical investigations of the ligands and of the ruthenocenes quantify the decreasing donor properties with increasing number of imidazolium substituents.

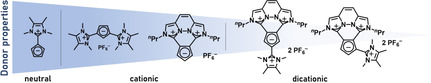

## Introduction

Cp ligands are among the most common ligands in organometallic chemistry and tuning of the steric and electronic properties by introducing different substituents is well known.[^1^, ^2^, ^3^] Each substituent at the Cp ring will increase its steric demand and in most cases also the electron density in the Cp ring so that the M−Cp bond strength increases. Substituents that weaken the M−Cp bond (e.g. Ar,^[4]^ F,^[5]^ Cl,^[5]^ CN, COOR, NO_2_, CF_3_,^[6]^ phosphonium,^[7]^ pyridinium or ammonium groups) are much less studied.[^8^, ^9^]

**Figure 1:**
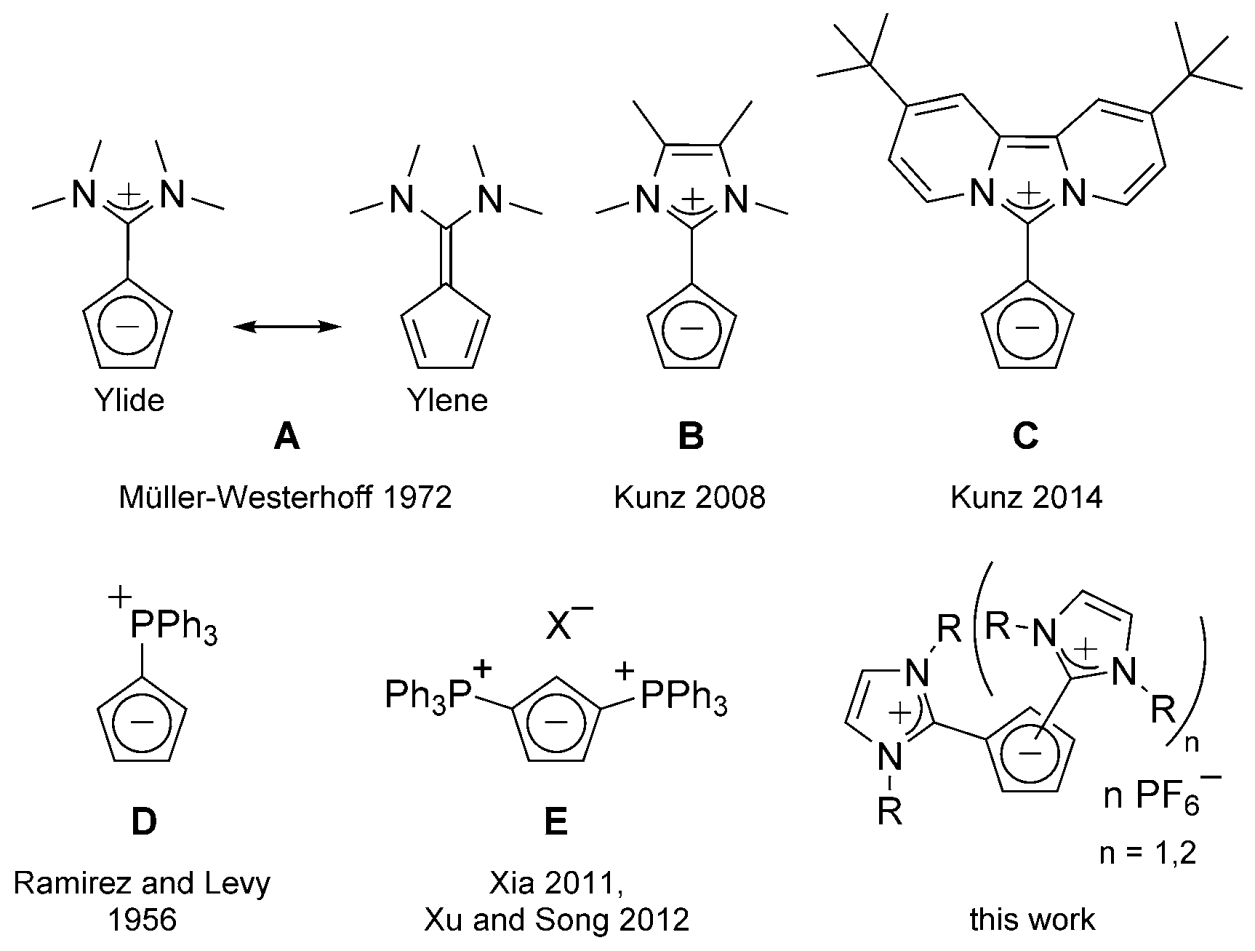
Selection of literature known cyclopentadienylides **A**‐**E** and the new di‐ and tri(imidazolium) Cps of this work.

One interesting ligand class in this regard is that of cyclopentadienylides. These zwitterionic compounds are thermodynamically stable Cp compounds containing at least one α‐cationic substituent at the Cp ring, e.g. ‐SR_2_^+^, ‐NR_3_^+^, ‐PR_3_^+^ or carbenium.[^7^, ^9^] The latter can also be described with the resonance structure of ylenes, for example, as fulvenes or fulvalenes (Figure 1). In case of an enhanced stabilisation of the separated charges by mesomeric effects or aromaticity, the ylidic character can become dominant. In early works, Müller‐Westerhoff introduced the diamino fulvene **A** as a cationic Cp ligand in ferrocene by simple addition to iron(II) chloride at 60 °C.^[10]^ This indicates a sufficient polarity of the exocyclic double bond and a Cp like reactivity of these compounds. With the idea of further enhancing the ylidic character by aromatic stabilization, we reported in 2008 on the first imidazolium cyclopentadienylide **B**, which can also be regarded as a diazafulvalene, and the formation of its ferrocene.^[11]^ This compound exhibits a highly ylidic character and can be best described as an imidazolium substituted Cp without significant ylene character. Six years later, we introduced the dipyrido‐anellated ligand **C**, which provides an even stronger charge separation.^[12]^ An analogous class of compounds are phosphonium cyclopentadienylides,^[7]^ with the Ramirez ylide **D** being its most prominent representative.^[13]^ The first examples of di(phosphonium) substituted Cp rings **E** have been reported recently.[^14^, ^15^] While the coordination chemistry of the monosubstituted Cp ligands **A**–**D** has been already investigated, that of the cationic disubstituted Cp ligand **E** remains unexplored so far.

The imidazolium cyclopentadienylides **B** and **C** were used as ligands for half‐sandwich and sandwich complexes[^11^, ^16^, ^17^] by simple addition to a free coordination site of a suitable metal precursor or by ligand substitution. As the cyclopentadienylides contain a positive charge at the α‐position of the substituents, their electron density is reduced compared to regular Cps. At this point, no detailed investigation of the donor properties of cyclopentadienylide **B** had been made. Therefore, we will present this together with an improved synthesis of **B** as well as the synthesis and properties of the first di(imidazolium) cyclopentadienylides and tri(imidazolium) cyclopentadienylides. To elucidate the electronic properties of these Cp ligands, we carried out DFT calculations and, if possible, prepared the respective ruthenocenes by reaction with [Ru(CH_3_CN)_3_Cp*](PF_6_) and analyzed the oxidation potential of the ligands as well as of the ruthenocenes.

## Results and Discussion

### Improved synthesis for imidazolium cyclopentadienylide 2

**Scheme 1:**
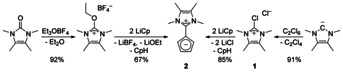
Previous (left) and improved (right) synthesis of the imidazolium cyclopentadienylide **2**.

Until now, the synthesis of the monosubstituted compounds **B** or **C** required the synthesis of the corresponding uronium or guanidinium salts[^11^, ^12^] that were reacted with two equivalents of either LiCp or NaCp (Scheme 1). One equivalent was needed as nucleophile to obtain the protonated Cp intermediates and the second to deprotonate them under generation of the desired imidazolium cyclopentadienylides. However, in the case of the uronium salts side reactions at the alkoxy group can lead to the corresponding imidazolone.^[18]^ To avoid also the formation of LiBF_4_ as a tediously to remove byproduct, we changed the leaving group from ethoxide to chloride. Chloroimidazolium salts are in general obtained from chlorination of the free carbene if the backbone is alkylated.^[19]^ In our case Kuhn's 2‐chloro‐tetramethylimidazolium chloride (**1**)[^20^, ^21^, ^22^] proved to be a suitable precursor for introducing the imidazolium substituent to Cps.

However, we found it advantageous to change the reaction conditions of the synthesis of **1** to firstly cooling the solution of a slight excess of hexachloroethane in diethyl ether to −30 °C and then adding dropwise a solution of the free carbene under vigorous stirring. We were also able to elucidate the molecular structure of **1** by X‐ray crystal structure analysis, which exhibits intermolecular halogen‐halogen interactions (see Supporting Information). Kuhn mentioned the high reactivity and decomposition of **1** in polar solvents,^[21]^ which we found not to be the case in acetonitrile.

**Figure 2:**
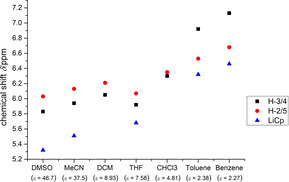
Solvent‐dependent chemical shift of the Cp−H signals (black and red) of **2** in comparison with those of LiCp (blue).

With the chloride **1** in hand, we synthesized cyclopentadienylide **2** achieving an improved yield and purity. As our previous work showed a fast H/D exchange of the Cp protons of **2** with D_2_O but no decomposition,^[11]^ we could remove residual lithium salts with degassed water. The ^1^H NMR chemical shifts of the Cp signals of **2** are strongly depending on the polarity of the solvent (Figure 2). Except for tetrahydrofuran, the signals are shifted downfield with lower polarity of the solvent. Interestingly, the β‐signals (3/4‐H) are more strongly affected than the α‐signals (2/5‐H). The signal of LiCp shows qualitatively the same trend.

**Scheme 2:**
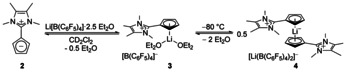
Coordination of ylide **2** to Li[B(C_6_F_5_)_4_]⋅2.5Et_2_O.

In a previous work, we reported on the facile coordination of cyclopentadienylide **C** to LiNTf_2_ under formation of Li(η^5^‐Cp) complexes that are in a fast equilibrium between a half‐sandwich and a lithiocene complex in toluene already at −80 °C.^[17]^ Therefore, we tested ylide **2** in an analogous NMR experiment, and used Li[B(C_6_F_5_)_4_]⋅2.5Et_2_O as well as LiNTf_2_ as lithium salt and deuterated dichloromethane as a polar, weakly coordinating solvent (Scheme 2). In the case of LiNTf_2_ we observe one broad signal in the ^7^Li NMR at −7.0 ppm (LiNTf_2_) that is diagnostic for a half‐sandwich lithium complex. In the case of Li[B(C_6_F_5_)_4_]⋅2.5Et_2_O we observe at room temperature two broad signals. The first at 0.8 ppm can be assigned to a lithium cation and the second at −7.3 ppm (Li[B(C_6_F_5_)_4_]) again to the half‐sandwich complex. At −80 °C (Li[B(C_6_F_5_)_4_]) we observe three lithium signals that can be assigned to a Li^+^ cation at −0.80 ppm, and in a 1:5 ratio to the half‐sandwich lithium complex **3** at −6.0 ppm and to the lithiocene **4** at −11.7 ppm. The broad signals indicate a fast equilibrium between these species that is confirmed by a ^7^Li EXSY NMR experiment. To compare it directly with **C**, we performed the experiment with LiNTf_2_ in toluene, but even at −80 °C only the half‐sandwich complex can be observed.

### Synthesis of 1,3‐di(imidazolium)cyclopentadienylide (5)

**Scheme 3:**
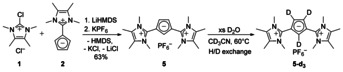
Synthesis of the di(imidazolium) cyclopentadienylide **5** and H/D exchange of the Cp protons.

To synthesize the imidazolium analog of the di(phosphonium) cyclopentadienylide **E**, we used the monosubstituted ylide **2** as a nucleophile to react with the chloroimidazolium salt **1**. The reaction is analogous to the synthesis of the ylide **2**, but with the difference that LiHMDS is used as a bulky and weakly nucleophilic auxiliary base (Scheme 3). The reaction is much slower, due to the weaker nucleophilicity of **2** compared to LiCp. The reduced nucleophilicity caused by each additional imidazolium substituent is the reason why a second substitution during the synthesis of **2** or a third substitution during the synthesis of **5** are not observed. For the same reason, the work up of **5** can be performed under air. Notably, only the 1,3‐substituted product is formed. A 1,2‐substitution pattern might be impeded by steric effects and Coulomb repulsion. Changing the solvent from acetonitrile to tetrahydrofuran resulted in longer reaction times, possibly due to the poor solubility of both starting materials, but in the same purity of **5** (NMR).

Attempts to deprotonate compound **5** to form an NHC analogue failed so far due to the very low acidity of the C−H protons. Moreover, the coordination to lithium salts under the same conditions as with **2** did not take place. However, we observed a slow H/D exchange of the Cp protons in D_2_O already at room temperature. To accelerate the exchange, we heated the solution to 60 °C. Most probably the exchange proceeds by a protonation–deprotonation mechanism. We also tried to freeze the rotation of the imidazolium substituents at −80 °C and quantify the rotation barrier, but even at −80 °C no second signal set is observed. This observation matches with a high single bond character in the exocyclic C−C bonds.

**Figure 3:**
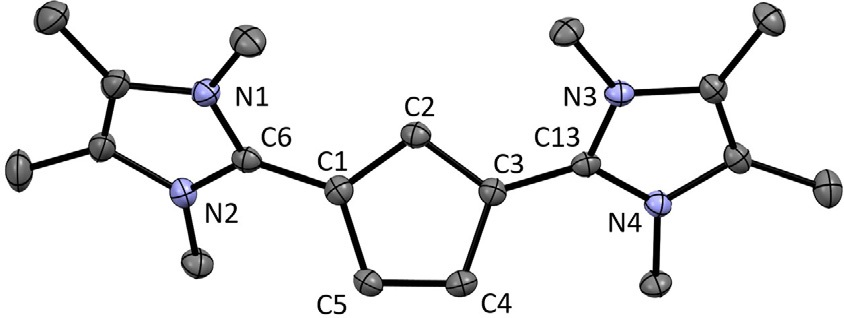
ORTEP plot of the molecular structure of **5** (anisotropic atomic displacement parameters at 50 % probability). Hydrogen atoms and the PF_6_^−^ counterion are omitted for clarity. Selected bond lengths [Å] and angles [°]: C1−C6 1.440(3), C3−C13 1.439(2), C1−C2 1.406(2), C2−C3 1.411(2), C3−C4 1.431(2), C4−C5 1.378(3), C5−C1 1.427(2); N1‐C6‐N2 106.2(2), N3‐C13‐N4 106.1(1), C5‐C1‐C2 108.1(2), C1‐C2‐C3 107.7(2), C2‐C3‐C4 107.6(1), C3‐C4‐C5 108.4(2), C4‐C5‐C1 108.2(2), N1‐C6‐C1‐C2 43.4, N3‐C13‐C3‐C2 35.6.

The molecular structure could be obtained from single crystals by X‐ray structure analysis (Figure 3). The bond lengths of the Imi−Cp bonds (C1−C6 and C3−C13, 1.440(3) and 1.439(3) Å) are longer than for **2** (C1−C3 1.430(3) Å).^[11]^ This fact can either be attributed to a higher single bond character compared to **2** per se and/or to a lower Coulomb attraction due to the additional cationic moiety. The bond lengths of the Cp ring lie between 1.431(2) and 1.378(3) Å, and are comparable with those of **1**. The angles of the Cp ring are very close to 108°, the value of a regular pentagon.

### Characterization of side product 6

**Figure 4:**
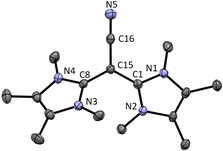
ORTEP plot of the molecular structure of **6** (anisotropic atomic displacement parameters at 50 % probability). Hydrogen atoms and the PF_6_^−^ counterion are omitted for clarity. Selected bond lengths [Å] and angles [°]: N5−C16 1.153(2), C15−C16 1.410(2), C1−C15 1.432(2), C8−C15 1.432(2); N1‐C1‐N3 106.9(1), N3‐C8‐N4 107.0(1), C1‐C15‐C8 123.0(1).

During the synthesis of **5**, we could observe from the crude product a peak in the ESI–MS^+^ at *m*/*z=*286.1, which fits to a combination of two imidazolium and one (deprotonated) acetonitrile moiety. Although, we were not able to optimize the synthesis and isolate this interesting air‐stable compound with the desired purity, we obtained single crystals of **6** suitable for X‐ray structure analysis, which confirms the anticipated structure (see Figure 4). The bond lengths N5−C16 1.153(2) Å and C15−C16 1.410(2) Å are comparable with the lengths of acetonitrile (C−N 1.150(4) Å and C−C 1.442(4) Å)^[23]^ and the calculated structure (C−N 1.176 Å and C−C 1.405 Å). The most plausible resonance structure is that of a C≡N triple bond and the negative charge mainly localized on the adjacent carbon atom. Analogous di(phosphonium)ylides are known from Erker and co‐workers.^[24]^

**Scheme 4:**
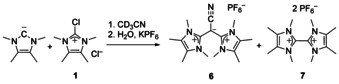
Independent synthesis of **6**, which also leads to formation of **7**.

We could reproduce this peak and therefore the formation of **6** by mixing the free carbene with the chloroimidazolium salt **1** in acetonitrile (Scheme 4). After short workup, we obtained a mixture of two species in acetonitrile. The ESI–MS^+^ revealed again the peak at *m*/*z=*286.1 for the product **6** and two peaks at *m*/*z=*124.1 for a dicationic species and *m*/*z=*393.1 for a mono cationic species. Furthermore, we could remove the second so far unknown species **7** by extraction of the raw product **6** with dichloromethane. As it is known, di(imidazolium) salts can be formed under these conditions.[^25^, ^26^] We confirmed formation of **7** by independent synthesis and characterization (Supporting Information).

### Synthesis of 1,2‐di(imidazolium)cyclopentadienylide 9

**Scheme 5:**
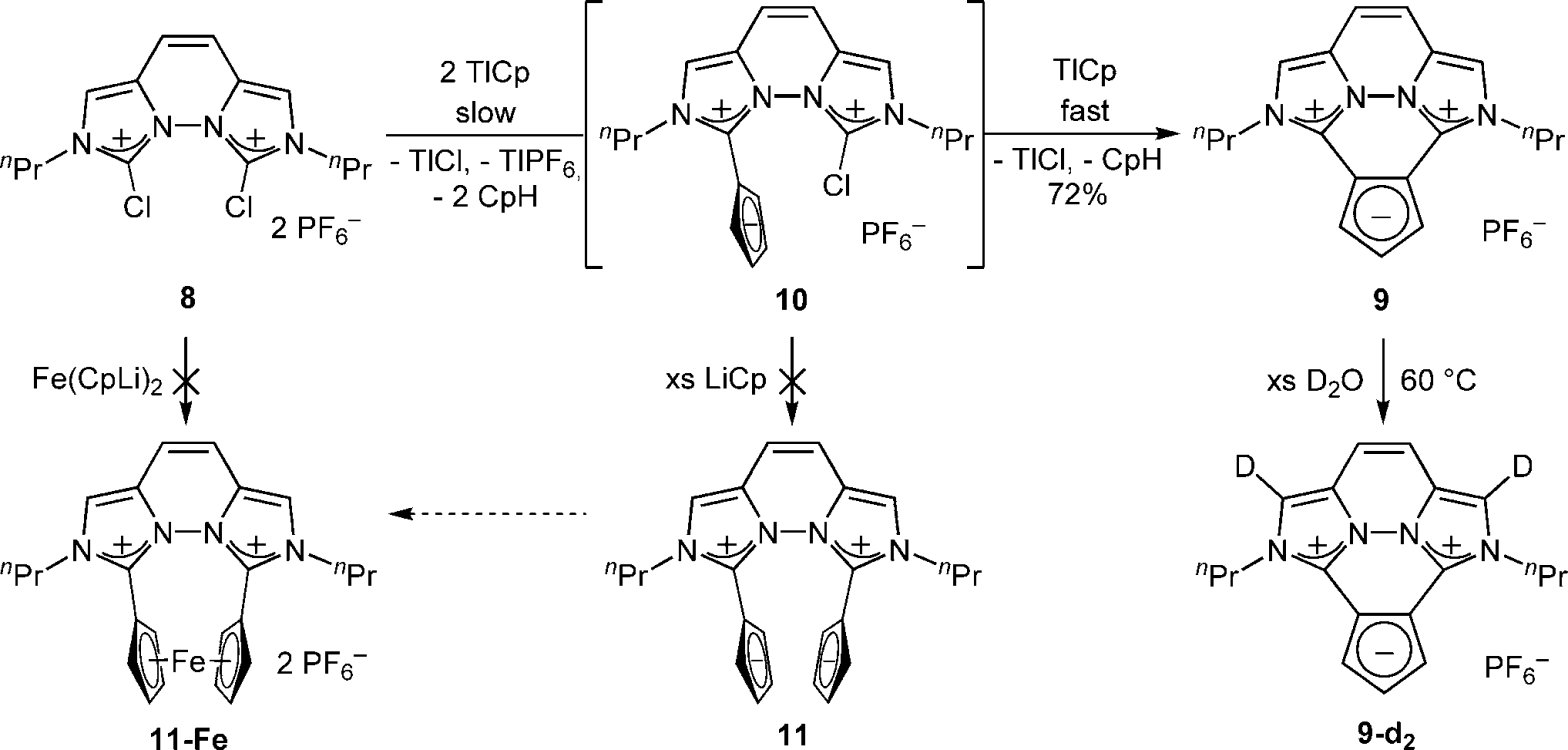
Synthesis of the 1,2‐substituted Cp‐ylide **9** and H/D exchange of the Cp protons. The potential di(Cp‐ylides) **11** and **11‐Fe** were not obtained.

To obtain also a 1,2‐substitution pattern of a di(imidazolium) Cp, we reacted the di(chloroimidazolium) salt **8**[^12^, ^27^] with two equivalents of LiCp or NaCp (see Scheme 5). In both cases, full conversion was obtained within one hour and the desired product was detected, but some brown impurities impeded its isolation. Therefore, we used the less nucleophilic thallium(I) cyclopentadienide and after two days full conversion at no apparent side reactions was achieved (NMR). Just like with compound **5**, the coordination of the cationic ylide **9** to Li^+^ is not observed under the same conditions as applied for **2**. We observed a H/D exchange with D_2_O however, not of the Cp but of the imidazo hydrogen atoms (see Scheme 5). As these positions are known to be acidic from the synthesis of abnormal NHCs, they seem to be the most acidic hydrogen atoms of **9**.^[28]^

Interestingly, no monoylide **10** was observed, even when only one equivalent of LiCp was used (see Scheme 5). This shows that the intramolecular S_N_ reaction with the second chloroimidazolium moiety proceeds much faster than the intermolecular S_N_ reaction to form **10**, or an analogue of the 1,3‐disubstituted Cp compound **5**. Moreover, no diylide **11** can be observed with a 40‐fold excess of LiCp, even if the reaction is performed in tetrahydrofuran, in which **8** is poorly soluble compared the LiCp. The reaction of dilithioferrocene with the di(chloroimidazolium) salt **8** to form directly the corresponding dicationic *ansa*‐ferrocene led to no defined product.

**Figure 5:**
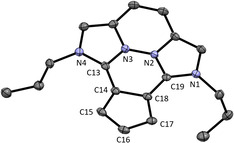
ORTEP plot of the molecular structure of **9** (anisotropic atomic displacement parameters at 50 % probability). Hydrogen atoms, one molecule of dichloromethane, and the PF_6_^−^ counterion are omitted for clarity. Selected bond lengths [Å] and angles [°]: C13−C14 1.414(3), C18−C19 1.418(3), C14−C15 1.402(3), C15−C16 1.399(3), C16−C17 1.403(3), C17−C18 1.400(3), C14−C18 1.457(3); N1‐C19‐N2 103.5(2), N3‐C13‐N4 103.7(2), C18‐C14‐C15 107.6(2), C14‐C15‐C16 107.5(2), C15‐C16‐C17 110.1(2), C16‐C17‐C18 107.7(2), C17‐C18‐C14 107.3(2), N1‐C19‐C18‐C17 3.0, N4‐C13‐C14‐C15 0.8.

The molecular structure of **9** could be elucidated with single crystal X‐ray structure analysis. As expected, **9** is planar (except for the *n*‐propyl groups) due to the conjugated π‐system (see Figure 5). The bond lengths of the Imi−Cp bonds (C13−C14 1.414(3) Å and C18−C19 1.418(3) Å) are shorter compared to compound **5**. Tentatively, this is caused by electronic effects of the conjugated, fixed planar π‐system formed. In contrast, the annellated Cp bond C14−C18 1.457(3) Å is much longer than any Cp bond in compound **5**. All other four Cp bonds have equal bond lengths of 1.40 Å within the standard deviation. The angles of the Cp ring are close to 108° as in **5**.

### Synthesis of tri(imidazolium) cyclopentadienylides 12 and 13

**Scheme 6:**
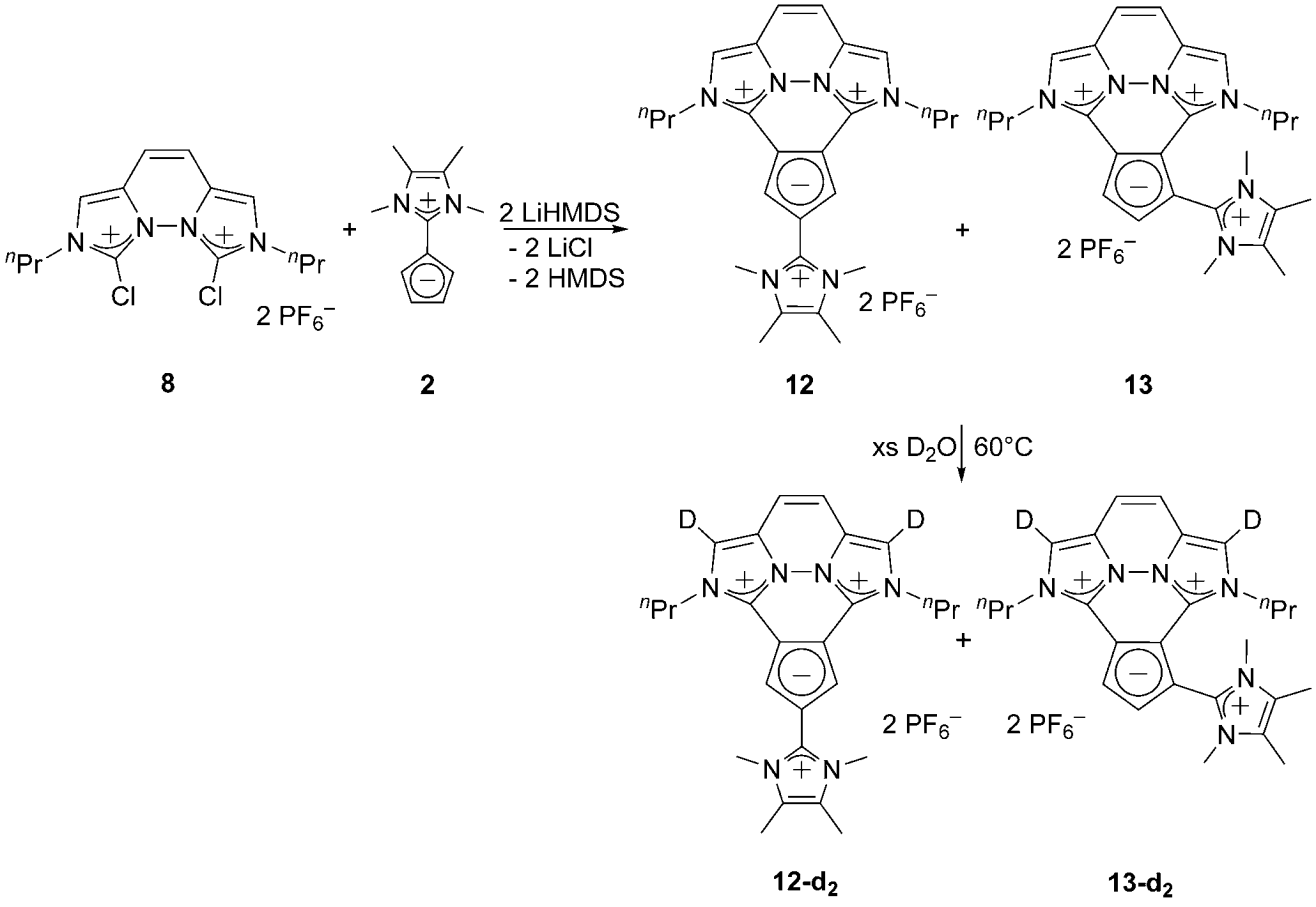
Synthesis of the tri(imidazolium) Cp‐ylides **12** and **13** and H/D exchange at the imidazo moieties.

As the nucleophilicity of the disubstituted compounds is reduced, an additional nucleophilic substitution by the chloroimidazolium salt **1** was not observed during the synthesis of **5**. However, as the intramolecular reaction is much faster, we took advantage of it and reacted the di(chloroimidazolium) compound **8** with **2** which is a weaker nucleophile than LiCp. In addition, we used LiHMDS as a sterically hindered base to form the tri‐substituted Cp‐ylides **12** and **13** (see Scheme 6). Monitoring the reaction by NMR spectroscopy showed full conversion of the starting material along with some side product. The ratio of 4.5:1 for **12**:**13** is equal for the reaction at room temperature and at −30 °C. Separation of these two salts of similar polarity failed until now.

The trisubstituted compounds were also tested in a H/D‐exchange experiment. As it was already revealed for **9**, the hydrogen atoms at the imidazo moieties of **12** and **13** were exchanged.

**Figure 6:**
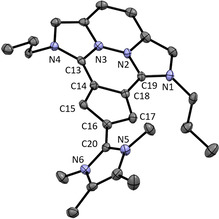
ORTEP plot of the molecular structure of **12** (anisotropic atomic displacement parameters at 50 % probability). Hydrogen atoms, one acetonitrile molecule and two PF_6_^−^ counterions are omitted for clarity. Selected bond lengths [Å] and angles [°]: C13−C14 1.413(5), C18−C19 1.422(5), C16−C20 1.452(5), C14−C15 1.390(5), C15−C16 1.403(5), C16−C17 1.414(5), C17−C18 1.385(5), C14−C18 1.456(4); N1‐C19‐N2 104.0(3), N3‐C13‐N4 103.6(3), N5‐C20‐N6 106.7(3), C18‐C14‐C15 107.5(3), C14‐C15‐C16 107.6(3), C15‐C16‐C17 109.7(3), C16‐C17‐C18 107.1(3), C17‐C18‐C14 108.1(3), N1‐C19‐C18‐C17 2.0, N4‐C13‐C14‐C15 2.5, N5‐C20‐C16‐C17 52.9.

**Figure 7:**
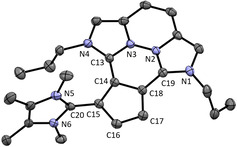
ORTEP plot of the molecular structure of **13** (anisotropic atomic displacement parameters at 50 % probability). Hydrogen atoms and two PF_6_^−^ counterions are omitted for clarity. Selected bond lengths [Å] and angles [°]: C13−C14 1.424(2), C18−C19 1.414(2), C15−C20 1.457(2), C14−C15 1.414(2), C15−C16 1.411(2), C16−C17 1.390(2), C17−C18 1.400(2), C14−C18 1.453(2); N1‐C19‐N2 104.2(1), N3‐C13‐N4 103.1(1), N5‐C20‐N6 107.1(1), C18‐C14‐C15 106.0(1), C14‐C15‐C16 107.8(1), C15‐C16‐C17 110.1(1), C16‐C17‐C18 107.2(1), C17‐C18‐C14 108.9(1), N1‐C19‐C18‐C17 9.3, N4‐C13‐C14‐C15 7.8, N5‐C20‐C16‐C17 76.6.

We were able to obtain the molecular structures of **12** and **13** by X‐ray structure analysis (see Figure 6 and 7). In both cases a planar anellated ring system with a third, almost perpendicular imidazolium substituent is revealed. The Imi−Cp bond lengths C13−C14 and C18−C19 of **12** are almost identical to those of compound **9**. Only the Imi−Cp bond C16−C20 from the additional substituent is longer compared to the respective bonds of **2** or **5**. In compound **13** the mean value of the Imi−Cp bonds C13−C14 and C18−C19 is comparable to that of **9** as well. The bond C15−C20 to the third imidazolium substituent is longer compared to **2** or **5**, which can be explained with the reduced Coulomb attraction of the Cp‐ring due to the additional imidazolium substituent. In both molecules **12** and **13** the angles in the Cp ring are still close to 108°.

**Figure 8:**
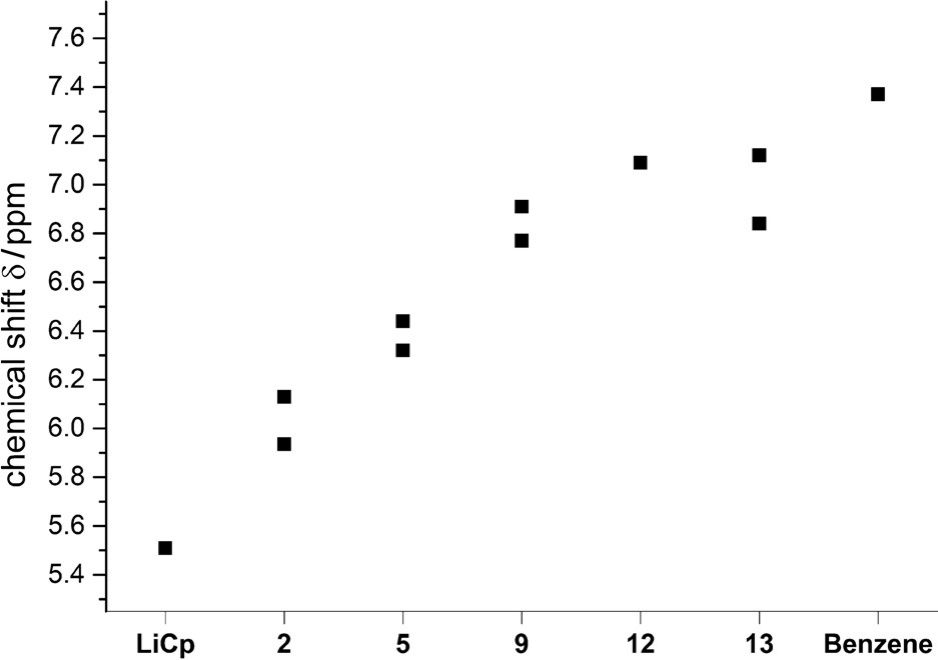
^1^H NMR chemical shifts of Cp−H signals of LiCp, the ylides **2**, **5**, **9**, **12**, **13** and benzene in deuterated acetonitrile.

When comparing the ^1^H NMR chemical shifts of the Cp signals of LiCp and the Cp−ylides **2**, **5**, **9**, **12** and **13** as well as the signal of benzene in deuterated acetonitrile, we noticed a downfield shift with every additional imidazolium substituent but also a downfield shift from the 1,3‐disubstituted system **5** to the planar 1,2‐disubstituted system **9** (see Figure 8).

### DFT calculations

**Figure 9:**
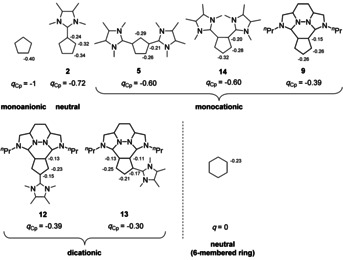
Selected natural charges and the sum of the natural charges of the Cp‐moiety *q*_Cp_ from the natural bond orbital (NBO) population analysis of the Cp anion, the ylides **2**, **5**, **14**, **9**, **12**, and **13** as well as of the benzene ring. Only the σ bonds between the C${}_{\mathrm{sp}{}^{2}}$ atoms are shown for clarity.

The Cp‐like reactivity of these compounds should be highly depending on the electron density in the Cp ring. Therefore, we carried out an NBO population analysis of the geometry optimized ligands (DFT, BP86/def2‐TZVP, COSMO: *ϵ*=37.5), which describes very well delocalized systems. For a reasonable comparison of the charges at the Cp ring, we summed up the natural charges of the atoms from the Cp fragment (*q*_Cp_), which can be correlated with the electron density. Compared to the non‐substituted Cp, the negative charge at the carbon atoms is reduced with every additional imidazolium substituent, except for the third substituent in **12** compared to **9** (see Figure 9). Besides the electron density, it should be considered that the Coulomb interactions also contribute significantly to the stability of the metal‐Cp bond.

In addition, we carried out the NBO population analysis of the hypothetical 1,2‐disubstituted non‐annellated compound **14** to compare the influence of the substitution pattern. A 1,2‐substitution leads to a different dipole moment of the molecule, but interestingly to the same charge of the Cp fragment. Therefore, we can assign the observed lower electron density of the Cp ring in the annellated system **9** to the fixed planar geometry of the imidazolium substituents and not to the substitution pattern.

The NBO population analysis of **2** reveals a slightly more negatively charged 3‐position of the Cp ring which explains the preferred 1,3‐substitution pattern in the nucleophilic substitution reaction.

**Figure 10:**
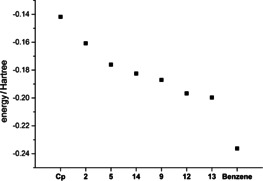
Calculated HOMO energies of the Cp anion, the ylides **2**, **5**, **14**, **9**, **12**, **13** and benzene.

With every additional imidazolium substituent, the energy of the highest‐occupied molecular orbital (HOMO) is decreasing (see Figure 10) as it is to be expected with electron withdrawing substituents. For all Cp ylides the HOMO is located at the Cp ring and has π‐character, but also with some contribution of the imidazolium substituents (Supporting Information). In comparison, the HOMO of benzene lies at considerably lower energies. The 1,2‐substitution of the virtual compound **14**, leads to a HOMO with pronouncedly lower energy compared to **5**. The LUMOs of the compounds are in some cases not Cp‐like and are therefore not discussed.

### Synthesis of the ruthenocenes

**Scheme 7:**
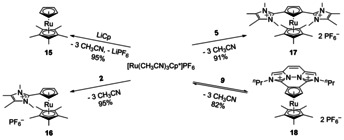
Synthesis of the ruthenocenes starting from [Ru(CH_3_CN)_3_Cp*](PF_6_).

To quantify the influence of our new ligands on the metal center, we decided to synthesize their respective mixed Cp*Ru‐complexes and compare their redox potential with the mixed ruthenocene [RuCp*Cp] (**15**). The synthesis of the literature known complex starts from [Ru(CH_3_CN)_3_Cp](PF_6_) using LiCp*^[29]^ and involves a low reaction temperature (−78 °C) and 14 h reaction time in total. However, we obtained a higher yield and comparable purity, when we started from [Ru(CH_3_CN)_3_Cp*](PF_6_) and LiCp (see Scheme 7) in acetonitrile as solvent and ran the reaction at room temperature for 10 min. The work up was kept identical to the literature procedure.

For the synthesis of the corresponding imidazolium substituted ruthenocenes, we also used [RuCp*(CH_3_CN)_3_](PF_6_) (a salt metathesis route is not possible) and reacted it with the ligands **2** and **5** in acetonitrile at room temperature. Both complexes **16** and **17** are air‐stable already during the workup of the reaction mixture.

**Figure 11:**
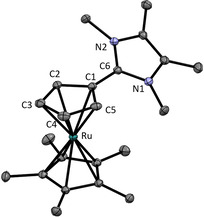
ORTEP plot of the molecular structure of **16** (anisotropic atomic displacement parameters at 50 % probability). Hydrogen atoms and the PF_6_^−^ anion are omitted for clarity. Selected bond lengths [Å] and angles [°]: C1−C6 1.454(2), C1−C2 1.443(2), C2−C3 1.426(2), C3−C4 1.424(2), C4−C5 1.423(2), C5−C1 1.443(2), Ru1−C1 2.195(1), Ru1−C2 2.181(1), Ru1−C3 2.193(1), Ru1−C4 2.212(1), Ru1−C5 2.207(1); N1‐C6‐N2 106.9(1), N1‐C6‐C1‐C2 36.5.

**Figure 12:**
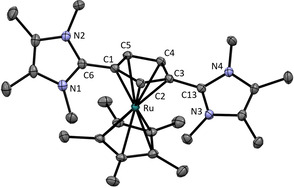
ORTEP plot of the molecular structure of **17** (anisotropic atomic displacement parameters at 50 % probability). Hydrogen atoms, one acetonitrile molecule and two PF_6_^−^ anions are omitted for clarity. Selected bond lengths [Å] and angles [°]: C1−C6 1.457(3), C3−C13 1.453(2), C1−C2 1.429(2), C2−C3 1.437(2), C3−C4 1.442(2), C4−C5 1.415(3), C5−C1 1.437(2), Ru1−C1 2.214(2), Ru1−C2 2.219(2), Ru1−C3 2.203(2), Ru1−C4 2.173(2), Ru1−C5 2.177(2); N1‐C6‐N2 107.1(2), N3‐C13‐N4 106.9(2), N1‐C6‐C1‐C2 49.1, N3‐C13‐C3‐C2 36.6.

The η^5^‐coordination of both ligands **2** and **5** was confirmed by their molecular structure obtained from X‐ray structure analysis of suitable single crystals (see Figure 11 and 12) and the unhindered rotation of the imidazolium substituents in complex **17** is observed even down at −80 °C, as it is also observed for the free ligand **5**.

Compared to the free ligands the exocyclic Imi−Cp bonds are longer in the complexes. In complex **16** it measures 1.454(2) Å compared to the exocyclic bond of the free ligand **2** of 1.430(3) Å.^[11]^ In complex **17** the exocyclic bonds C1−C6 and C3−C13 of 1.453(2) and 1.457(3) Å are also longer than in the free ligand **5** of 1.440(3) and 1.439(3) Å. This might be caused by a higher single bond character but, what is more plausible, by the weaker Coulomb attraction of the charges due to coordination of the Cp‐ring to the metal.

We tried to synthesize complex **18**, bearing the planar 1,2‐disubstituted ligand **9**, in the same way as complexes **16** and **17**. However, in NMR experiments we recognized only partial conversion to the metal complex **18**, so that an equilibrium mixture containing also the free ligand **9**, acetonitrile and [Ru(CD_3_CN)_3_Cp*](PF_6_) is formed. To shift this equilibrium to the product side, we changed the solvent to dichloromethane so that complex **18** precipitated as a yellow crystalline solid during the reaction. To confirm the existence of such an equilibrium, we isolated the crystals and redissolved them in [D_3_]acetonitrile. After 30 min the formation of 4 % the free metal precursor [Ru(CD_3_CN)_3_Cp*](PF_6_) was observed in the ^1^H NMR spectrum. After 25 h the spectrum shows the final equilibrium ratio with 22 % of [Ru(CD_3_CN)_3_Cp*](PF_6_), which was confirmed after 9 days. Obviously, the monocationic ligand **9** binds much weaker to the metal fragment than the monocationic ligand **5**. In contrast to complex **17**, complex **18** is air sensitive and decomposes over time, most likely due to hydrolysis. All ruthenocenes **16**–**18** were observed in the ESI–MS spectrum using a solution of complexes **16** and **17** in acetonitrile and a solution of **18** in dichloromethane. Only in the case of complex **18** the free ligand **9** is observed in high intensity. This also confirms the weak ligand‐metal bond and the poor donor property of ligand **9**.

**Figure 13:**
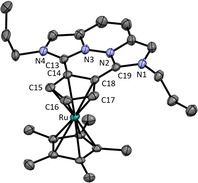
ORTEP plot of the molecular structure of **18** (anisotropic atomic displacement parameters at 50 % probability). Hydrogen atoms, one acetonitrile molecule and two PF_6_^−^ anions are omitted for clarity. Selected bond lengths [Å] and angles [°]: C13−C14 1.432(3), C18−C19 1.436(3), C14−C15 1.427(3), C15−C16 1.415(3), C16−C17 1.424(3), C17−C18 1.423(3), C14−C18 1.472(3), Ru1−C14 2.165(2), Ru1−C15 2.189(2), Ru1−C16 2.200(2), Ru1−C17 2.216(2), Ru1−C18 2.198(2); N1‐C19‐N2 104.2(2), N3‐C13‐N4 104.5(2), N1‐C19‐C18‐C17 5.1, N4‐C13‐C14‐C15 1.9.

The molecular structure of complex **18**, obtained from X‐ray structure analysis, confirms the η^5^‐coordination mode of **9** (see Figure 13). The Imi−Cp bonds of the metal complex **18** (1.432(3) and 1.436(3) Å) are longer compared to those of the free ligand **9** (1.414(3) and 1.418(3) Å). This can be explained again with the reduced Coulomb attraction.

In all solid state structures, the carbenium ion is not bent to the metal center, as it would be the case if further stabilization was required,[^30^, ^31^] but rather points away from it for steric reasons. This behavior was already observed in the ferrocene complex bearing a derivative of **2**.^[11]^ The effect is stronger for **16** and **17**, in which the imidazolium substituents are freely rotating than for complex **18** bearing the rigid ligand **9**. But in all cases, it confirms again the good stabilization of the carbenium ions.

Attempts to form the corresponding ruthenocene with the tri(imidazolium) substituted Cp ligands **12** and **13** in dichloromethane failed. Due to the double positive charge of these ligands as well as the weak electron density in the Cp ring, the acetonitrile ligands cannot be replaced by these Cp compounds.

### Recycling of the air stable ligand 9

As **18** is not air‐stable, it is possible to recover the free ligand by dissolving complex **18** in acetone under air and stir the mixture for several days. Once the complex is decomposed, the formed precipitate can be filtered off and the ligand can be isolated from the filtrate in crystalline form using the same workup procedure as described in the synthesis of ligand **9**. Primarily this is important to reduce the use of thallium salts significantly.

### Side product 20 from the synthesis of [(C_6_H_6_)RuCp*](PF_6_) (19)

**Scheme 8:**
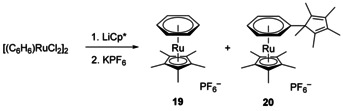
Unexpected formation of the ruthenium benzene complex **20**.

For the following electrochemical investigations we synthesized the literature known [(C_6_H_6_)RuCp*](PF_6_) (**19**) starting from [(C_6_H_6_)RuCl_2_]_2_. Theoretically, this synthesis should require only a salt metathesis with LiCp* however, there is no report on the use of LiCp* in the literature for this example. Instead, TlCp*[^32^, ^33^] and (*n*Bu)_3_SnCp*^[34]^ were used as Cp* source, most likely to avoid the nucleophilic attack at the coordinated benzene.[^33^, ^35^, ^36^, ^37^] Inspired by the literature known reaction with (*n*Bu)_3_SnCp*,^[34]^ we changed the solvent to tetrahydrofuran and added 2 equivalents of LiCp* relative to [(C_6_H_6_)RuCl_2_]_2_ and stirred the mixture at room temperature for 2 h (see Scheme 8). For the workup, we substituted excess NH_4_PF_6_ by 2 equivalents of KPF_6_. Although the formation and isolation of the desired benzene complex **19** in high purity proved successful, the yield of 12 % was significantly lower than reported for the other methods (31 %). Examination of the mother liquor after precipitation of the Ru complex by NMR revealed a second well‐defined compound with a similar solubility. Complex **20** could not be fully separated from **19**, but unambiguously characterized by NMR spectroscopy, X‐ray structure analysis (see Supporting Information) and ESI‐MS (*m*/*z=*449.2 in acetonitrile).

Presumably, complex **20** is formed by nucleophilic attack of LiCp* at the benzene ligand of either the starting material or complex **19**. In both cases a cyclohexadienyl anion should be formed. These complexes are already known from literature.[^33^, ^34^, ^35^, ^36^, ^37^] In our case however, a hydride abstraction is followed so that the benzene ligand is rearomatized, whereas the cyclopentadiene fragment cannot gain back its aromaticity.

### Electrochemical investigations

The electrochemical behavior of pentamethylruthenocene (**15**), the imidazolium‐substituted ruthenocene derivates **16**–**18** and the cationic pentamethylcyclopentadienylbenzeneruthenium complex (**19**) as well as the free ligands **2**, **5** and **9** has been investigated by cyclic voltammetry (CV) in dichloromethane and acetonitrile. Three different electrolytes were used, based on tetrabutylammonium hexafluorophosphate (NBu_4_PF_6_; both solvents) or lithium tetrakis(pentafluorophenyl)borate (Li[B(C_6_F_5_)_4_] (containing 2.5 equiv. Et_2_O; dichloromethane only).

**Figure 14:**
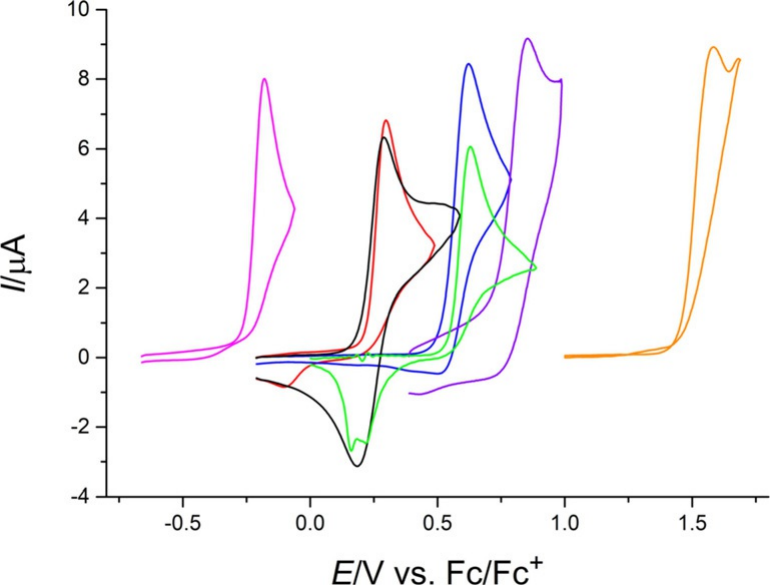
Cyclic voltammograms of **2** (magenta, 0.26 mm), **5** (red, 0.30 mm), **9** (green, 0.28 mm), **15** (black, 0.30 mm), **16** (blue, 0.26 mm), **17** (purple, 0.34 mm) and **19** (orange, 0.29 mm); DCM/0.1 m NBu_4_PF_6_, *v*=0.1 V s^−1^.

Complexes **15**–**17** and **19** exhibit a chemically irreversible anodic oxidation at scan rates of 0.05 to 2 V s^−1^ with the supporting electrolyte NBu_4_PF_6_ in dichloromethane (Figure 14) or acetonitrile. Traditional anions, such as PF_6_^−^, that may interact coordinatively with redox active species have been shown to be responsible for promoting a two‐electron ruthenocene oxidation process by anion attack at the strongly electrophilic ruthenocene(III) center.^[38]^ A similar mechanism might operate here. In both electrolytes, oxidation of ligand **2** is also chemically irreversible. In contrast, for ligands **5** and **9** the shapes of the voltammograms indicate ECE‐type mechanisms. Here, the primary oxidation products undergo a chemical reaction and the resulting species can be reduced. The identity of these intermediates was not further investigated. Only **15** shows a reverse peak directly connected to the primary oxidation in dichloromethane, as indicated by the peak potentials. With a peak current ratio *I*_p_^red^/*I*_p_^ox^ of 0.6 and a peak potential difference Δ*E*_p_ >100 mV at a scan rate of 0.1 V s^−1^ (78 mV at 0.05 V s^−1^), we assume a quasi‐reversible electron transfer step followed by a chemical reaction. Voltammograms of **15** in the acetonitrile electrolyte are comparable to **5** and **9**, thus again indicating an ECE‐mechanism.

**Figure 15:**
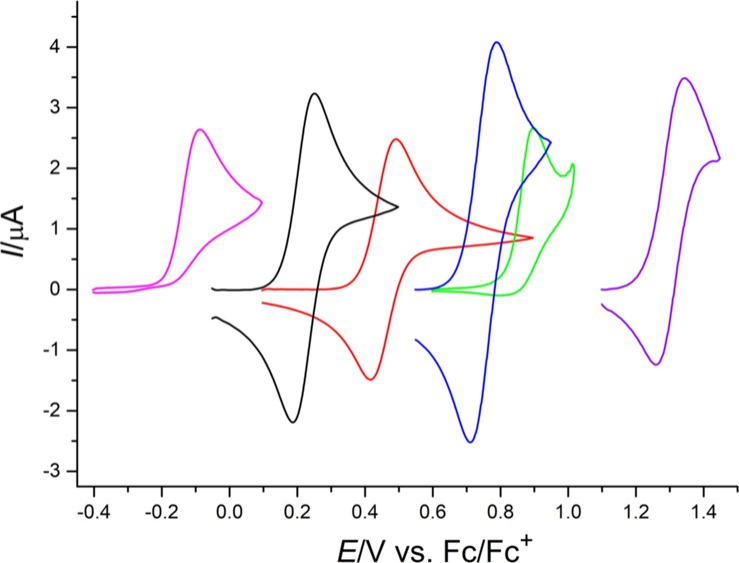
Cyclic voltammograms of **2** (magenta, 0.14 mm), **5** (red, 0.14 mm), **9** (green, 0.13 mm), **15** (black, 0.15 mm), **16** (blue, 0.2 mm) and **17** (purple, 0.2 mm); DCM/0.008 m Li[B(C_6_F_5_)_4_], *v*=0.1 V s^−1^.

**Figure 16:**
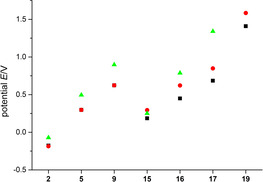
*E*_p_^ox^ of **2**, **5**, **9**, **15**, **16**, **17**, and **19**; CH_3_CN/0.1 m NBu_4_PF_6_ (black), DCM/0.1 m NBu_4_PF_6_ (red), DCM/0.008 m Li[B(C_6_F_5_)_4_] (green).

The interaction between the oxidation product and the PF_6_^−^ anion might be avoided if Li[B(C_6_F_5_)_4_] with its weakly coordinating anion were used. The PF_6_^−^ counter anion of compounds **5** and **16**–**19** was removed by in situ precipitation of solid LiPF_6_ in dichloromethane. The replacement of the anion leads indeed to the occurrence of reverse peaks for complexes **15**, **16** and **17** (Figure 15). The voltammograms of ligands **2** and **9** keep their irreversible shape. The peak potentials of ligands **2**, **5**, and **9**, as well of the complexes **15**, **16** and **17** depend on the electrolyte composition, with most positive potentials for the B(C_6_F_5_)_4_^−^ electrolyte (Figure 16). At the same time, there is a clear trend to more positive potentials for more electron deficient species, that is, those bearing a larger number of imidazolium substituents, in the two series of compounds. Note, that peak potentials of even partially irreversible peaks have a thermodynamic and a kinetic component. The thermodynamically more meaningful formal potentials of compounds **15**, **16** and **17** will be discussed below. Cyclic voltammograms of **18** show an intense, symmetric oxidation peak above 1.57 V, with a peak current that is independent of the starting concentration *c* ^0^. A reduction peak is not found. During multiple potential cycles, *I*_p_^ox^ decreases and *E*_p_^ox^ shifts. We assume adsorption of the compound and progressive deactivation of the electrode surface. Further analysis of the data was not attempted. Complex **19** does not show any electrochemical activity in the B(C_6_F_5_)_4_^−^ electrolyte between −2.3 and 1.7 V. From the oxidation peak potentials in Figure 16 we estimate a value for **19** under these conditions more positive than the accessible potential range of the electrolyte. Thus, **5** (in complex **17**) with two imidazolium substituents seems still to be a better donor ligand than benzene (in **19**).

The oxidation current of precursor ligands **2** and **5** decreases during subsequent potential cycles until the electrochemical activity disappears, possibly due to slow formation of the respective half‐sandwich complexes of lithium and lithiocenes (see Scheme 2 for compound **2**). This could be promoted by the fact that the Li salt is used in excess for the measurement. Another decomposition pathway could be the protonation of the corresponding Cp ring by HCl generated from decomposing dichloromethane.

**Figure 17:**
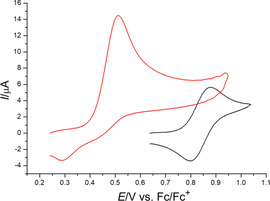
Cyclic voltammograms of **16** (0.26 mm) in DCM/ 0.008 m Li[B(C_6_F_5_)_4_] before (black) and after (red) adding of NBu_4_PF_6_ to the solution.

Addition of NBu_4_PF_6_ in a concentration equimolar to the B(C_6_F_5_)_4_^−^ supporting electrolyte in dichloromethane changes the electrochemical behaviour back to an irreversible two‐electron process, (for example **16**, Figure 17). The negative potential shift could be a result of a coupled follow‐up reaction or increased ion pairing of the oxidation product with PF_6_^−^, as it was proposed by Geiger et al.^[39]^

At 0.05≤*v*≤0.2 V s^−1^, the peak potentials of **15**, **16**, and **17** in the B(C_6_F_5_)_4_^−^ electrolyte are independent of *v* and the concentration *c* ^0^, with Δ*E*_p_ around 70 mV. At higher scan rates Δ*E*_p_ increases moderately, indicating the influence of electrochemical quasireversibility (electron‐transfer kinetics) at these faster time scales. Concentration normalized currents *I*_p_/*c* ^0^ are almost independent of *c* ^0^ and increase linearly with the square root of the scan rate. Thus, the electrode reaction is diffusion controlled. The peak current ratio *I*_p_^red^/*I*_p_^ox^ is close to unity for *v* between 0.05 and 2 V s^−1^, indicating chemical reversibility, that is, the absence of follow‐up reactions of the primary oxidation products. Under conditions where the processes are chemically and electrochemically reversible (or mildly quasi‐reversible), formal potentials *E* ^0^ of **15**, **16**, and **17** were determined from the cyclic voltammograms as the „mid‐point potentials“ and confirmed from differential pulse voltammetry (DPV) data (Table 1).

**Table 1:** *E* ^0^ of **15**, **16** and **17** vs. Fc/Fc^+^; DCM/0.008 m Li[B(C_6_F_5_)_4_].

*E* ^0^/V	**15**	**16**	**17**
CV	0.219±0.003	0.749±0.002	1.299±0.002
DPV	0.219±0.001	0.750±0.002	1.301±0.003

We conclude that every additional imidazolium substituent increases the oxidation potential of ruthenocene complexes from **15** to **16** to **17** by more than 500 mV owing to its electron‐withdrawing effect and insertion of an additional positive charge.

While none of the compounds could be reduced in the accessible potential range of DCM, **5**, **9**, **16**, **17** and **19** showed reduction peaks at very negative potentials in MeCN/ 0.1 m NBu_4_PF_6_. The reduction of the complexes under these conditions is irreversible, possibly owing to the high reactivity of a primarily formed 19‐electron ruthenocene. The values for *E*_p_^red^ (**5**: −3.145 V, **9**: −2.244 V, **16**: −2.890 V, **17**: −2.775 V, **19**: −2.599 V; see Supporting Information) are consistent with the substituent effect and the decrease of the electron density in this order.

## Conclusions

In this work we improved the synthesis of imidazolium cyclopentadienylides like **2** and introduced di(imidazolium)‐ (**5** and **9**) and even tri(imidazolium) cyclopentadienylides (**12** and **13**). The electron‐withdrawing effect of the imidazolium substituent could already be noticed in the synthesis of the compounds and comparing their reactivity against air and moisture. The different reactivity manifests itself also in the synthesis of the ruthenocenes of the mono substituted ylide **16** and of the disubstituted ylides **17** and **18**. Complexes of the trisubstituted ylides could not be prepared. The electrochemical data support the electron withdrawing substituent effect for the ligands and the complexes. The experimental data and DFT calculations show a pronounced difference between free rotating and planar fixed imidazolium moieties. We also could observe a rare equilibrium between a coordinated and a non‐coordinated Cp ligand, as the metal Cp bond is weakened due to the lower electron density of the ligand **9**. The trisubstituted compounds **12** and **13** provide even less electron density in the Cp ring which prevents coordination to the ruthenium centre.

Currently we are investigating the CO stretching frequencies of the corresponding half‐sandwich tricarbonyl complexes of chromium(0), molybdenum(0) and tungsten(0) to quantify the donor properties of our novel ligands.

## Experimental Section

***General remarks***: All experiments, if not other otherwise noted, were performed under inert argon atmosphere using standard Schlenk technique. Solvents were dried and degassed using an MB‐SPS Solvent Purification System from MBraun. Deuterated solvents were dried with standard purification methods and degassed⋅^[40]^ The NMR spectra were recorded at 26 °C with a Bruker DRX‐250, Bruker AVII+400 spectrometer, a Bruker AVII+500, a Bruker AVII HDX 600 and with a Bruker Avance III HDX 700. ^1^H and ^13^C{^1^H} NMR spectra were referenced to the respective solvent signal as the internal standard. For ^19^F{^1^H} NMR pure CFCl_3_ and for ^31^P{^1^H} NMR 85 % aqueous H_3_PO_4_ was used as external standard. The ^7^Li{^1^H} NMR chemical shifts are calibrated to a 1 m LiCl solution in water as the external standard. Signals were assigned using 2D NMR (COSY, NOESY, HSQC, HMBC) experiments and reported in ppm. The numbering scheme of the signals is depicted in the Supporting Information. A slight deviation of the integrals of the Cp protons compared to the methyl groups can be observed in the ^1^H NMR spectrum of the ligands. This is not due to H/D exchange with deuterated solvents, but to the different relaxation times of the protons. Changing the delay time from 1 s to 60 s leads to matching of observed and theoretical integral values. The IR spectra were collected with a Bruker Vertex 70. The elemental analyses were determined using a varioMICRO cube by the elemental analysis section of the Institut für Anorganische Chemie at the University of Tübingen and mass spectra were recorded using a Bruker Esquire 3000 Plus by the mass spectra section of the Institut für Organische Chemie at the University of Tübingen. Melting points were measured using a Büchi Melting Point M‐560. UV/VIS spectra were recorded with a Jasco V‐770 UV/Visible/NIR spectrophotometer. 1,3,4,5‐tetramethylimidazolin‐2‐ylidene,^[20]^ Cp*H,^[41]^ [RuCp*Cl_2_]_*n*_,^[42]^ [RuCp*(CH_3_CN)_3_](PF_6_),^[43]^ thallium cyclopentadienide,^[44]^ *N*,*N’*‐di(*n*‐propyl)‐1,8‐dichlorodiimidazo[1,5‐*b*:5’,1’‐*f*]pyridazinium hexafluorophosphate (**8**),[^27^, ^45^] B(C_6_F_5_)_3_[^44^, ^46^, ^47^, ^48^] and Li[B(C_6_F_5_)_4_]⋅2.5Et_2_O^[49]^ were synthesized according to the published procedure. *n*BuLi, dicyclopentadiene, hexachloroethane, lithium bis(trimethylsilyl)amide, potassium hexafluorophosphate and deuterated water were purchased and used without further purification.

***Electrochemical investigations***: Dichloromethane and acetonitrile for electrochemical experiments were purchased from Alfa Aesar. Dichloromethane was distilled over P_2_O_5_ and then over K_2_CO_3_. Acetonitrile was distilled successively over P_2_O_5_, CaH_2_ and again over P_2_O_5_. Silver perchlorate (AgClO_4_), tetra‐*n*‐butylammonium hexafluorophosphate (NBu_4_PF_6_) and ferrocene were purchased from Alfa Aesar (reagent grade). The supporting electrolytes NBu_4_PF_6_ were used in 0.1 m (NBu_4_PF_6_) and in 0.008 m (LiB(C_6_F_5_)_4_) concentration. The electrolyte solutions were degassed by freeze‐pump‐thaw cycles (CH_2_Cl_2_) or by bubbling of argon (acetonitrile). For the electrochemical experiments, an Eco Chemie BV Autolab PG‐STAT100 (Metrohm, Filderstadt, Germany) was used with control software GPES (v. 4.9). Cyclic staircase voltammograms were recorded with *α*=0.5 defining the sampling point at 17 °C with a glassy carbon electrode. All experiments were carried out under argon with a gas‐tight full‐glass cell in a three‐electrode arrangement.^[50]^ *iR* drop was compensated by positive feedback through the GPES software. Cyclic voltammetric scan rates ranged from 0.05 to 2 V s^−1^. All CV experiments were carried out for several concentrations. Background curves were recorded for various scan rates in the first part of an experimental session for later use. Then the substrate was added in the form of several aliquots from a stock solution. *i*‐*E*‐curves were recorded at all scan rates after each addition. Finally, background currents were subtracted from these data. Differential pulse voltammograms were recorded with a pulse height of 5 mV. Formal potentials were determined as mean values of peak potentials („mid‐point potentials“) from cyclic voltammograms of reversible shape, or from the peak potentials in differential pulse voltammograms after correction by half the pulse height. All potentials were determined vs. a Ag/Ag^+^ (0.01 m in CH_3_CN/0.1 m NBu_4_PF_6_) electrode with a Haber‐Luggin dual reference electrode system.^[51]^ The values were rescaled to the Fc/Fc^+^ standard for each electrolyte (*E* ^0^ ${}_{\mathrm{DCM}/\mathrm{NBu}{}_{4}\mathrm{PF}{}_{6}}$ (Fc/Fc^+^)=212±1 mV, *E* ^0^ ${}_{\mathrm{MeCN}/\mathrm{NBu}{}_{4}\mathrm{PF}{}_{6}}$ (Fc/Fc^+^)=82±1 mV, *E* ^0^ ${}_{\mathrm{DCM}/\mathrm{LiB}\left(C{}_{6}F{}_{5}\right){}_{4}}$ (Fc/Fc^+^)=302±3 mV, all vs. Ag/Ag^+^).

***Modified synthesis of lithium cyclopentadienide***:^[52]^ To a solution of *n*BuLi (1.6 m, 50 mL, 80 mmol, 1 equiv) in *n*‐hexane at 0 °C a solution of freshly distilled CpH (9.9 mL, 0.12 mol, 1.5 equiv) in 100 mL of pentane was added dropwise under stirring within 1 h. After the addition was completed, the suspension was stirred for 30 min at room temperature. Then the residue was filtered off and washed three times with 20 mL of pentane. After drying in vacuo, the product (5.5 g, 77 mmol, 96 %) was obtained as a white powder. ^1^H NMR (400 MHz, [D_6_]DMSO): *δ* [ppm]=5.32 (s, Cp‐H). ^1^H NMR (400 MHz, CD_3_CN): *δ* [ppm]=5.51 (s, Cp‐H). ^1^H NMR (400 MHz, [D_8_]THF): *δ* [ppm]=5.68 (s, Cp‐H). ^1^H NMR (400 MHz, [D_8_]toluene: [D_8_]THF 11:1): *δ* [ppm]=6.32 (s, Cp‐H). ^1^H NMR (400 MHz, C_6_D_6_: [D_8_]THF 8:1): *δ* [ppm]=6.46 (s, Cp‐H).

***Modified synthesis of 2‐chloro‐1,3,4,5‐tetramethylimidazolium chloride (1)***:[^21^, ^22^] To a solution of hexachloroethane (1.19 g, 5.03 mmol, 1.25 equiv) in 20 mL of diethyl ether at −30 °C a solution of 1,3,4,5‐tetramethylimidazolin‐2‐ylidene (500 mg, 4.03 mmol, 1 equiv) in 40 mL diethyl ether was added dropwise over a period of 30 min. The suspension was stirred for additional 20 min at room temperature. After filtration the residue was washed with 40 mL of diethyl ether and dried in vacuo. The product was obtained as colorless microcrystals (0.713 g, 3.65 mmol, 91 %). Single crystals suitable for X‐ray analysis were obtained by diffusion of diethyl ether into a solution of acetonitrile at −30 °C. ^1^H NMR (400 MHz, CD_3_CN): *δ* [ppm]=3.67 (s, 6 H, NCH_3_), 2.26 (s, 6 H, CCH_3_). ^13^C{^1^H} NMR (100 MHz, CD_3_CN): *δ* [ppm]=130.1 (CCl, signal from HMBC), 128.8 (CCH_3_), 33.9 (NCH_3_), 9.3 (CCH_3_). CHN: calcd C 43.10, H 6.20, N 14.36 found C 43.25, H 5.93, N 14.47. MS (ESI^+^, CH_3_CN): *m*/*z=*159.0 [M]^+^.

***Modified synthesis of 1,3,4,5‐tetramethylimidazolium cyclopentadienide (2)***:^[11]^ To a suspension of LiCp (488 mg, 6.77 mmol, 2.10 equiv) in 15 mL of acetonitrile at room temperature a solution of **1** (629 mg, 3.23 mmol, 1 equiv) in 5 mL of acetonitrile was added under stirring. The suspension was stirred for 1 h, whereupon the color turned brownish and a white precipitate formed, which was filtered off. The filtrate was dried in vacuo and extracted three times with each 7 mL of dichloromethane. The combined organic layers were washed three times with 5 mL of degassed water and then concentrated to dryness. The residue was washed with 15 mL of pentane and dried in vacuo to obtain the product as a lilac solid (517 mg, 2.74 mmol, 85 %). ^1^H NMR (400 MHz, [D_6_]DMSO): *δ* [ppm]=6.04–6.02 (m, 2 H, 2/5‐H), 5.84–5.82 (m, 2 H, 3/4‐H), 3.63 (s, 6 H, NCH_3_), 2.18 (s, 6 H, CCH_3_). ^1^H NMR (400 MHz, CD_3_CN): *δ* [ppm]=6.14–6.12 (m, 2 H, 2/5‐H), 5.94–5.93 (m, 2 H, 3/4‐H), 3.66 (s, 6 H, NCH_3_), 2.17 (s, 6 H, CCH_3_). ^1^H NMR (400 MHz, CD_2_Cl_2_): *δ* [ppm]=6.21–6.20 (m, 2 H, 2/5‐H), 6.05–6.04 (m, 2 H, 3/4‐H), 3.70 (s, 6 H, NCH_3_), 2.17 (s, 6 H, CCH_3_). ^1^H NMR (400 MHz, [D_8_]THF): *δ* [ppm]=6.08–6.07 (m, 2 H, 2/5‐H), 5.93–5.91 (m, 2 H, 3/4‐H), 3.66 (s, 6 H, NCH_3_), 2.17 (s, 6 H, CCH_3_). ^1^H NMR (400 MHz, CDCl_3_): *δ* [ppm]=6.35–6.34 (m, 2 H, 2/5‐H), 6.31–6.29 (m, 2 H, 3/4‐H), 3.76 (s, 6 H, NCH_3_), 2.20 (s, 6 H, CCH_3_). ^1^H NMR (400 MHz, [D_8_]toluene): *δ* [ppm]=6.92–6.91 (m, 2 H, 3/4‐H), 6.54–6.52 (m, 2 H, 2/5‐H), 3.00 (s, 6 H, NCH_3_), 1.14 (s, 6 H, CCH_3_). ^1^H NMR (400 MHz, C_6_D_6_): *δ* [ppm]=7.14–7.12 (m, 2 H, 3/4‐H), 6.69–6.67 (m, 2 H, 2/5‐H), 2.98 (s, 6 H, NCH_3_), 1.25 (s, 6 H, CCH_3_). CHN: calcd C 76.55, H 8.57, N 14.88 found C 76.63, H 8.30, N 14.56. MS (ESI^+^, CH_3_CN): *m*/*z=*189.1 [*M*+H]^+^. UV/VIS (CH_3_CN): *λ*_1_=332 nm (*ϵ*=2.5×10^4^ L mol^−1^⋅cm^−1^), *λ*_2_=491 nm (*ϵ*=1×10^1^ L mol^−1^⋅cm^−1^). Contrary to earlier observations the compound was stable over months under argon at rt.

***Formation of the lithium adducts 3 and 4***: Li[B(C_6_F_5_)_4_]⋅2.5Et_2_O (18 mg, 21 μmol, 1 equiv) and **2** (5.0 mg, 27 μmol, 1.3 equiv) were dissolved in 0.4 mL of [D_2_]dichloromethane. ^1^H NMR (500 MHz, CD_2_Cl_2_, RT): *δ* [ppm]=6.13–6.12 (m, 2 H, 2/5‐H), 6.01–5.99 (m, 2 H, 3/4‐H), 3.68 (s, 6 H, NCH_3_), 2.23 (s, 6 H, CCH_3_). ^7^Li NMR (194 MHz, CD_2_Cl_2_, RT): *δ* [ppm]=0.8 (br s, Li), −7.3 (br s, LiCp). ^7^Li NMR (194 MHz, CD_2_Cl_2_, −80 °C): *δ* [ppm]=−0.8 (s, Li), −6.0 (br s, CpLi (**3**)), −11.7 (s, LiCp_2_ (**4**)).

***Formation of the lithium adducts 3‐NTf***_***2***_: LiNTf_2_ (7.6 mg, 27 μmol, 1 equiv) and **2** (5.0 mg, 27 μmol, 1.0 equiv) were dissolved in 0.4 mL of [D_2_]dichloromethane. ^1^H NMR (250 MHz, CD_2_Cl_2_, RT): *δ* [ppm]=6.24–6.22 (m, 2 H, 2/5‐H), 6.12–6.9 (m, 2 H, 3/4‐H), 3.70 (s, 6 H, NCH_3_), 2.21 (s, 6 H, CCH_3_). ^7^Li NMR (97 MHz, CD_2_Cl_2_, RT): *δ* [ppm]=−7.0 (br s, LiCp).

***Synthesis of 1,3‐bis(1,3,4,5‐tetramethylimidazolium)cyclopentadienide hexafluorophosphate (5)***: A solution of **2** (241 mg, 1.28 mmol, 1 equiv) and **1** (250 mg, 1.28 mmol, 1 equiv) in 8 mL of acetonitrile was stirred at room temperature for 20 h. Then LiHMDS (155 mg, 0.926 mmol, 0.72 equiv) was added to the formed suspension. After 48 h a second portion of LiHMDS (81.0 mg, 0.484 mmol, 0.38 equiv) was added. After stirring the mixture overnight, the solvent was removed in vacuo and the residue suspended in 5 mL of water. The solid was removed by filtration and a concentrated solution of KPF_6_ (259 mg, 1.41 mmol, 1.10 equiv) in water was added under stirring to the filtrate. After stirring for 20 min at rt the precipitate was filtered off and washed with each 10 mL of water and diethyl ether. The residue was then dissolved in 3 mL of acetonitrile and the product precipitated with 15 mL water. After filtration, washing with water and drying in vacuo, the product was obtained as a pale heather, air stable crystalline solid (369 mg, 0.808 mmol, 63 %). Single crystals suitable for X‐ray analysis were obtained by slow evaporation of acetonitrile from a solution of **5**. ^1^H NMR (400 MHz, CD_3_CN): *δ* [ppm]=6.44 (t, ^3^ *J*_HH_=2.3 Hz, 1 H, 2‐H), 6.32 (d, ^3^ *J*_HH_=2.3 Hz, 2 H, 4/5‐H), 3.68 (s, 12 H, NCH_3_), 2.22 (s, 12 H, CCH_3_). ^13^C{^1^H} NMR (100 MHz, CD_3_CN): *δ* [ppm]=147.2 (C‐6/7), 124.9 (CCH_3_), 114.7 (C‐2), 113.4 (C‐4/5), 103.4 (C‐1/3), 33.8 (NCH_3_), 9.1 (CCH_3_). ^19^F{^1^H} NMR (376 MHz, CD_3_CN): *δ* [ppm]=−72.9 (d, ^1^ *J*_PF_=707 Hz, PF_6_). ^31^P{^1^H} NMR (162 MHz, CD_3_CN): *δ* [ppm]=−144.6 (spt, ^1^ *J*_PF_=707 Hz, PF_6_). ^1^H NMR (400 MHz, CD_2_Cl_2_): *δ* [ppm]=6.41 (t, ^3^ *J*_HH_=2.3 Hz, 1 H, 2‐H), 6.34 (d, ^3^ *J*_HH_=2.3 Hz, 2 H, 4/5‐H), 3.72 (s, 12 H, NCH_3_), 2.25 (s, 12 H, CCH_3_). CHN: calcd C 50.00, H 5.96, N 12.28 found C 50.11, H 5.70, N 12.26. MS (ESI^+^, CH_3_CN): *m*/*z=*311.2 [M]^+^. UV/VIS (CH_2_Cl_2_): *λ*_1_=284 nm (*ϵ*=3.0×10^3^ L mol^−1^⋅cm^−1^), *λ*_2_=339 nm (*ϵ*=7.2×10^3^ L mol^−1^⋅cm^−1^), *λ*_3_=524 nm (*ϵ*=1×10^2^ L mol^−1^⋅cm^−1^). UV/VIS (CH_3_CN): *λ*_1_=275 nm (*ϵ*=1.2×10^4^ L mol^−1^⋅cm^−1^), *λ*_2_=336 nm (*ϵ*=3.5×10^4^ L mol^−1^⋅cm^−1^), *λ*_3_=509 nm (*ϵ*=2.5×10^4^ L mol^−1^⋅cm^−1^). IR (KBr): *ṽ* [cm^−1^]=2961 (w), 2929 (w), 1653 (m), 1548 (s), 1504 (m), 1440 (m), 1348 (m), 1245 (w), 1213 (w), 1126 (w), 1047 (w), 910 (m), 840 (vs., PF_6_^−^), 710 (w), 647 (w), 558 (s, PF_6_^−^). Mp.: 266 °C.

***H/D exchange of 5***: To a solution of **5** (23.0 mg, 50.4 μmol, 1 equiv) in 0.4 mL of CD_3_CN 91 μL D_2_O (101 mg, 5.04 mmol, 100 equiv) were added. The mixture was then heated for 5 d at 60 °C. The solvent was removed in vacuo and the residue dissolved in 0.4 mL of CD_3_CN to confirm the formation of **5‐d_3_**. ^1^H NMR (400 MHz, CD_3_CN): *δ* [ppm]=6.45 (s, residual proton signal, 2‐H), 6.32 (s, residual proton signal, 4/5‐H), 3.69 (s, 12 H, NCH_3_), 2.23 (s, 12 H, CCH_3_). ^13^C{^1^H} NMR (100 MHz, CD_3_CN): *δ* [ppm]=147.2 (C‐6/7), 124.9 (CCH_3_), 114.7 (C‐2), 114.4 (t, ^1^ *J*_CD_=24.3 Hz, C‐2), 113.2 (C‐4/5), 113.0 (t, ^1^ *J*_CD_=24.3 Hz, C‐4/5), 103.2 (C‐1/3), 33.8 (NCH_3_), 9.1 (CCH_3_). MS (ESI^+^, CH_3_CN): *m*/*z=*314.2 [M]^+^. MS (ESI^−^, CH_3_CN): *m*/*z=*144.8 [PF_6_]^−^. The deuteration degree for the Cp of 91 % was determined from the ESI–MS spectrum.

***Independent formation of 6 and 7***: To a suspension of **1** (31.4 mg, 161 μmol, 1 equiv) in 0.5 mL of CD_3_CN tetramethylimidazolin‐2‐ylidene (20.0 mg, 161 μmol, 1.00 equiv) was added. After 4 h 2 mL of diethyl ether were added and the solid was filtered off, dissolved in water and combined with a saturated aqueous solution of KPF_6_ (70.0 mg, 380 μmol, 2.36 equiv). The formed off‐white precipitate was filtered off, washed with water and diethyl ether and dried in vacuo. The air stable product is a mixture of **6** and **7** in the ratio 3:2. Single crystals suitable for X‐ray analysis of **6** (colorless plates) and **7** (colorless needles) were obtained by slow evaporation of acetonitrile from a solution of the mixture. Compound **6**: ^1^H NMR (700 MHz, CD_3_CN): *δ* [ppm]=3.26 (s, 6 H, NCH_3_), 2.15 (s, 6 H, CCH_3_). ^13^C{^1^H} NMR (176 MHz, CD_3_CN): *δ* [ppm]=145.2 (NCN), 125.2 (CCH_3_), 121.6 (NCC), 32.9 (NCH_3_), 30.1 (NCC), 9.1 (CCH_3_). ^1^H NMR (700 MHz, CD_2_Cl_2_): *δ* [ppm]=3.33 (s, 6 H, NCH_3_), 2.20 (s, 6 H, CCH_3_). ^13^C{^1^H} NMR (176 MHz, CD_3_CN): *δ* [ppm]=144.5 (NCN), 124.9 (CCH_3_), 120.9 (NCC), 32.7 (NCH_3_), 30.0 (NCC), 9.3 (CCH_3_). MS (ESI^+^, CH_3_CN): *m*/*z=*286.2 [M]^+^. Compound **7**: ^1^H NMR (700 MHz, CD_3_CN): *δ* [ppm]=3.58 (s, 6 H, NCH_3_), 2.36 (s, 6 H, CCH_3_). ^13^C{^1^H} NMR (176 MHz, CD_3_CN): *δ* [ppm]=134.0 (CCH_3_), 124.1 (NCN), 34.8 (NCH_3_), 9.4 (CCH_3_). MS (ESI^+^, CH_3_CN): *m*/*z=*124.1 [M]^2+^, 393.2 [M+PF_6_]^2+^.

***Synthesis of 7***: To a suspension of **2** (100 mg, 513 μmol, 1 equiv) in 2.5 mL tetrahydrofuran at −30 °C was added dropwise a solution of tetramethylimidazol‐2‐ylidene (64.0 mg, 515 μmol, 1.00 equiv) in 2.5 mL tetrahydrofuran. The mixture was stored at −30 °C for 16 h. The solvent was removed in vacuo and the residue dissolved in water. A saturated aqueous solution of KPF_6_ (234 mg, 1.27 mmol, 2.48 equiv) was added under stirring. The formed precipitate was filtered off, washed with 5 mL water, twice with 5 mL diethyl ether each and dried in vacuo to obtain **7** as an air stable off‐white solid (204 mg, 388 μmol, 74 %). ^1^H NMR (400 MHz, CD_3_CN): *δ* [ppm]=3.59 (s, 6 H, NCH_3_), 2.36 (s, 6 H, CCH_3_). ^13^C{^1^H} NMR (100 MHz, CD_3_CN): *δ* [ppm]=134.0 (CCH_3_), 124.2 (NCN, signal from HMBC), 34.8 (NCH_3_), 9.4 (CCH_3_). MS (ESI^+^, CH_3_CN): *m*/*z=*124.1 [M]^2+^, 393.2 [M+PF_6_]^2+^.

***Synthesis of 9***: To a suspension of TlCp (237 mg, 1.01 mmol, 3.05 equiv) in 4 mL acetonitrile a solution of *N*,*N’*‐di(*n*‐propyl)‐1,8‐dichlorodiimidazo[1,5‐*b*:5’,1’‐*f*]pyridazinium di(hexafluorophosphate) (**8**) (200 mg, 0.332 mmol, 1 equiv) in 6 mL of acetonitrile was added. After stirring for 72 h in the dark, the reaction mixture turned yellow and a grey precipitate had formed. The solid was filtered off and the solvent removed in vacuo. The residue was dissolved in 10 mL acetonitrile and 20 mL of degassed water was added to precipitate the yellow raw material, which was filtered off, dissolved in 1.5 mL acetonitrile and 30 mL of ethyl ether were added. The obtained yellow solid was filtered off again, dissolved in 5 mL acetonitrile and 40 mL degassed water were added. The yellow precipitate was filtered off, washed with 3 mL degassed water and dried in vacuo. The product **9** was obtained as a yellow, air‐stable solid (108 mg, 0.240 mmol, 72 %). Crystals suitable for X‐ray analysis were obtained by overlaying a solution of **9** in dichloromethane with pentane. ^1^H NMR (400 MHz, CD_3_CN): *δ* [ppm]=7.16 (s, 2 H, H‐2/5), 6.91 (d, 2 H, ^3^ *J*_HH_=3.8 Hz, H‐7/9), 6.77 (t, 1 H, ^3^ *J*_HH_=3.8 Hz, H‐8), 6.73 (s, 2 H, H‐3/4), 4.29 (t, 4 H, ^3^ *J*_HH_=7.2 Hz, NCH_2_), 1.96 (tq, 4 H, ^3^ *J*_HH_=7.4 Hz, ^3^ *J*_HH_=7.2 Hz, CH_2_), 1.00 (t, 6 H, ^3^ *J*_HH_=7.4 Hz, CH_3_). ^13^C{^1^H} NMR (100 MHz, CD_3_CN): *δ* [ppm]=133.2 (C‐6a/9b), 124.9 (C‐8), 120.8 (C‐2a/4a), 115.9 (C‐3/4), 115.0 (C‐2/5), 111.9 (C‐7/9), 104.7 (C‐6b/9a), 51.2 (NCH_2_), 23.7 (CH_2_), 11.0 (CH_3_). ^19^F{^1^H} NMR (376 MHz, CD_3_CN): *δ* [ppm]=−72.9 (d, ^1^ *J*_PF_=707 Hz, PF_6_). ^31^P{^1^H} NMR (162 MHz, CD_3_CN): *δ* [ppm]=−144.6 (spt, ^1^ *J*_PF_=707 Hz, PF_6_). ^1^H NMR (400 MHz, CD_2_Cl_2_): *δ* [ppm]=7.08 (s, 2 H, H‐2/5), 6.93–6.89 (m, 3 H, H‐7/9 and H‐8), 6.73 (s, 2 H, H‐3/4), 4.29 (t, 4 H, ^3^ *J*_HH_=7.3 Hz, NCH_2_), 2.01 (tq, 4 H, ^3^ *J*_HH_=7.4 Hz, ^3^ *J*_HH_=7.3 Hz, CH_2_), 1.08 (t, 6 H, ^3^ *J*_HH_=7.4 Hz, CH_3_). CHN: calcd C 50.67, H 4.70, N 12.44 found C 50.64, H 4.95, N 12.18. MS (ESI^+^, CH_3_CN): *m*/*z=*305.2 [M]^+^. MS (ESI^−^, CH_3_CN): *m*/*z=*144.8 [PF_6_]^−^. UV/VIS (CH_3_CN): *λ*_1_=284 nm (*ϵ*=2.2×10^4^ L mol^−1^⋅cm^−1^), *λ*_2_=339 nm (*ϵ*=1.1×10^4^ L mol^−1^⋅cm^−1^). IR (KBr): *ṽ* [cm^−1^]=2971 (w), 2920 (w), 1630 (m), 1587 (m), 1459 (w), 1386 (w), 1308 (w), 1343 (w), 1308 (w), 1160 (w), 1047 (w), 1024 (w), 841 (vs., PF_6_^−^), 792 (w), 733 (w), 722 (w), 558 (s, PF_6_^−^). Mp.: 202 °C (dec.).

***Synthesis of 9‐d***_***2***_: To a solution of **9** (23.0 mg, 51.1 μmol, 1 equiv) in 0.4 mL of CD_3_CN 92 μL D_2_O (102 mg, 5.11 mmol, 100 equiv) were added. The mixture was then heated for 24 h at 60 °C. The solvent was removed in vacuo and the residue dissolved in 0.4 mL of CD_3_CN and the solution filtered. ^1^H NMR (400 MHz, CD_3_CN): *δ* [ppm]=7.16 (s, residual proton signal, H‐2/5), 6.89 (d, 2 H, ^3^ *J*_HH_=3.8 Hz, H‐7/9), 6.76 (t, 1 H, ^3^ *J*_HH_=3.8 Hz, H‐8), 6.72 (s, 2 H, H‐3/4), 4.28 (t, 4 H, ^3^ *J*_HH_=7.2 Hz, NCH_2_), 1.95 (tq, 4 H, ^3^ *J*_HH_=7.4 Hz, ^3^ *J*_HH_=7.2 Hz, CH_2_), 1.00 (t, 6 H, ^3^ *J*_HH_=7.4 Hz, CH_3_). ^13^C{^1^H} NMR (100 MHz, CD_3_CN): *δ* [ppm]=133.1 (C‐6a/9b), 124.9 (C‐8), 120.7 (C‐2a/4a), 115.8 (C‐3/4), 115.0 (C‐2/5), 114.8 (t, ^1^ *J*_CD_=31.2 Hz, C‐2/5), 111.9 (C‐7/9), 104.7 (C‐6b/9a), 51.2 (NCH_2_), 23.7 (CH_2_), 11.0 (CH_3_). MS (ESI^+^, CH_3_CN): *m*/*z=*307.2 [M]^+^. The deuteration degree of 93 % was determined from the ESI‐spectrum.

***Synthesis of 12 and 13***: To a solution of *N*,*N’*‐di(*n*‐propyl)‐1,8‐dichlorodiimidazo[1,5‐*b*:5’,1’‐*f*]pyridazinium di(hexafluorophosphate) (**7**) (118 mg, 195 μmol, 1 equiv) in 2 mL acetonitrile, a solution of **2** (36.7 mg, 195 μmol, 1 equiv) in 2 mL acetonitrile is added upon which the color turned orange. After stirring for 1 h LiHMDS (65.2 mg, 390 μmol, 2 equiv) was added as solid together with 2 mL acetonitrile. After stirring for 1 h at room temperature, the dark orange solution was concentrated in vacuo to 2 mL and 10 mL degassed water were added. After stirring for 30 min, the formed brown precipitate was filtered off and dissolved in 2 mL of acetonitrile. 30 mL of diethyl ether were added to precipitate the product, which was filtered off, washed with 5 mL diethyl ether and dried in vacuo. The brown air stable product (84.2 mg, 117 μmol, 60 %) is a mixture of both isomers **12** and **13** in 4.5:1 ratio. Crystals suitable for X‐ray analysis of **12** (orange plates) and **13** (oranges blocks) were obtained by diffusion of diethyl ether into a solution of the product mixture in acetonitrile. **12**: ^1^H NMR (600 MHz, CD_3_CN): *δ* [ppm]=7.35 (s, 2 H, H‐2/5), 7.09 (s, 2 H, H‐7/9), 6.87 (s, 2 H, H‐3/4), 4.37 (t, ^3^ *J*_HH_=7.0 Hz, 4 H, NCH_2_), 3.65 (s, 6 H, NCH_3_), 2.30 (s, 6 H, CCH_3_), 1.96 (tq, 4 H, ^3^ *J*_HH_=7.4 Hz, ^3^ *J*_HH_=7.0 Hz, CH_2_), 1.00 (t, 6 H, ^3^ *J*_HH_=7.4 Hz, CH_3_). ^13^C{^1^H} NMR (151 MHz, CD_3_CN): *δ* [ppm]=143.2 (C‐10), 132.9 (C‐6a/9b), 127.4 (CCH_3_), 121.3 (C‐2a/4a), 116.3 (C‐3/4), 116.1 (C‐2/5), 116.0 (C‐6b/9a), 113.1 (s, C‐7/9), 106.2 (C‐8), 51.6 (NCH_2_), 34.0 (NCH_3_), 23.7 (CH_2_), 10.9 (CH_3_), 9.1 (CCH_3_). **13**: ^1^H NMR (600 MHz, CD_3_CN): *δ* [ppm]=7.34 (s, 1 H, H‐2), 7.24 (s, 1 H, H‐5), 7.12 (d, 1 H, ^3^ *J*_HH_=4.2 Hz, H‐9), 6.84 (d, 1 H, ^3^ *J*_HH_=4.2 Hz, H‐8), 6.83 (s, 1 H, H‐3 or H‐4), 6.80 (s, 1 H, H‐3 or H‐4), 4.40–4.37 (m, 2 H, NCH_2_^b^), 3.50 (s, 6 H, NCH_3_), 3.18–3.15 (m, 2 H, NCH_2_^a^), 2.33 (s, 6 H, CCH_3_), 2.02–1.96 (m, 2 H, CH_2_^b^), 1.59–1.53 (m, 2 H, CH_2_^a^), 1.04 (t, 3 H, ^3^ *J*_HH_=7.3 Hz, CH_3_^b^), 0.71 (t, 3 H, ^3^ *J*_HH_=7.3 Hz, CH_3_^a^). ^13^C{^1^H} NMR (151 MHz, CD_3_CN): *δ* [ppm]=142.6 (C‐10), 128.6 (CCH_3_), 127.9 (C‐8), 116.5 (C‐3 or C‐4), 116.3 (C‐3 or C‐4), 116.2 (C‐2), 116.1 (C‐5), 112.7 (C‐9), 51.6 (NCH_2_^b^), 51.6 (NCH_2_^a^), 33.7 (NCH_3_), 23.7 (CH_2_^b^), 23.4 (CH_2_^a^), 10.9 (CH_3_^b^), 10.8 (CH_3_^a^), 9.1 (CCH_3_). The signals for C‐2a, C‐4a, C‐6a, C‐6b, C‐7, C‐9a and C‐9b could not be assigned unambiguously. Both: ^19^F{^1^H} NMR (376 MHz, CD_3_CN): *δ* [ppm]=−72.9 (d, ^1^ *J*_PF_=707 Hz, PF_6_). ^31^P{^1^H} NMR (162 MHz, CD_3_CN): *δ* [ppm]=−144.6 (spt, ^1^ *J*_PF_=707 Hz, PF_6_). MS (ESI^+^, CH_3_CN): *m*/*z=*214.0 [M]^2+^. MS (ESI^−^, CH_3_CN): *m*/*z=*144.8 [PF_6_]^−^.

***Synthesis of 12‐d***_***2***_ ***and 13‐d***_***2***_: To a solution of the above obtained mixture of **12** and **13** (20.0 mg, 27.8 μmol, 1 equiv) in 0.4 mL of CD_3_CN were added 100 μL D_2_O (111 mg, 5.54 mmol, 199 equiv). The mixture is then heated for 8 d at 60 °C. The solvent was removed in vacuo and the residue dissolved in 0.4 mL of CD_3_CN and filtered. **12**: ^1^H NMR (400 MHz, CD_3_CN): *δ* [ppm]=7.36 (s, residual proton signal, H‐2/5), 7.10 (s, 2 H, H‐7/9), 6.87 (s, 2 H, H‐3/4), 4.38 (t, ^3^ *J*_HH_=7.0 Hz, 4 H, NCH_2_), 3.66 (s, 6 H, NCH_3_), 2.30 (s, 6 H, CCH_3_), 1.98 (tq, 4 H, ^3^ *J*_HH_=7.4 Hz, ^3^ *J*_HH_=7.0 Hz, CH_2_), 1.01 (t, 6 H, ^3^ *J*_HH_=7.4 Hz, CH_3_). ^13^C{^1^H} NMR (100 MHz, CD_3_CN): *δ* [ppm]=143.2 (C‐10), 132.8 (C‐6a/9b), 127.3 (CCH_3_), 121.2 (C‐2a/4a), 116.2 (C‐3/4), 116.0 (C‐6b/9a), 113.1 (C‐7/9), 106.2 (C‐2), 51.6 (NCH_2_), 34.0 (NCH_3_), 23.6 (CH_2_), 10.9 (CH_3_), 9.1 (CCH_3_). The signal for C‐2/5 could not be detected. **13**: ^1^H NMR (400 MHz, CD_3_CN): *δ* [ppm]=7.34 (s, residual proton signal, H‐2), 7.24 (s, residual proton signal, H‐5), 7.11 (d, 1 H, ^3^ *J*_HH_=4.3 Hz, H‐9), 6.84 (d, 1 H, ^3^ *J*_HH_=4.3 Hz, H‐8), 6.82 (s, 1 H, H‐3 or H‐4), 6.80 (s, 1 H, H‐3 or H‐4), 4.41–4.37 (m, 2 H, NCH_2_^b^), 3.50 (s, 6 H, NCH_3_), 3.18–3.14 (m, 2 H, NCH_2_^a^), 2.33 (s, 6 H, CCH_3_), 2.02–1.96 (m, 2 H, CH_2_^b^), 1.61–1.51 (m, 2 H, CH_2_^a^), 1.04 (t, 3 H, ^3^ *J*_HH_=7.3 Hz, CH_3_^b^), 0.71 (t, 3 H, ^3^ *J*_HH_=7.3 Hz, CH_3_^a^). The ^13^C signals could not be assigned unambiguously due to the low intensity. Both: MS (ESI^+^, CH_3_CN): *m*/*z=*215.1 [M]^2+^, 575.2 [M+PF_6_]^+^. The deuteration degree of 97 % was determined from the ESI‐spectrum.

***Modified synthesis of 1,2,3,4,5‐pentamethylruthenocene (15)***:[^29^, ^53^] To a suspension of LiCp (15.0 mg, 208 μmol, 1.05 equiv) in 2 mL of acetonitrile a solution of [Ru(CH_3_CN)_3_Cp*](PF_6_) (100 mg, 198 μmol, 1 equiv) in 1 mL of acetonitrile was added under stirring. After 10 min, the solvent of the formed solution was removed in vacuo, and the residue extracted three times with 3 mL pentane. The combined extracts were filtered over neutral aluminum oxide and concentrated to dryness in vacuo to obtain the product **15** as a colorless, air‐stable, crystalline solid (56.4 mg, 187 μmol, 95 %). ^1^H NMR (400 MHz, [D_6_]acetone): *δ* [ppm]=4.14 (s, 5 H, Cp), 1.93 (s, 15 H, Cp*). ^1^H NMR (400 MHz, CD_3_CN): *δ* [ppm]=4.15 (s, 5 H, Cp), 1.93 (s, 15 H, Cp*). CHN: calcd C 59.78, H 6.69 found C 59.82, H 6.51. NMR data match with those from literature.^[29]^

***Synthesis of 1‐(1,3,4,5‐tetramethylimidazolium)‐1’,2’,3’,4’,5’‐pentamethylruthenocene hexafluorophosphate (16)***: To a suspension of **2** (29.9 mg, 159 μmol, 1 equiv) in 0.75 mL of acetonitrile a solution of [Ru(CH_3_CN)_3_Cp*](PF_6_) (80.0 mg, 159 μmol, 1 equiv) in 0.75 mL of acetonitrile was added under stirring. The suspension turned yellow and the solid dissolved after 30 min. The solvent was removed in vacuo and the residue extracted 3 times with 0.5 mL dichloromethane. The product was precipitated from the combined extract by addition of 5 mL of pentane, filtered off and dried in vacuo to obtain **16** as an off‐white, air‐stable crystalline solid (86.2 mg, 151 μmol, 95 %). Crystals suitable for X‐ray analysis were obtained by diffusion of pentane into a solution of **16** in dichloromethane. ^1^H NMR (400 MHz, CD_3_CN): *δ* [ppm]=4.77–4.76 (m, 2 H, 2/5‐H), 4.55–4.54 (m, 2 H, 3/4‐H), 3.74 (s, 6 H, NCH_3_), 2.22 (s, 6 H, CCH_3_), 1.86 (s, 15 H, Cp*). ^13^C{^1^H} NMR (100 MHz, CD_3_CN): *δ* [ppm]=143.9 (C‐6), 127.3 (CCH_3_), 88.2 (Cp*‐CCH_3_), 77.5 (C‐3/4), 74.8 (C‐2/5), 71.6 (C‐1), 34.4 (NCH_3_), 11.8 (Cp*‐CCH_3_), 9.1 (CCH_3_). ^19^F{^1^H} NMR (376 MHz, CD_3_CN): *δ* [ppm]=−72.9 (d, ^1^ *J*_PF_=707 Hz, PF_6_). ^31^P{^1^H} NMR (162 MHz, CD_3_CN): *δ* [ppm]=−144.6 (spt, ^1^ *J*_PF_=707 Hz, PF_6_). ^1^H NMR (400 MHz, CD_2_Cl_2_): *δ* [ppm]=4.65–4.64 (m, 2 H, 2/5‐H), 4.54–4.53 (m, 2 H, 3/4‐H), 3.78 (s, 6 H, NCH_3_), 2.26 (s, 6 H, CCH_3_), 1.87 (s, 15 H, Cp*). CHN: calcd C 46.40, H 5.49, N 4.92 found C 46.41, H 5.37, N 5.14. MS (ESI^+^, CH_3_CN): *m*/*z=*425.2 [M]^+^. UV/VIS (CH_3_CN): *λ*_1_=280 nm (*ϵ*=3.5×10^4^ L mol^−1^⋅cm^−1^), *λ*_2_=332 nm (*ϵ*=1.7×10^4^ L mol^−1^⋅cm^−1^). IR (KBr): *ṽ* [cm^−1^]=3137 (w), 2966 (w), 2901 (w), 1646 (m), 1552 (m), 1511 (m), 1452 (m), 1384 (m), 1262 (w), 1046 (w), 1028 (w), 850 (vs., PF_6_^−^), 827 (vs., PF_6_^−^), 710 (w), 558 (s, PF_6_^−^), 444 (w). Mp.: 193 °C.

***Synthesis of 1,3‐bis(1,3,4,5‐tetramethylimidazolium)‐1’,2’,3’,4’,5’‐pentamethylruthenocene hexafluorophosphate (17)***: To a solution of [Ru(CH_3_CN)_3_Cp*](PF_6_) (50.0 mg, 99.1 μmol, 1 equiv) in 1.5 mL of acetonitrile a solution of **5** (45.2 mg, 99.1 μmol, 1 equiv) in 1.5 mL of acetonitrile was added under stirring. The solution turned brownish and after 15 min the solvent was removed in vacuo. The residue was extracted three times with 0.4 mL of dichloromethane and the solvent removed in vacuo to obtain the ruthenocene **17** as an off‐white, air‐stable crystalline solid (75.5 mg, 90.1 μmol, 91 %). Crystals suitable for X‐ray analysis were obtained by slow evaporation of acetonitrile from a solution of **17**. ^1^H NMR (400 MHz, CD_3_CN): *δ* [ppm]=5.12 (d, ^3^ *J*_HH_=1.3 Hz, 2 H, 4/5‐H), 5.03 (t, ^3^ *J*_HH_=1.3 Hz, 1 H, 2‐H), 3.80 (s, 12 H, NCH_3_), 2.26 (s, 12 H, CCH_3_), 1.76 (s, 15 H, Cp*). ^13^C{^1^H} NMR (100 MHz, CD_3_CN): *δ* [ppm]=140.1 (C‐6/7), 128.5 (CCH_3_), 90.5 (Cp*‐CCH_3_), 77.6 (C‐4/5), 76.9 (C‐2), 75.6 (C‐1/3), 34.7 (NCH_3_), 11.1 (Cp*‐CCH_3_), 9.2 (CCH_3_). ^19^F{^1^H} NMR (376 MHz, CD_3_CN): *δ* [ppm]=−72.9 (d, ^1^ *J*_PF_=707 Hz, PF_6_). ^31^P{^1^H} NMR (162 MHz, CD_3_CN): *δ* [ppm]=−144.6 (spt, ^1^ *J*_PF_=707 Hz, PF_6_). ^1^H NMR (400 MHz, CD_2_Cl_2_): *δ* [ppm]=5.36 (t, ^3^ *J*_HH_=1.3 Hz, 1 H, 2‐H), 5.12 (d, ^3^ *J*_HH_=1.3 Hz, 2 H, 4/5‐H), 3.89 (s, 12 H, NCH_3_), 2.30 (s, 12 H, CCH_3_), 1.79 (s, 15 H, Cp*). CHN: calcd C 41.58, H 5.05, N 6.69 found C 41.37, H 4.93, N 6.66. MS (ESI^+^, CH_3_CN): *m*/*z=*274.1 [M]^2+^. MS (ESI^−^, CH_3_CN): *m*/*z=*144.9 [PF_6_]^−^. UV/VIS (CH_3_CN): *λ*=280 nm (*ϵ*=2.2×10^4^ L mol^−1^⋅cm^−1^). IR (KBr): *ṽ* [cm^−1^]=2964 (w), 2918 (w), 1650 (m), 1520 (m), 1458 (m), 1387 (w), 1299 (w), 1287 (w), 1238 (w), 1128 (w), 1070 (w), 840 (vs., PF_6_^−^), 709 (w), 557 (s, PF_6_^−^), 501 (w), 451 (w). Mp.: 264 °C (dec.).

***Synthesis of 18***: To a solution of [Ru(CH_3_CN)_3_Cp*](PF_6_) (30.0 mg, 59.5 μmol, 1 equiv) in 0.5 mL dichloromethane a suspension of **9** (26.8 mg, 59.5 μmol, 1 equiv) in 0.5 mL dichloromethane was added under stirring. The solid dissolved and a yellow crystalline solid formed. After 30 min the solvent was removed in vacuo and the solid was suspended in 0.5 mL dichloromethane. After 30 min the solvent is again removed in vacuo and the solid was washed two times with 0.35 mL of dichloromethane. The residue was dried in vacuo to obtain the product **18** as a yellow, crystalline solid (40.5 mg, 48.7 μmol, 82 %). Crystals suitable for X‐ray structure analysis were obtained by overlaying a solution of **18** in acetonitrile with diethyl ether. ^1^H NMR (400 MHz, CD_3_CN): *δ* [ppm]=7.71 (s, 2 H, H‐2/5), 7.18 (s, 2 H, H‐3/4), 5.78 (d, 2 H, ^3^ *J*_HH_=2.9 Hz, H‐7/9), 5.38 (t, 1 H, ^3^ *J*_HH_=2.9 Hz, H‐8), 4.44–4.38 (m, 2 H, NCH_2_), 4.30–4.24 (m, 2 H, NCH_2_), 2.01 (ps sxt, 4 H, ^3^ *J*_HH_=7.3 Hz, CH_2_), 1.74 (s, 15 H, Cp*), 1.05 (ps t, 6 H, ^3^ *J*_HH_=7.4 Hz, CH_3_). ^13^C{^1^H} NMR (126 MHz, CD_3_CN): *δ* [ppm]=135.7 (C‐6a/9b, signal from HMBC), 122.8 (C‐2a/4a), 118.9 (C‐2/5), 116.8 (C‐3/4), 92.4 (Cp*‐CCH_3_), 84.3 (C‐8), 73.8 (C‐7/9), 68.0 (C‐6b/9a), 52.6 (NCH_2_), 23.9 (CH_2_), 11.5 (Cp*‐CCH_3_), 10.8 (CH_3_). ^19^F{^1^H} NMR (376 MHz, CD_3_CN): *δ* [ppm]=−72.9 (d, ^1^ *J*_PF_=707 Hz, PF_6_). ^31^P{^1^H} NMR (162 MHz, CD_3_CN): *δ* [ppm]=−144.6 (spt, ^1^ *J*_PF_=707 Hz, PF_6_). CHN: calcd C 41.88, H 4.36, N 6.74 found C 41.73, H 4.12, N 6.81. MS (ESI^+^, CH_2_Cl_2_): *m*/*z=*270.8 [M]^2+^, 305.1 [M‐RuCp*]^+^, 687.1 [M+PF_6_]^+^. Mp.: 230 °C (dec.).

***Formation of 19 and 20***: To a suspension of LiCp* (900 mg, 6.33 mmol, 2.00 equiv) in 30 mL tetrahydrofuran [(C_6_H_6_)RuCl_2_]_2_ (1.58 g, 3.16 mmol, 1 equiv) was added as solid with 30 mL tetrahydrofuran and the mixture was stirred for 2 h. The solvent was removed in vacuo and 200 mL water were added to the residue. The solution was filtered and a concentrated solution of KPF_6_ (1.17 g, 6.35 mmol, 2.01 equiv) in water was added under stirring. The formed off‐white precipitate was filtered off and washed with 10 mL diethyl ether. The residue was dissolved in acetone, filtered over neutral aluminum oxide and the filtrate concentrated to dryness. After dissolving the residue in dichloromethane and addition of diethyl ether, the formed precipitate was collected, washed with diethyl ether and dried in vacuo to obtain **19** (360 mg, 784 μmol, 12 %) as an off‐white air stable solid. ^1^H NMR (400 MHz, [D_6_]acetone): *δ* [ppm]=6.03 (s, 6 H, C_6_H_6_), 2.07 (s, 15 H, Cp*). ^1^H NMR (400 MHz, CD_3_CN): *δ* [ppm]=5.76 (s, 6 H, C_6_H_6_), 1.97 (s, 15 H, Cp*). ^1^H NMR (400 MHz, CDCl_3_): *δ* [ppm]=5.80 (s, 6 H, C_6_H_6_), 2.03 (s, 15 H, Cp*). CHN: calcd C 41.83, H 4.61 found C 41.75, H 4.45. NMR data in acetone matches with that in the literature.^[34]^

The mother liquor of the precipitation from dichloromethane with diethyl ether was concentrated to dryness and the residue recrystallized from dichloromethane/diethyl ether and subsequently from acetone/diethyl ether to obtain **20** (262 mg) as an off‐white air stable solid which contains residual **19** (about 3 %). Crystals suitable for X‐ray structure analysis were obtained by crystallization from acetone/diethyl ether. ^1^H NMR (400 MHz, CDCl_3_): *δ* [ppm]=5.69–5.67 (m, 2 H, 3‐H), 5.61–5.59 (m, 3 H, 2‐H and 4‐H), 1.98 (s, 15 H, Cp*), 1.76 (s, 6 H, 7‐H), 1.72 (s, 6 H, 6‐H), 1.22 (s, 3 H, 5‐H). ^13^C NMR (100 MHz, CDCl_3_): *δ* [ppm]=139.8 (CCH_3_‐6), 136.8 (CCH_3_‐7) 113.7 (C‐1), 95.9 (Cp*‐CCH_3_), 86.6 (C‐4), 86.5 (C‐3), 83.5 (C‐2), 58.0 (CCH_3_‐5), 18.1 (CH_3_‐5), 11.2 (CH_3_‐7), 10.9 (Cp*‐CCH_3_), 10.7 (CCH_3_‐6). ^19^F{^1^H} NMR (376 MHz, CDCl_3_): *δ* [ppm]=−72.9 (d, ^1^ *J*_PF_=713 Hz, PF_6_). ^31^P{^1^H} NMR (162 MHz, CDCl_3_): *δ* [ppm]=−144.6 (spt, ^1^ *J*_PF_=713 Hz, PF_6_). ^1^H NMR (400 MHz, [D_6_]acetone): *δ* [ppm]=5.90–5.85 (m, 5 H, 2‐H, 3‐H, 4‐H), 2.06 (s, 15 H, Cp*), 1.82 (s, 6 H, 7‐H), 1.79 (s, 6 H, 6‐H), 1.32 (s, 3 H, 5‐H). MS (ESI^+^, CH_3_CN): *m*/*z=*449.2 [M]^+^.

***Crystal structure analyses***: X‐ray diffraction data were collected on a Bruker APEX II DUO instrument equipped with an IμS microfocus sealed tube and QUAZAR optics for Mo*K*_α_ (*λ*=0.71073 Å) radiation. Corrections for absorption effects were applied using SADABS or TWINABS. All structures were solved by direct methods using the ShelXle[^54^, ^55^, ^56^] software package for structure solution and refinement. In some cases, especially for the PF_6_^−^ ions, the Disordered Structure Refinement (DSR) tool was used for disordered fragments.[^57^, ^58^]In the case of structures **12** and **18** the SQEEZE routine was applied.^[59]^

CCDC 2001145 (**1**), 2001139 (**5**), 2001146 (**6**), 2001141 (**7**), 2001148 (**9**), 2001147 (**12**), 2001143 (**13**), 2001140 (**16**), 2001142 (**17**), 2001149 (**18**) and 2001144 (**20**) contain the supplementary crystallographic data of this paper. These data are provided free of charge by the joint Cambridge Crystallographic Data Centre and Fachinformationszentrum Karlsruhe Access Structures service www.ccdc.cam.ac.uk/structures.

**DFT calculations**: Performed based on density functional theory at the BP86/def2‐TZVP[^60^, ^61^, ^62^, ^63^, ^64^, ^65^] level implemented in Turbomole.[^66^, ^67^, ^68^, ^69^, ^70^, ^71^, ^72^, ^73^, ^74^, ^75^, ^76^] The RI‐approximation[^77^, ^78^, ^79^, ^80^, ^81^, ^82^] was used all over. Geometry optimization and natural bond analysis were performed using the Conductor‐like Screening Model (COSMO)[^83^, ^84^] with the permittivity of acetonitrile (*ϵ*=37.5). Minimum structures were verified at the BP86/def2‐TZVP level by calculating the Hessian matrix and ensuring that it has no imaginary frequency. Despite numerous attempts, for example, including the Grimme dispersion correction,[^85^, ^86^] to optimize the geometry of **12** and **13**, a negative frequency of −9.40 cm^−1^ (**12**) and −17.33 cm^−1^ (**13**) remained. The negative vibrational mode involves the rotation around the *N*‐R bonds. The cartesian coordinates of the geometry optimized structures are available as xyz‐file in the Supporting Information.

## Conflict of interest

The authors declare no conflict of interest.

## Supporting information

As a service to our authors and readers, this journal provides supporting information supplied by the authors. Such materials are peer reviewed and may be re‐organized for online delivery, but are not copy‐edited or typeset. Technical support issues arising from supporting information (other than missing files) should be addressed to the authors.

Supplementary

Supplementary
