# Supplementary material for: Electron‐Deficient Imidazolium Substituted Cp Ligands and their Ru Complexes
Source: Chemistry. 2020 Sep 30;26(69):16291–305. doi: 10.1002/chem.202002801 (PMC7756557; doi:10.1002/chem.202002801)
Supplement: Supplementary file 1 — Supplementary [file CHEM-26-16291-s001.pdf]

Chemistry–A European Journal

Supporting Information

Electron-Deficient Imidazolium Substituted Cp Ligands and their Ru Complexes

Fabio Mazzotta,[a] Georg Zitzer,[b] Bernd Speiser,[b] and Doris Kunz*[a]

Table of contents

1. **Numbering scheme for the NMR signals** S2
2. **NMR spectra** S3
3. **IR spectra** S32
4. **UV/VIS spectra** S34
5. **Cyclic voltammograms of the reductive potential region** S39
6. **Additional X-ray structure analyses** S40
7. **Frontier orbitals of the free ligands** S42
8. **Reference** S44

1. Numbering scheme for the NMR signals

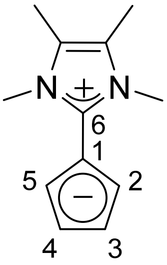

2

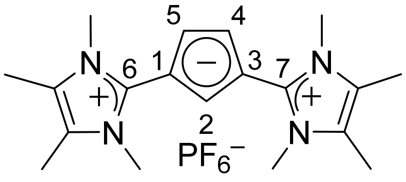

5

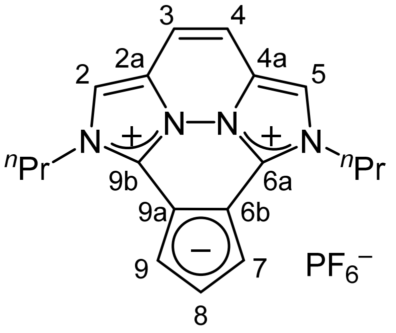

9

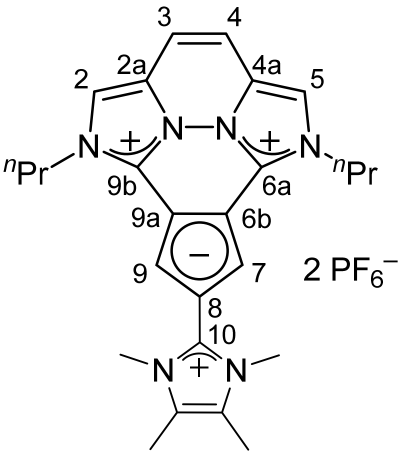

11

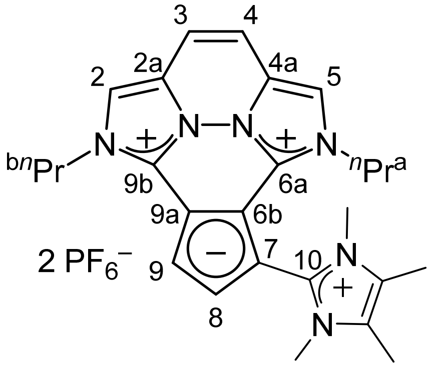

12

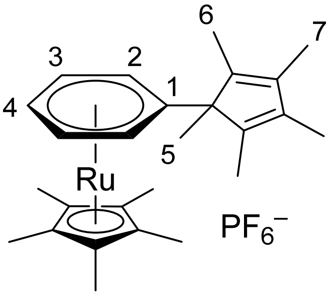

20

Scheme S1. Numbering scheme for the assignment of the ^{1}H and ^{13}C NMR signals.

2. NMR spectra

Spectra of LiCp

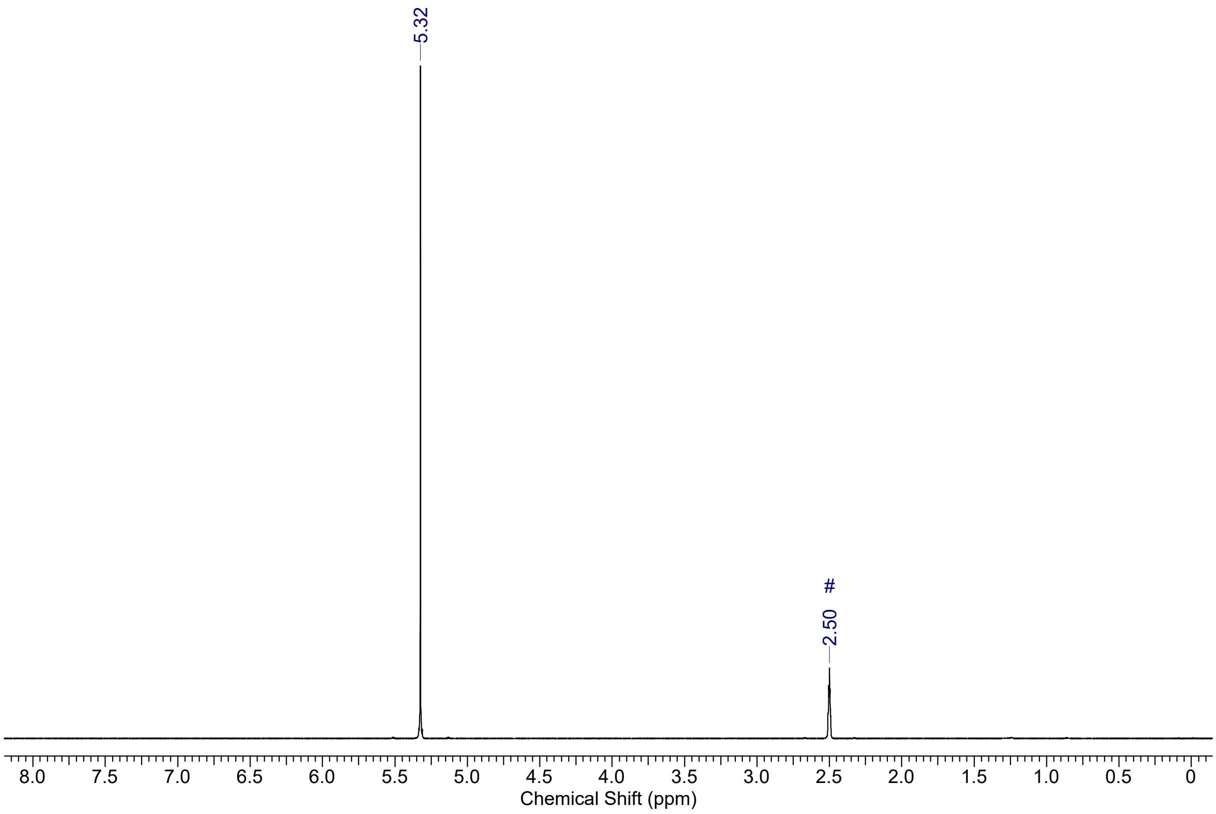

Figure S1. ^{1}H NMR spectrum (400 MHz, DMSO-d_6) of LiCp.

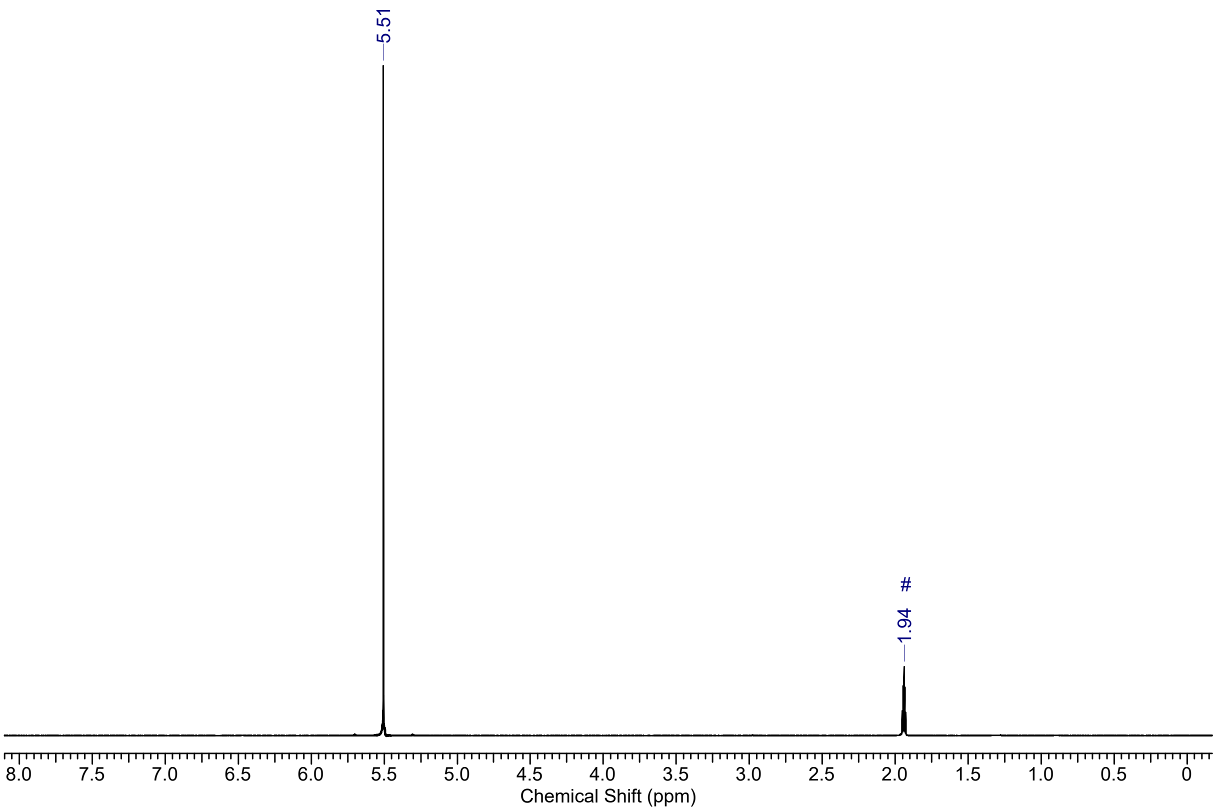

Figure S2. ^{1}H NMR spectrum (400 MHz, CD_3CN #) of LiCp.

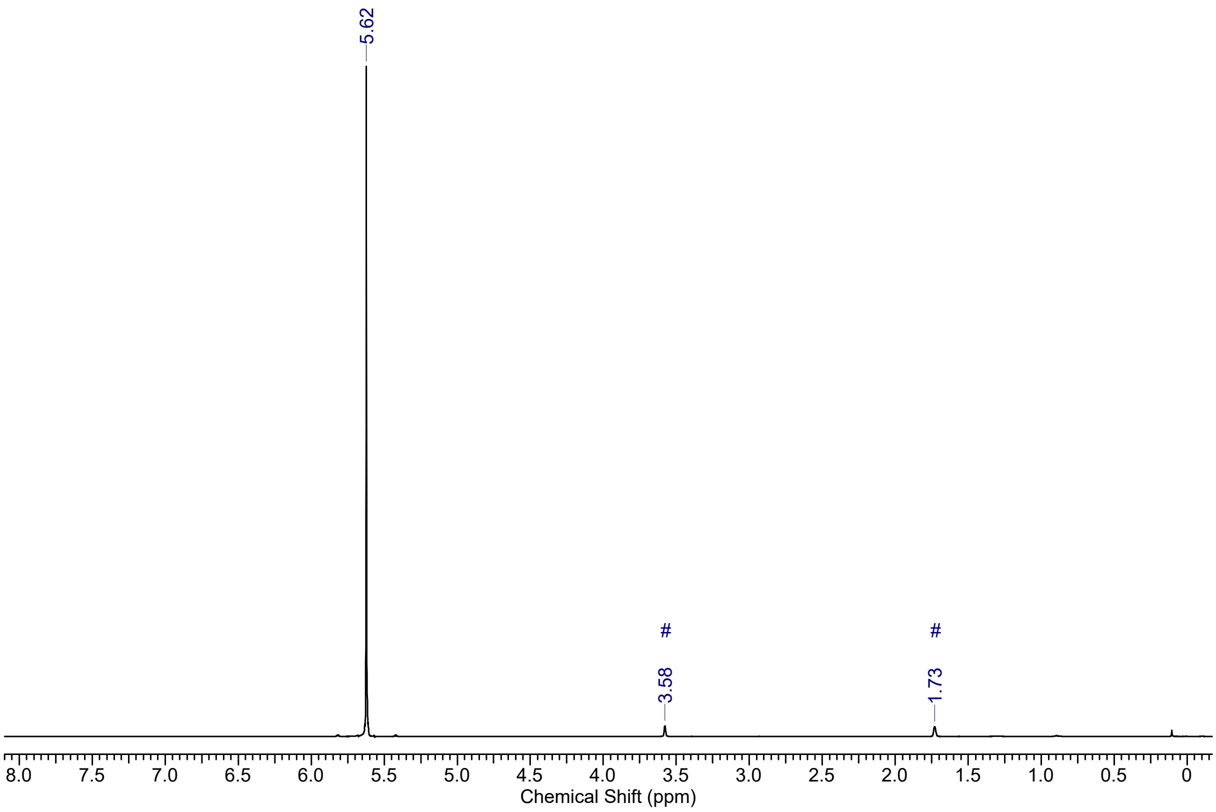

Figure S3. ^{1}H NMR spectrum (400 MHz, THF-d_8 #) of LiCp.

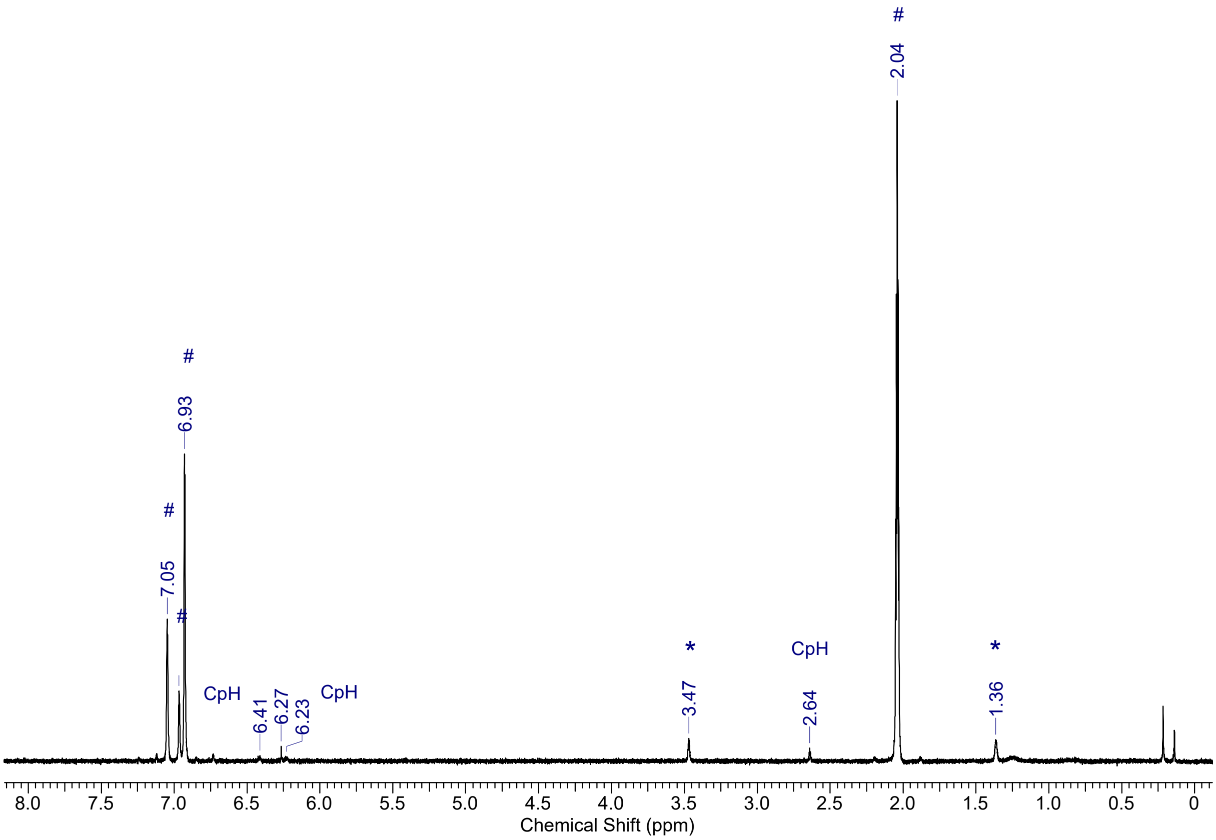

Figure S4. ^{1}H NMR spectrum (400 MHz, toluene-d_8 #: THF-d_8 ∗) of LiCp.

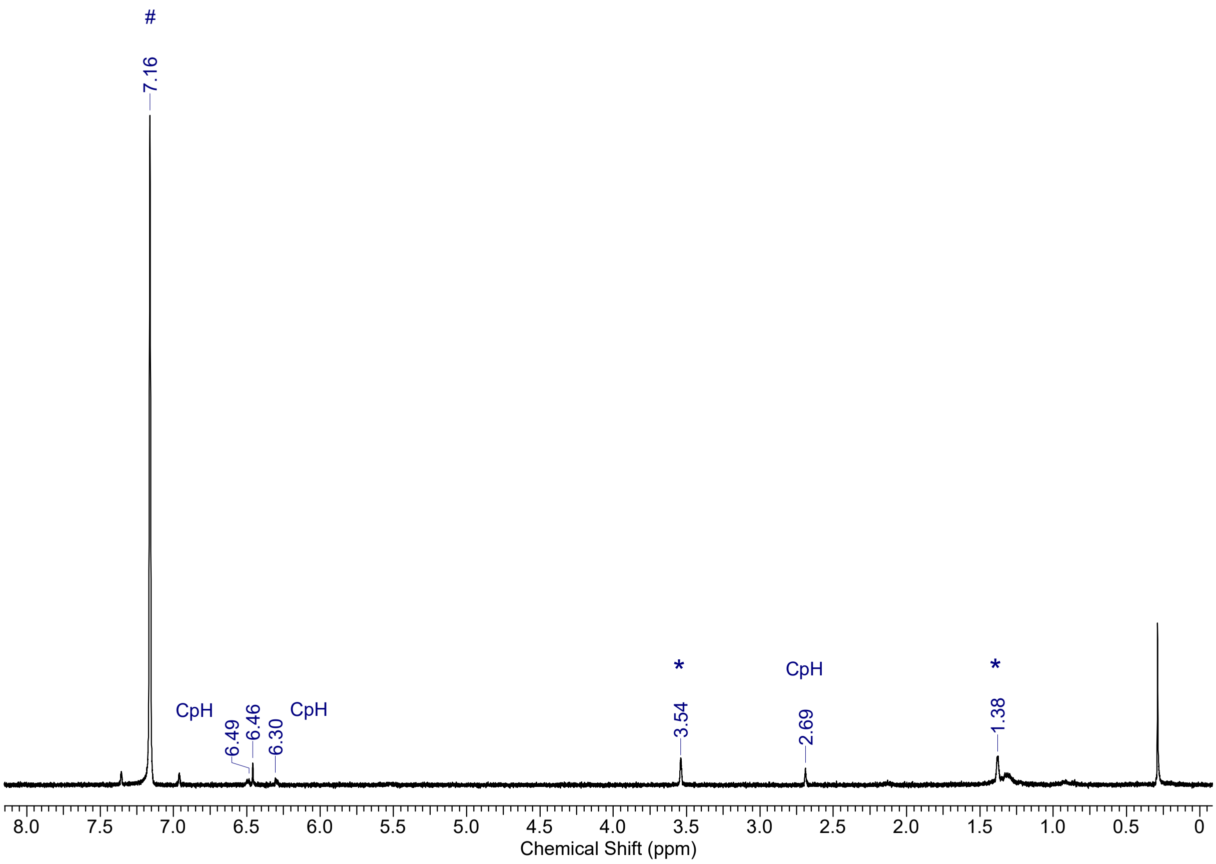

Figure S5. ^{1}H NMR spectrum (400 MH, benzene-d_8 #: THF-d_8 *) of LiCp.

Spectra of 1

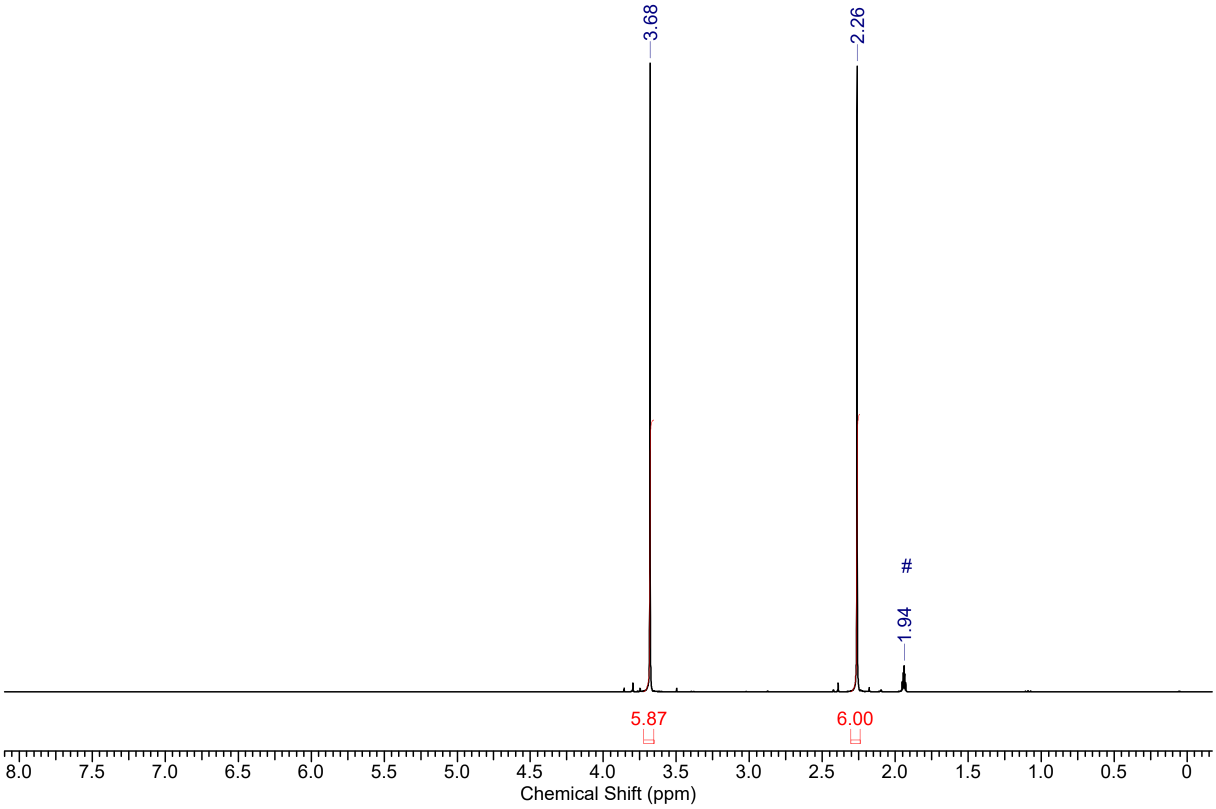

Figure S6. ^{1}H NMR spectrum (400 MHz, CD_3CN #) of **1**.

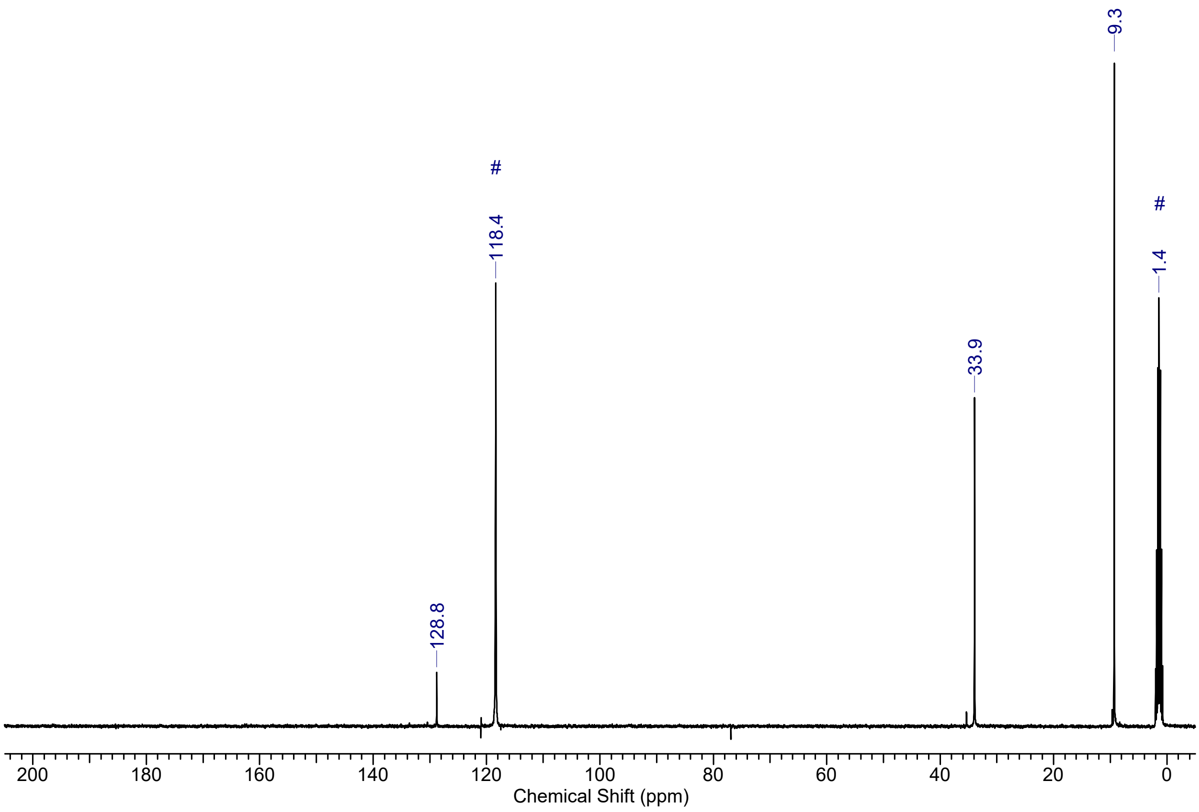

Figure S7. ^{13}C NMR spectrum (100 MHz, CD_3CN #) of **1**.

Spectra of 2

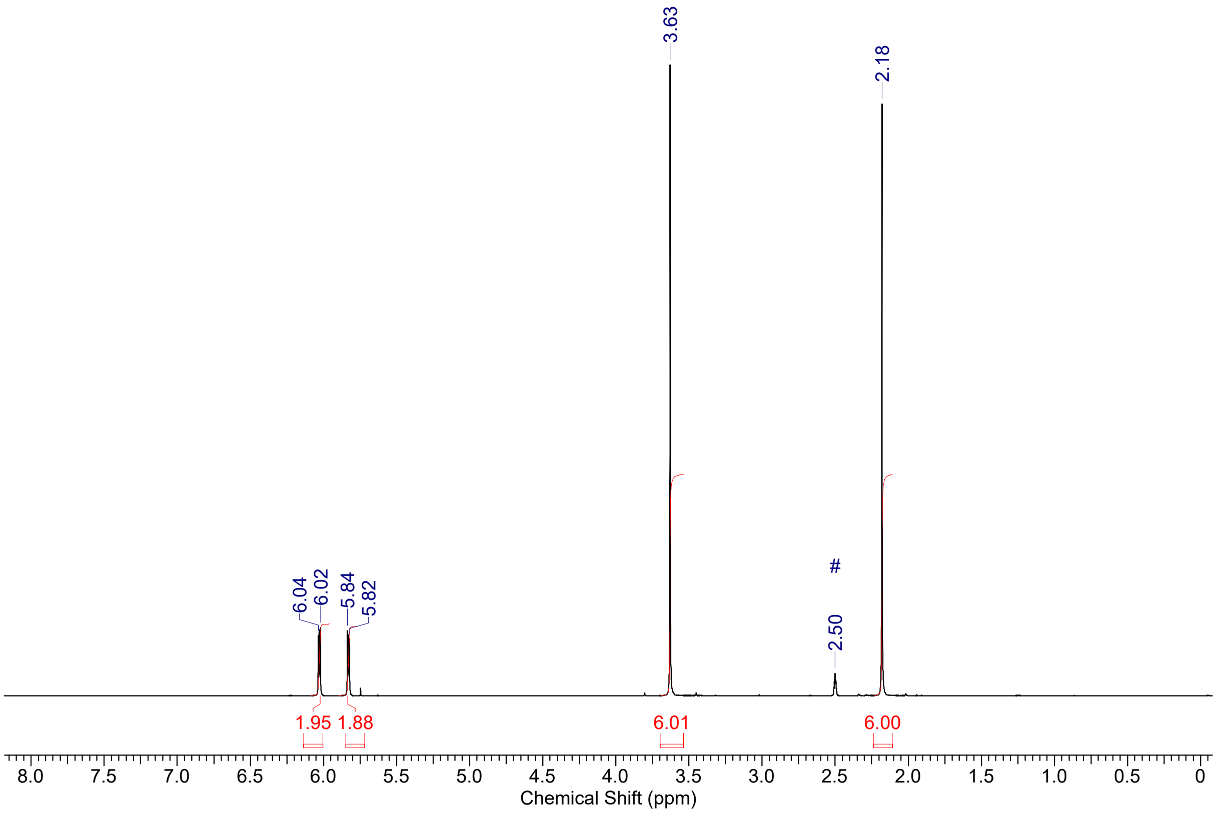

Figure S8. ^{1}H NMR spectrum (400 MHz, DMSO-d_6 #) of **2**.

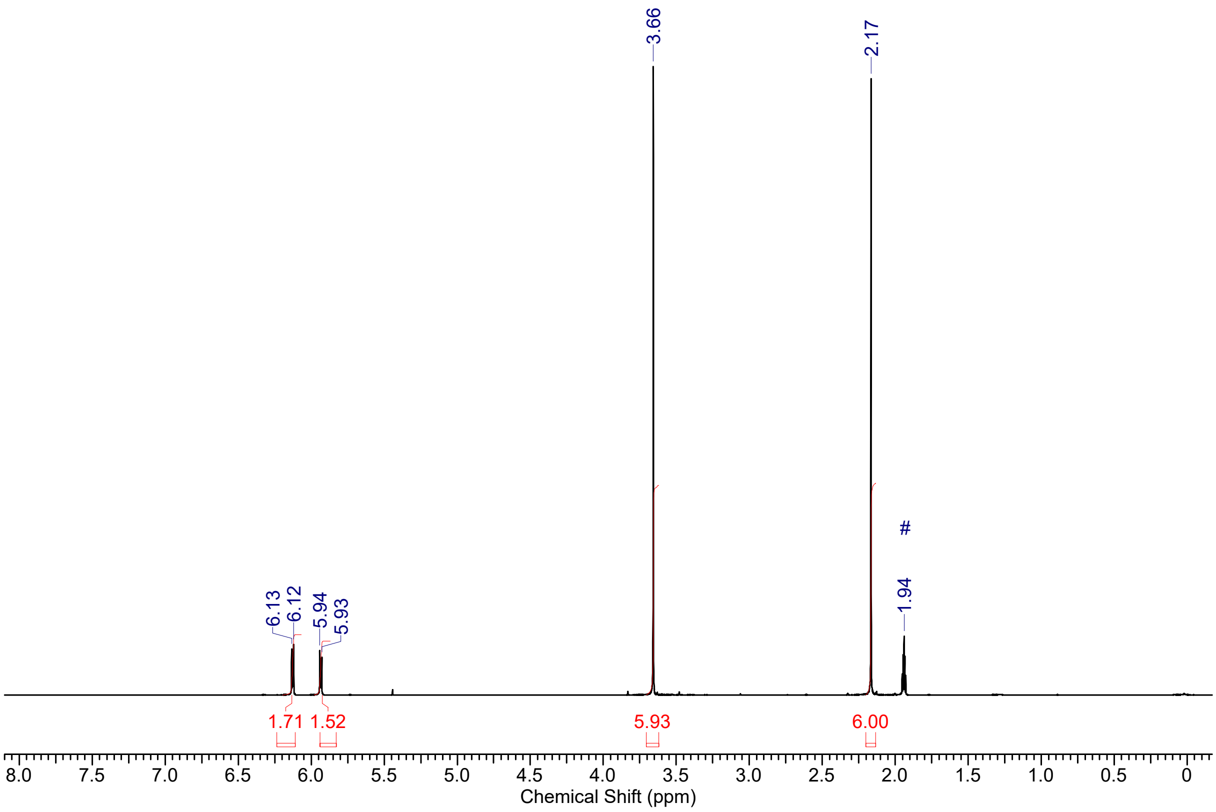

Figure S9. ^{1}H NMR spectrum (400 MHz, CD_3CN #) of **2** (D1 = 1 s).

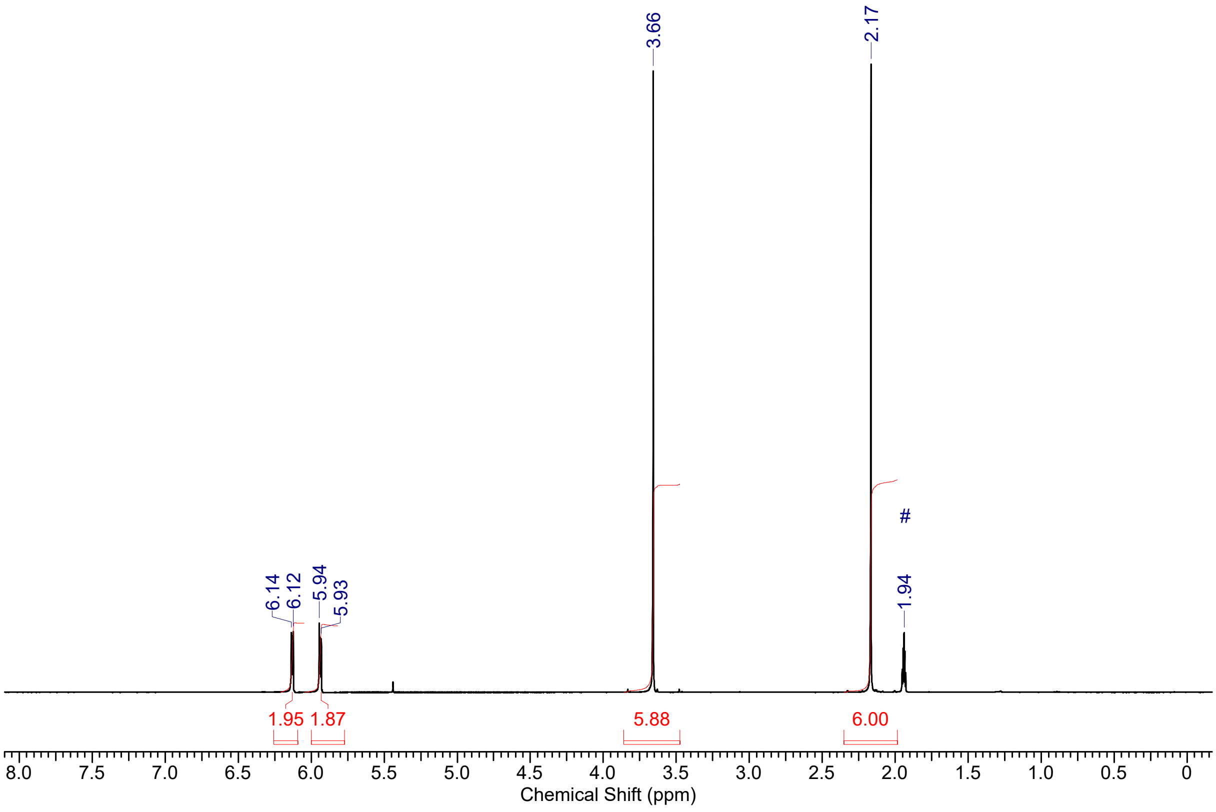

Figure S10. ^{1}H NMR spectrum (400 MHz, CD_3CN #) of **2** (D1 = 60 s).

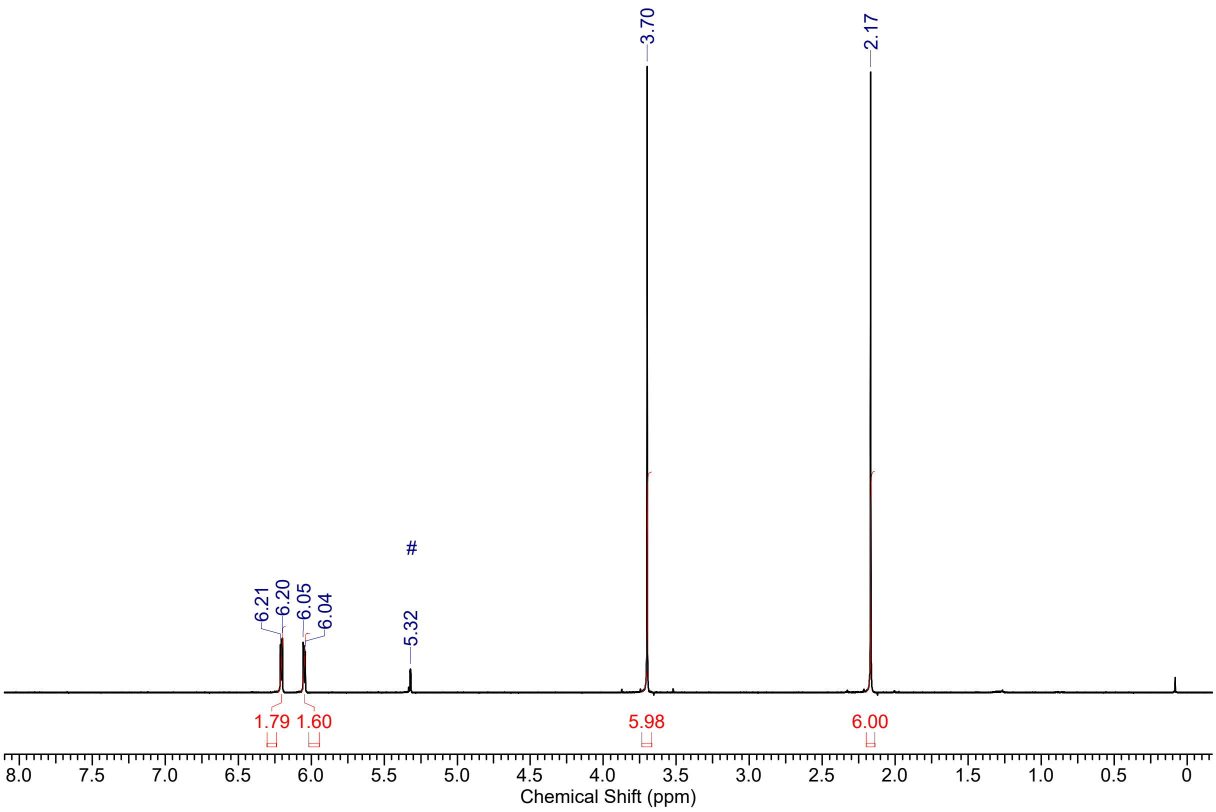

Figure S11. ^{1}H NMR spectrum (400 MHz, CD_2Cl_2 #) of **2**.

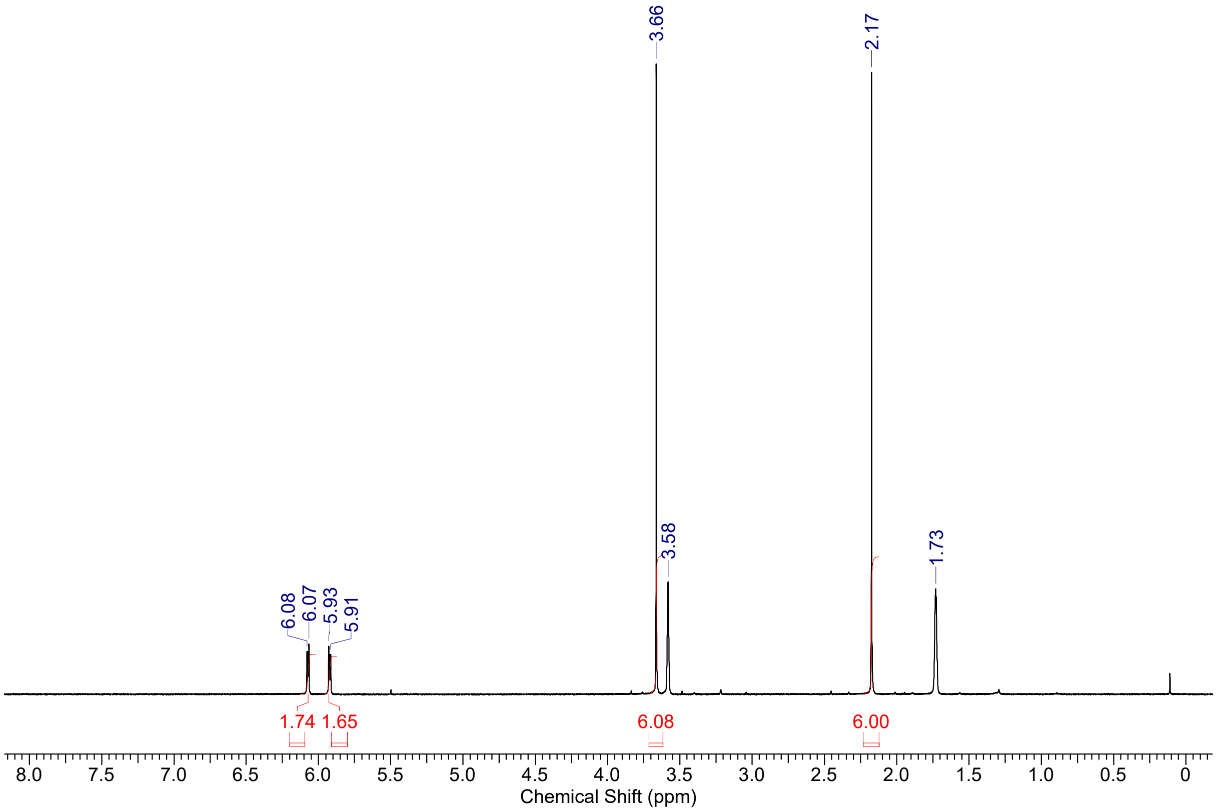

Figure S12. ^{1}H NMR spectrum (400 MHz, THF-d_8 #) of **2**.

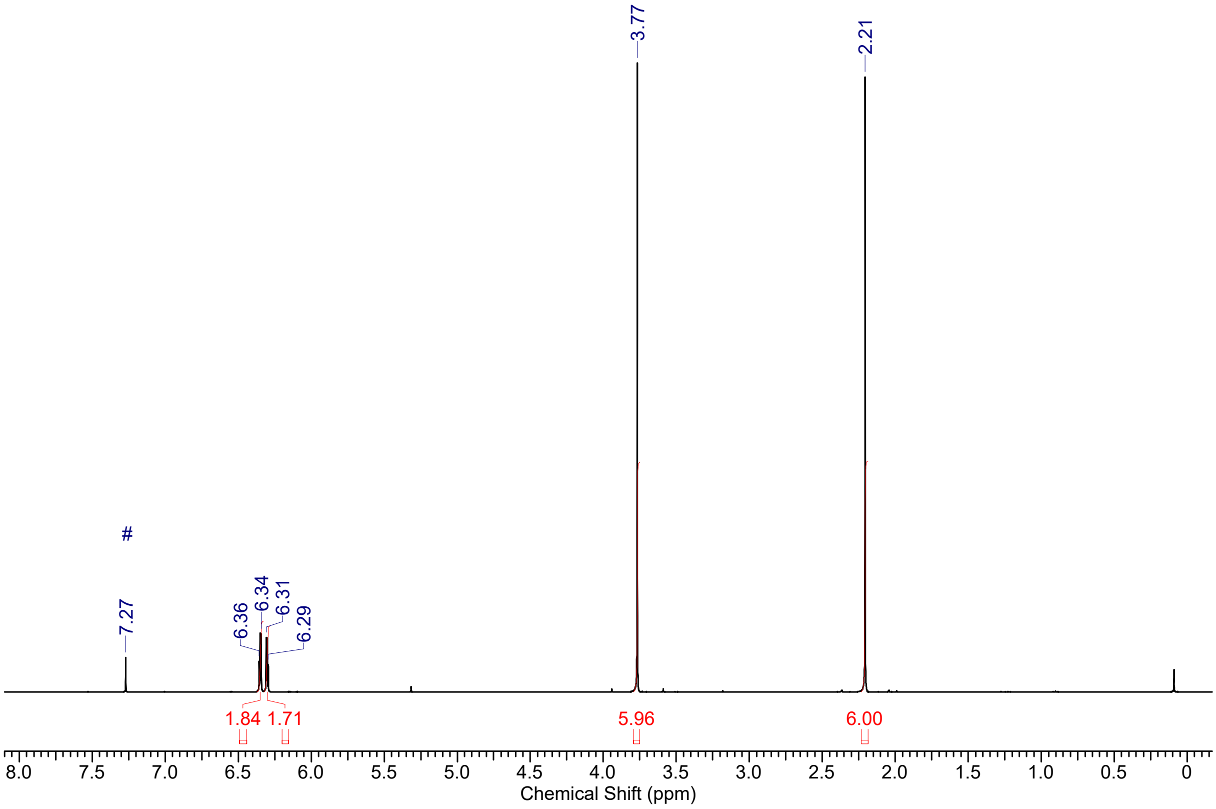

Figure S13: ^{1}H NMR spectrum (400 MHz, $CDCl_3$ #) of **2**.

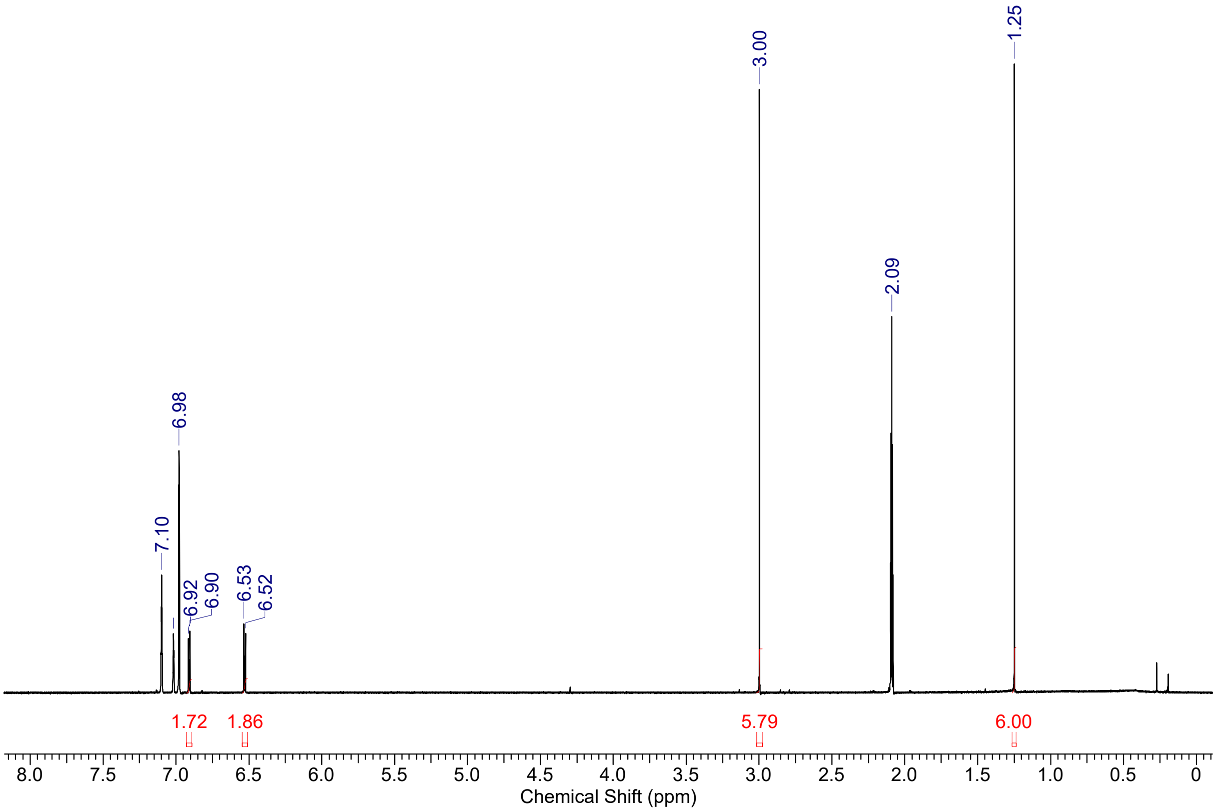

Figure S14. ^{1}H NMR spectrum (500 MHz, toluene-d_8 #) of **2**.

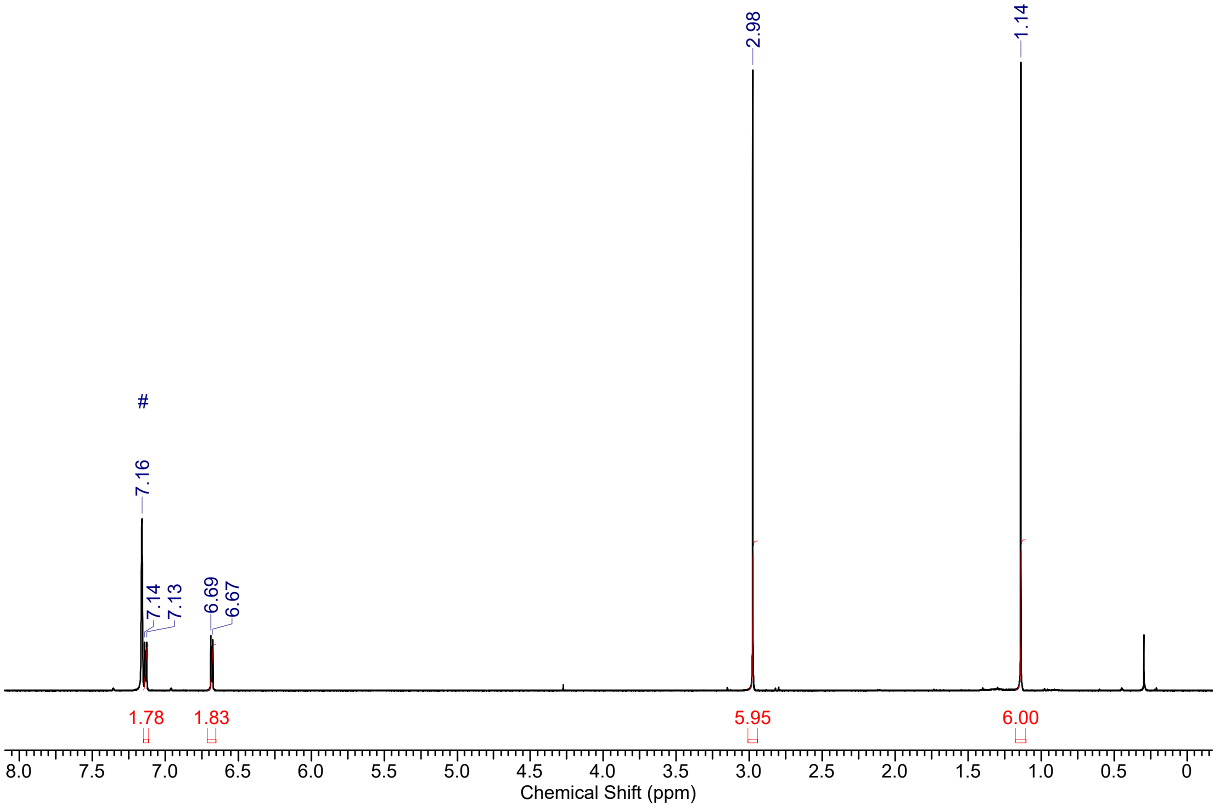

Figure S15. ^{1}H NMR spectrum (400 MHz, C_6D_6 #) of **2**.

Spectra of 3 and 4

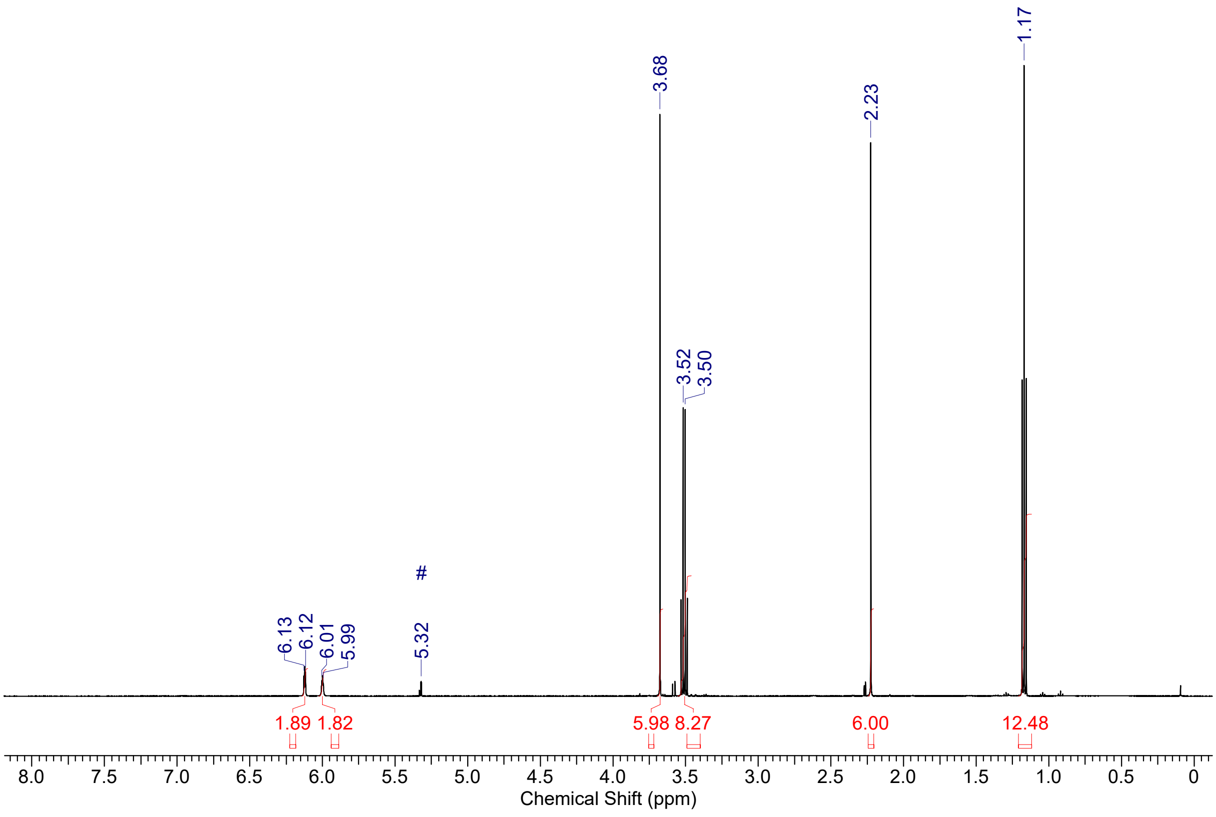

Figure S16. ^{1}H NMR spectrum (500 MHz, CD_2Cl_2 #) of **3**.

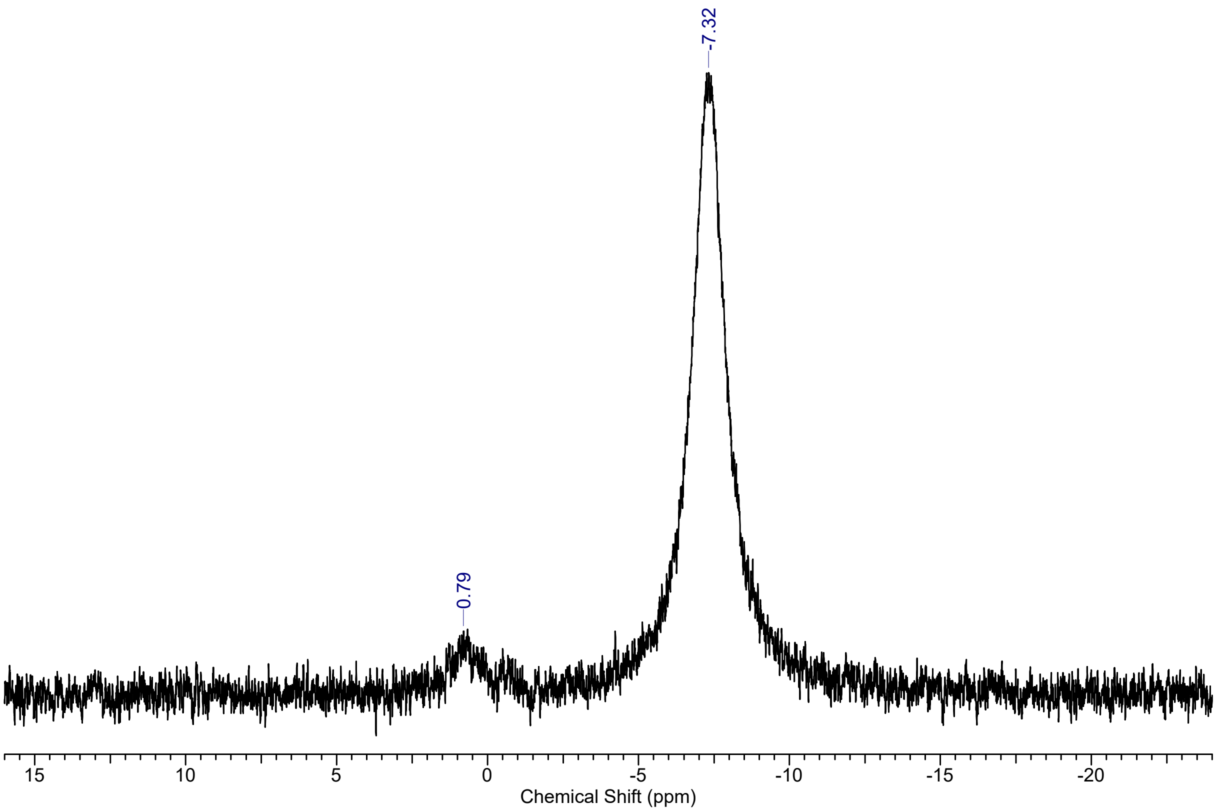

Figure S17. ^{7}Li NMR spectrum (194 MHz, CD_2Cl_2) of **3**.

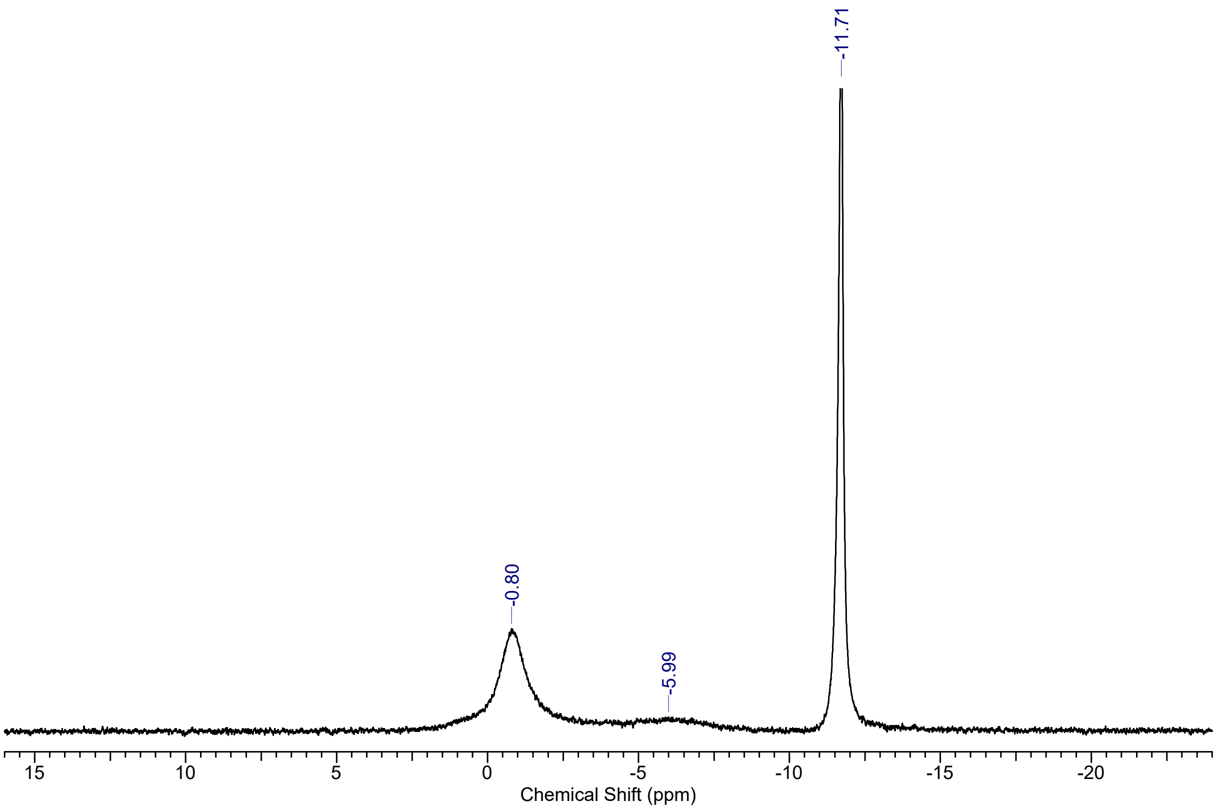

Figure S18. ^{7}Li NMR spectrum (194 MHz, CD_2Cl_2) of **3** and **4** at -80 °C.

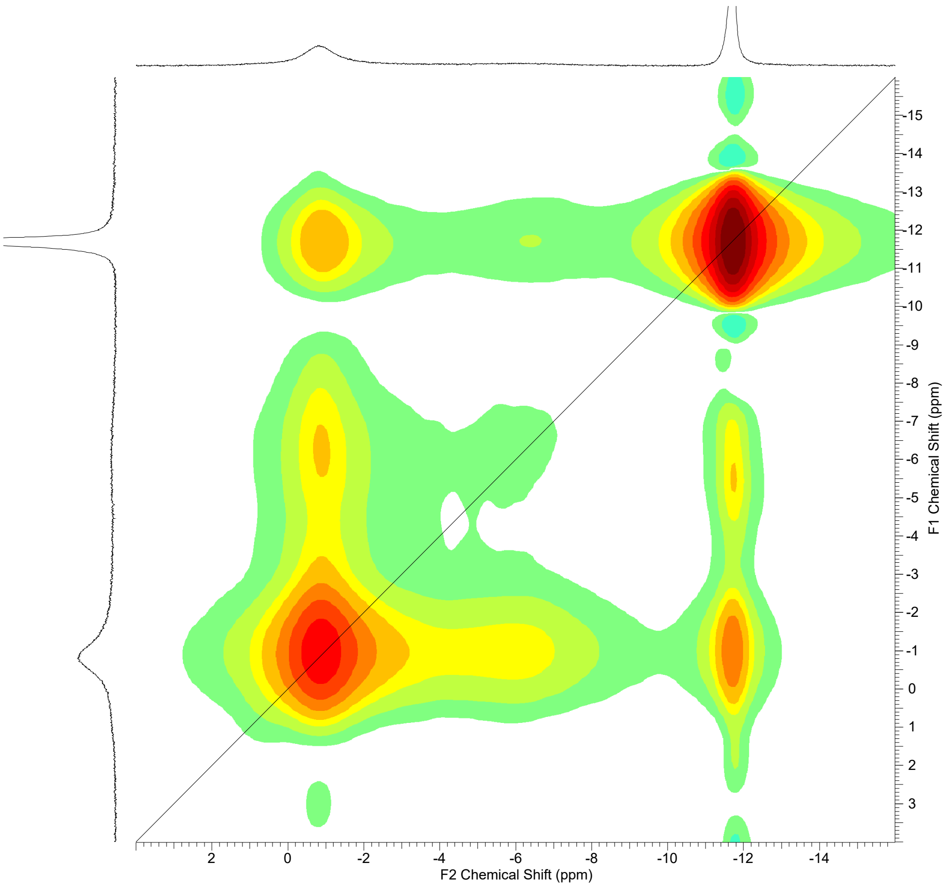

Figure S19. ^{7}Li EXSY NMR spectrum (194 MHz, CD_2Cl_2) of **3** and **4** at -80 °C.

Spectra of 3-NTf_2

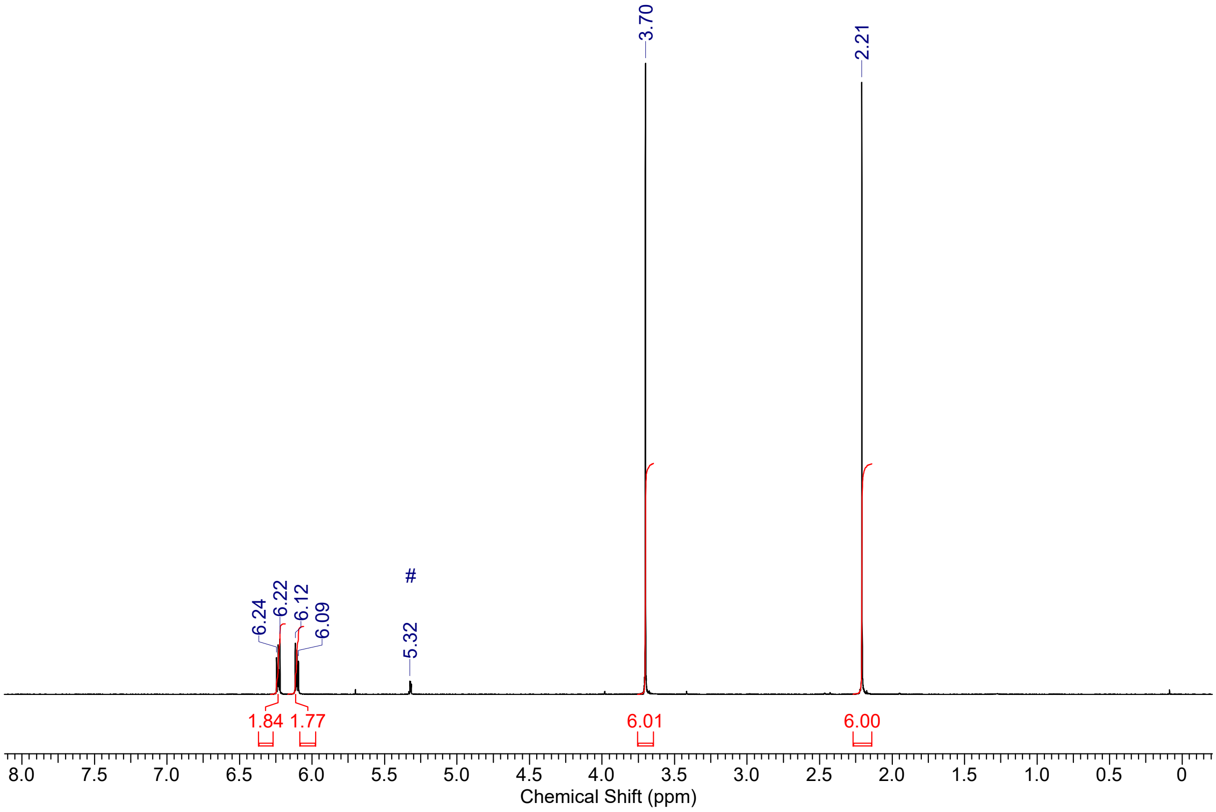

Figure S20. ^{1}H NMR spectrum (250 MHz, CD_2Cl_2 #) of **3-NTf_2**.

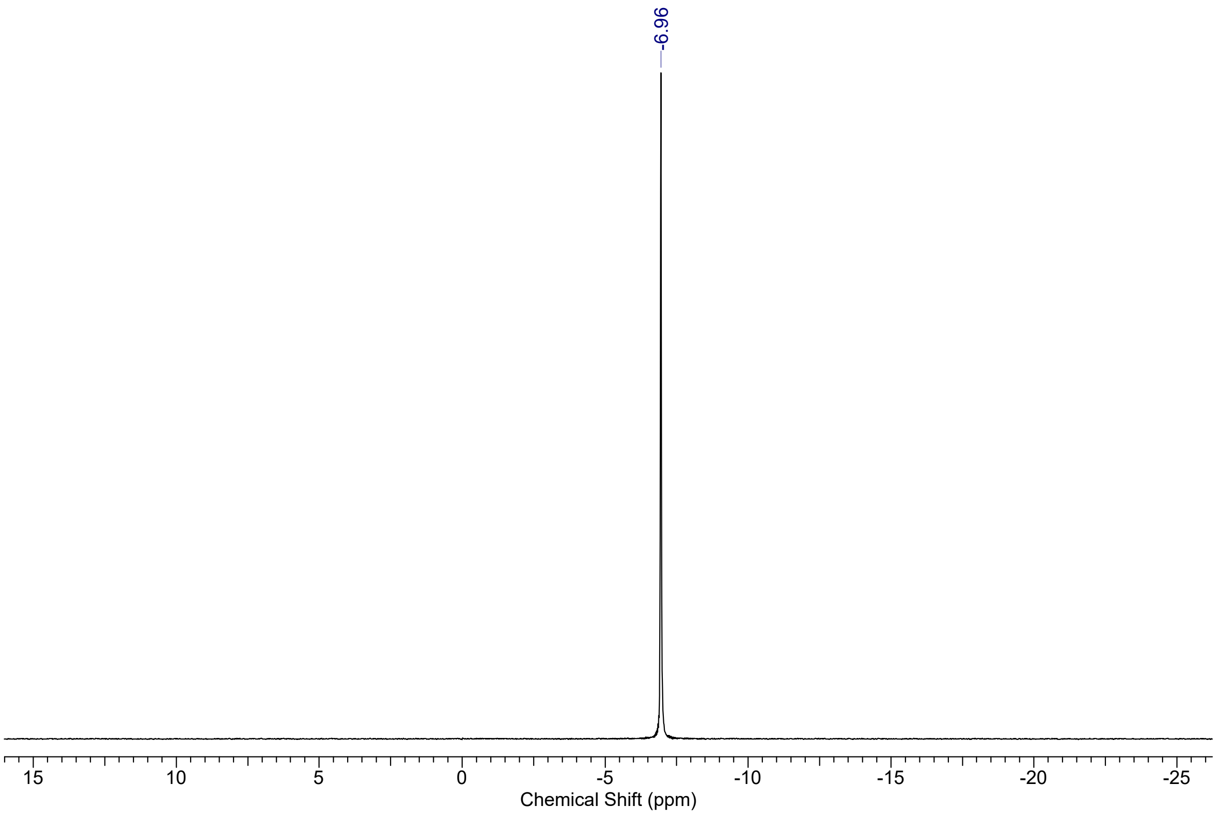

Figure S21. ^{7}Li NMR spectrum (97 MHz, CD_2Cl_2) of **3-NTf$_2$**.

Spectra of 5

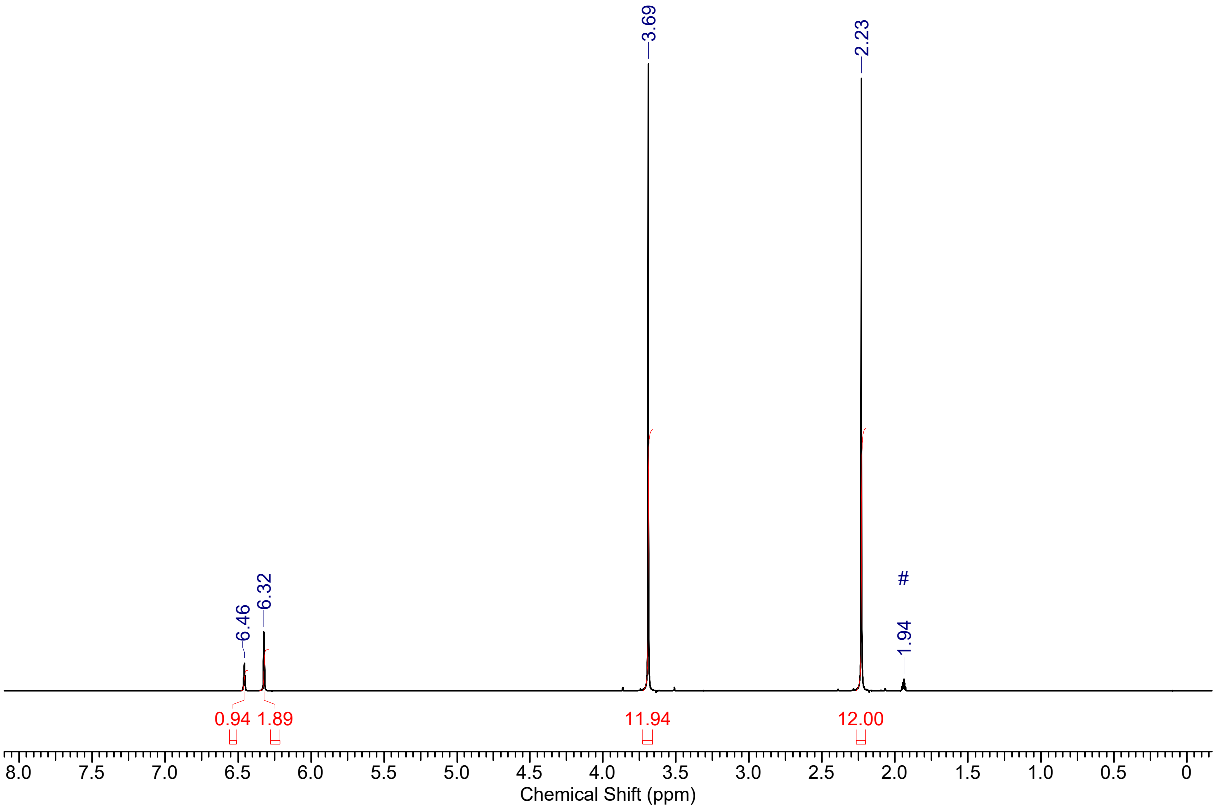

Figure S22. ^{1}H NMR spectrum (400 MHz, CD_3CN #) of **5**.

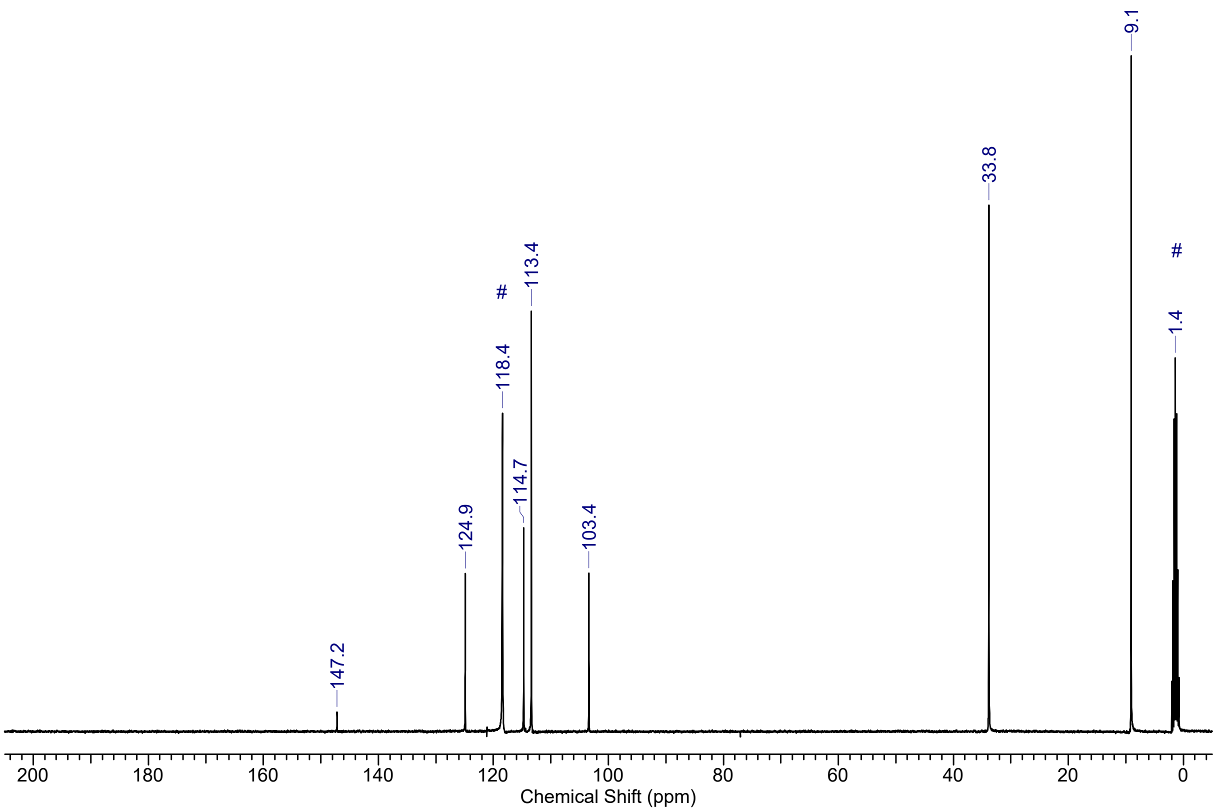

Figure S23. ^{13}C NMR spectrum (100 MHz, CD_3CN #) of **5**.

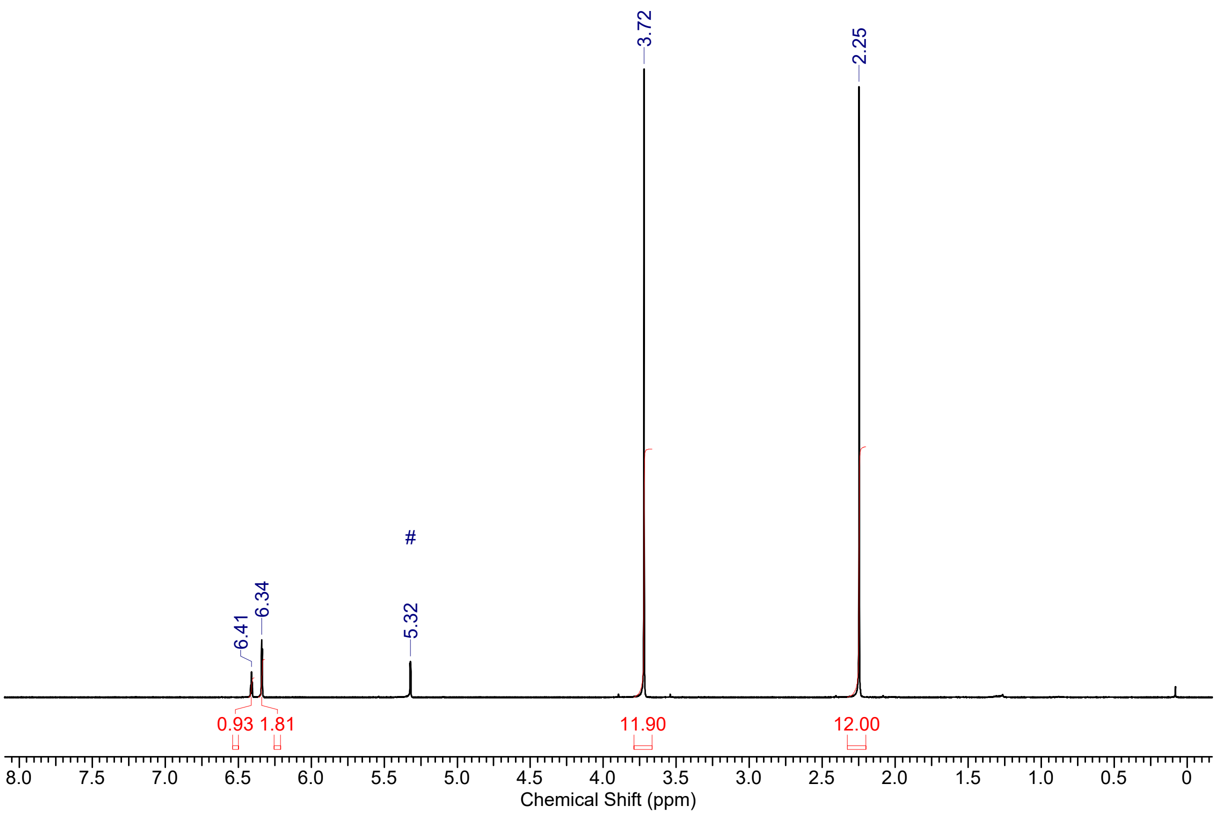

Figure S24. ^{1}H NMR spectrum (400 MHz, CD_2Cl_2 #) of **5**.

Spectra of **5-d3**

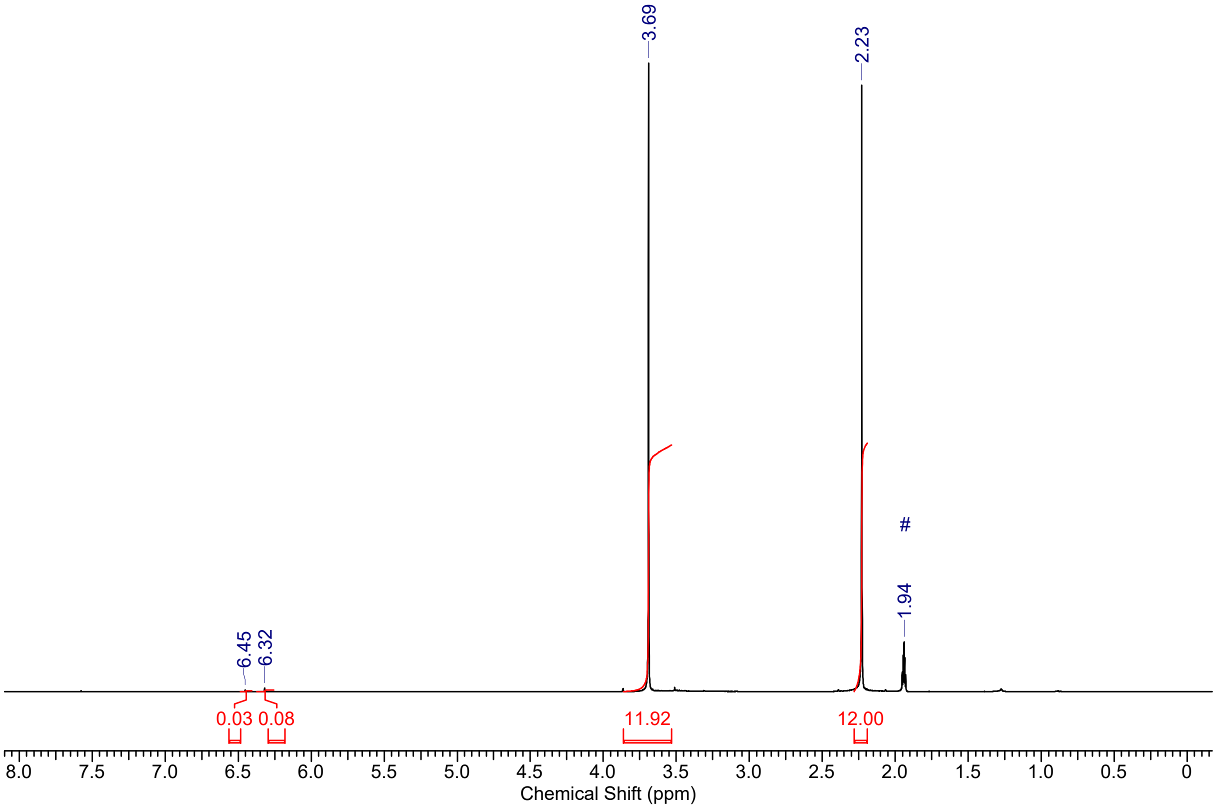

Figure S25. ^{1}H NMR spectrum (400 MHz, CD_3CN #) of **5-d$_3$**.

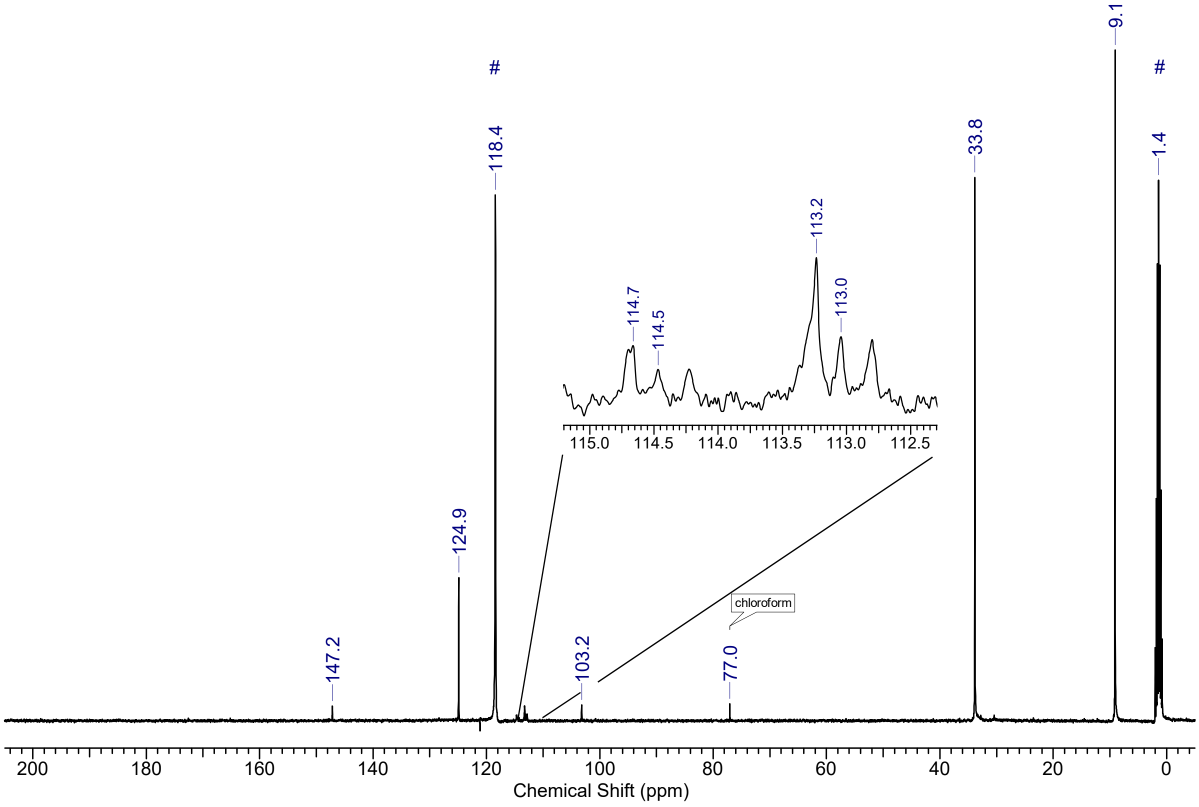

Figure S26. ^{13}C NMR spectrum (100 MHz, CD_3CN #) of **5-d$_3$**.

Spectra of **6**

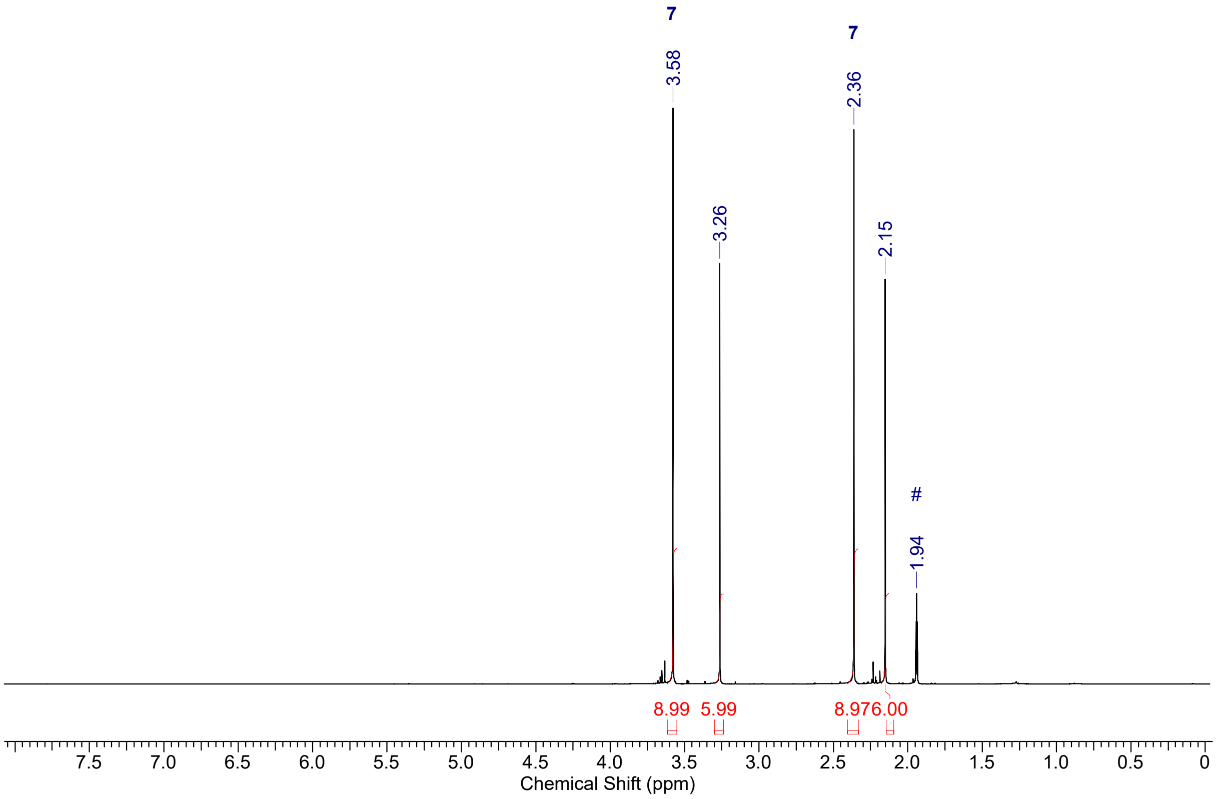

Figure S27. ^{1}H NMR spectrum (700 MHz, CD_3CN #) of **6** and **7**.

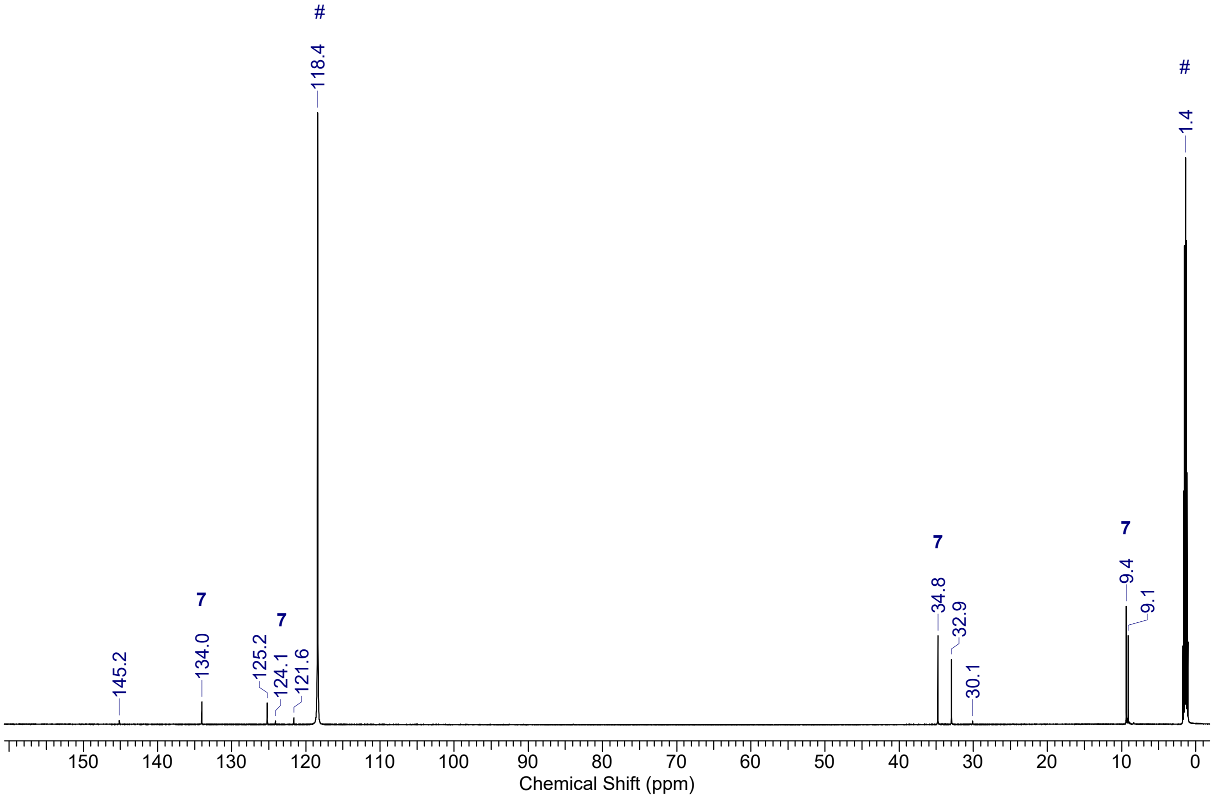

Figure S28. ^{13}C NMR spectrum (176 MHz, CD_3CN #) of **6** and **7**.

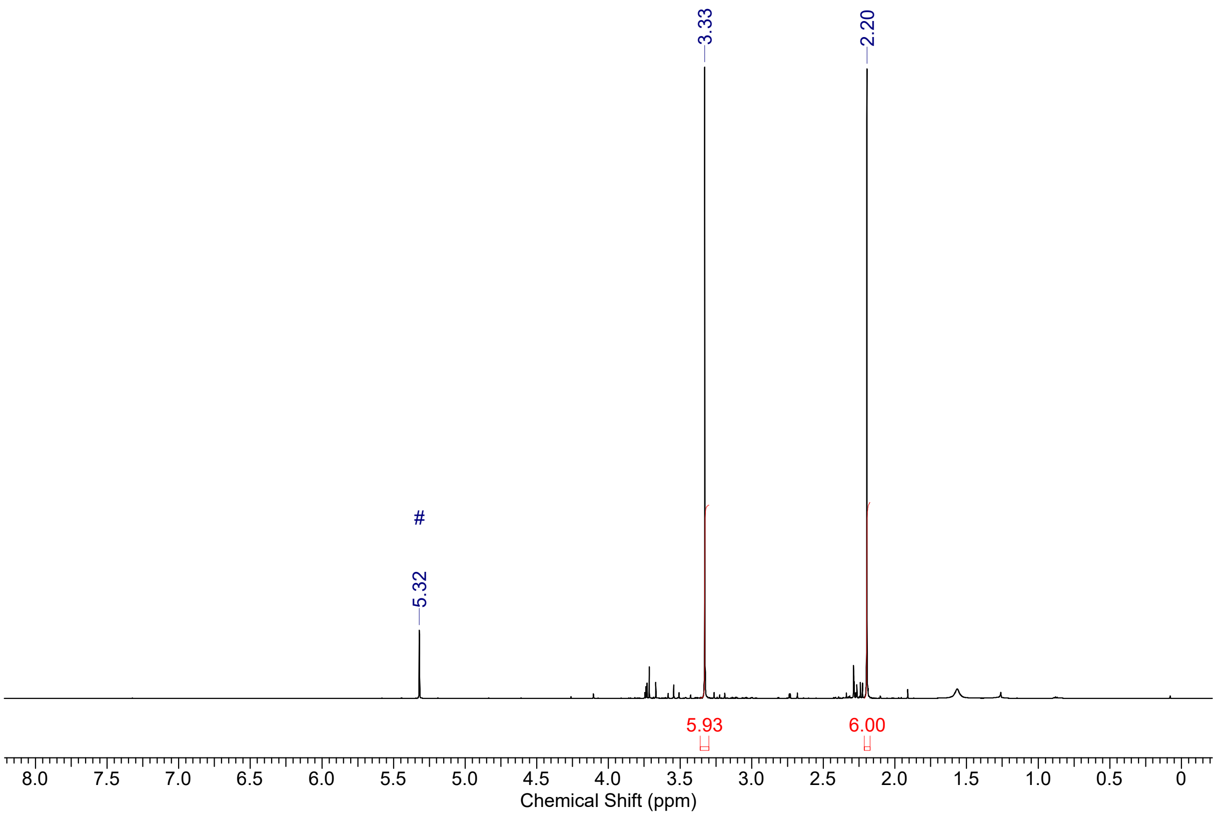

Figure S29. ^{1}H NMR spectrum (700 MHz, CD_2Cl_2 #) of **6**.

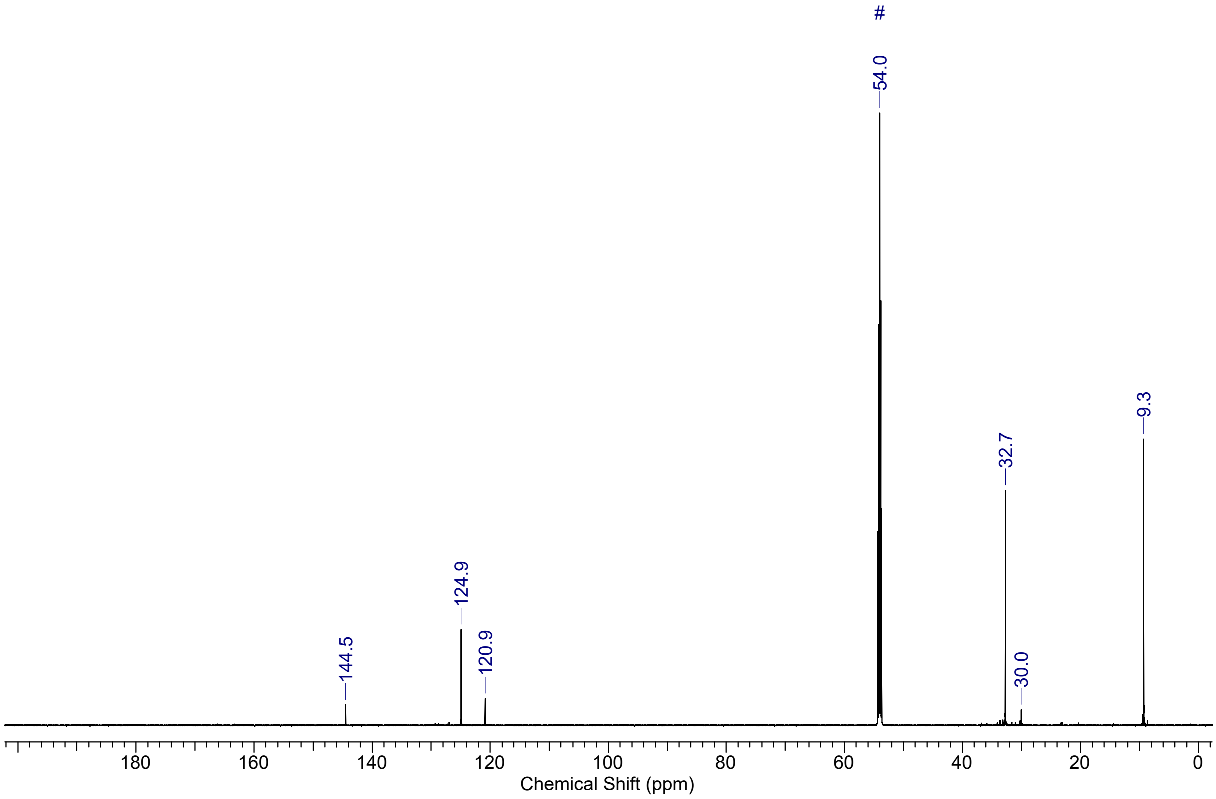

Figure S30. ^{13}C NMR spectrum (176 MHz, CD_2Cl_2 #) of **6**.

Spectra of 7

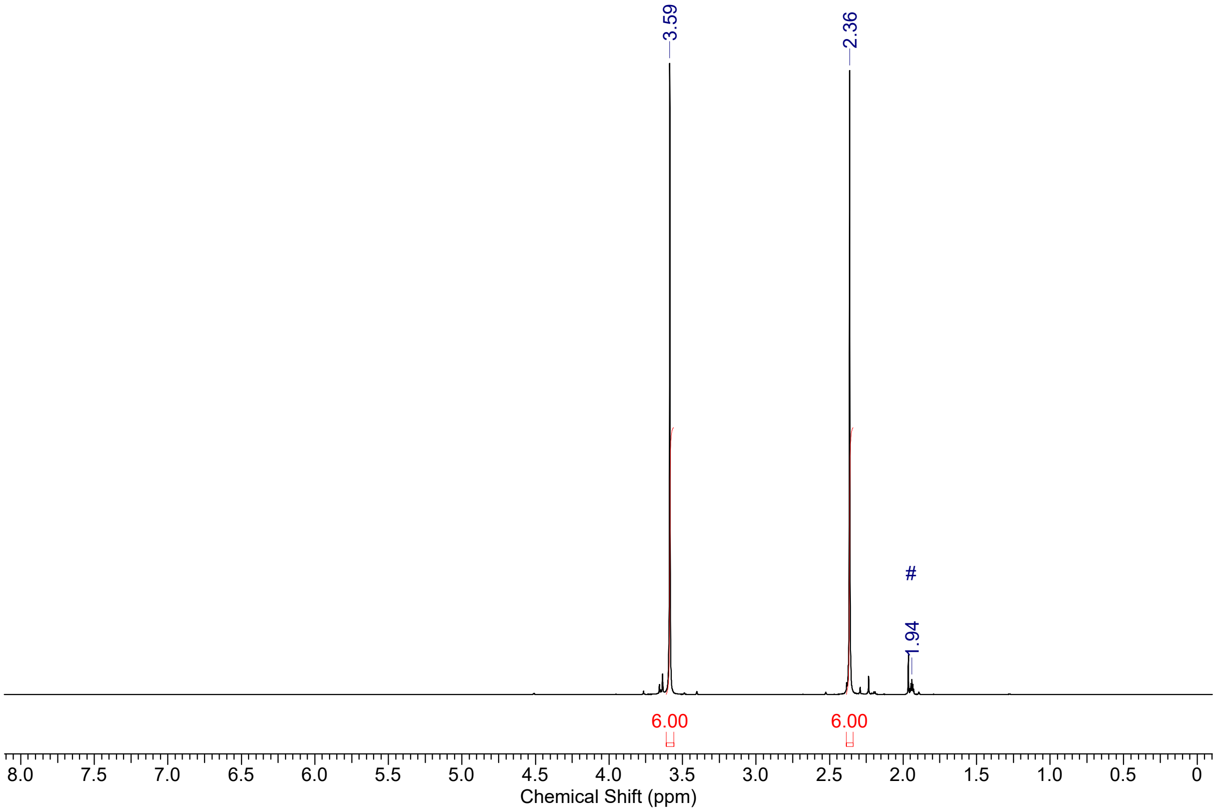

Figure S31. ^{1}H NMR spectrum (400 MHz, CD_3CN #) of **7**.

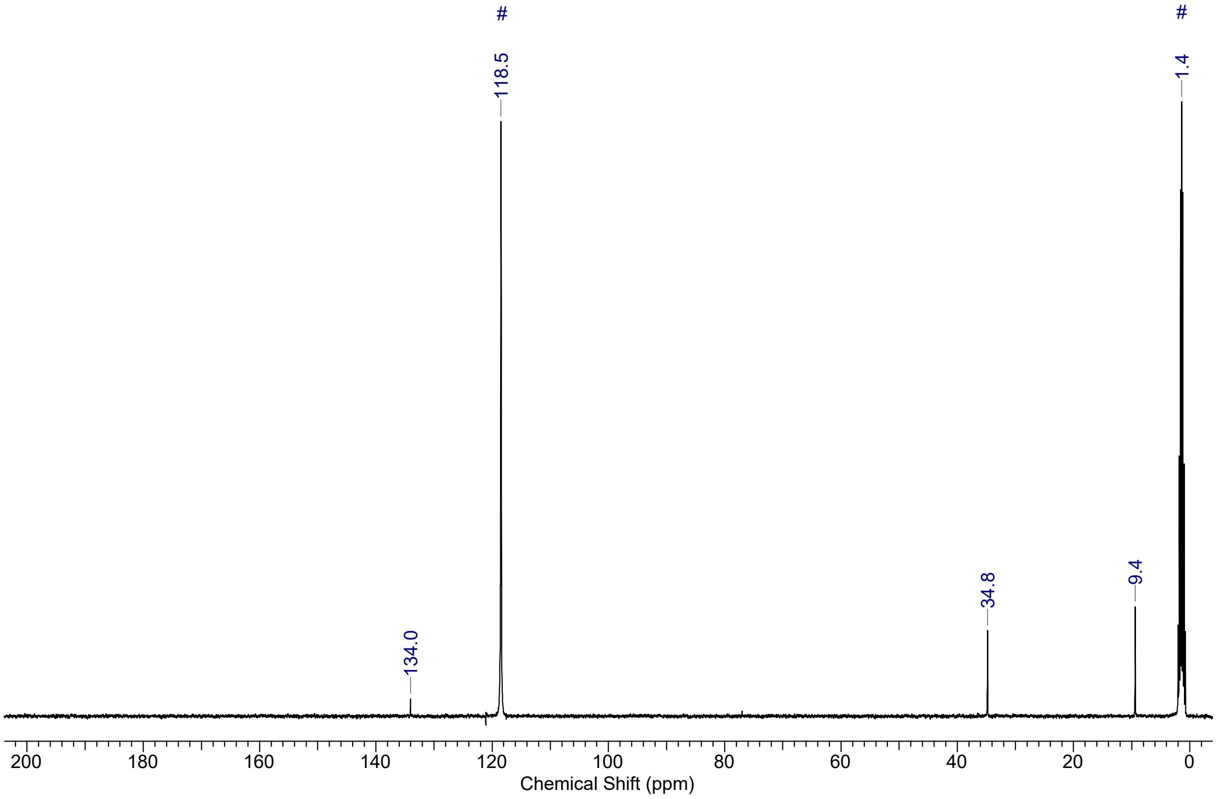

Figure S32. ^{13}C NMR spectrum (100 MHz, CD_3CN #) of **7**.

Spectra of 9

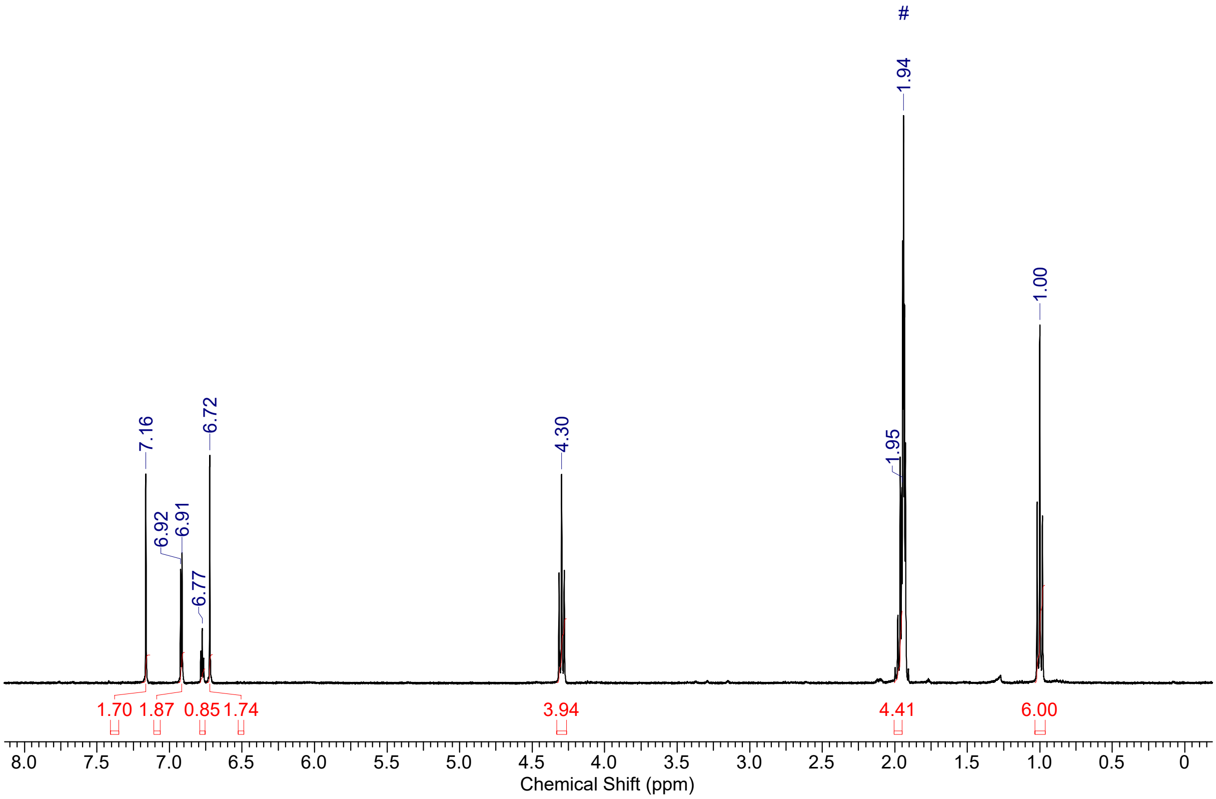

Figure S33. ^{1}H NMR spectrum (400 MHz, CD_3CN #) of **9**.

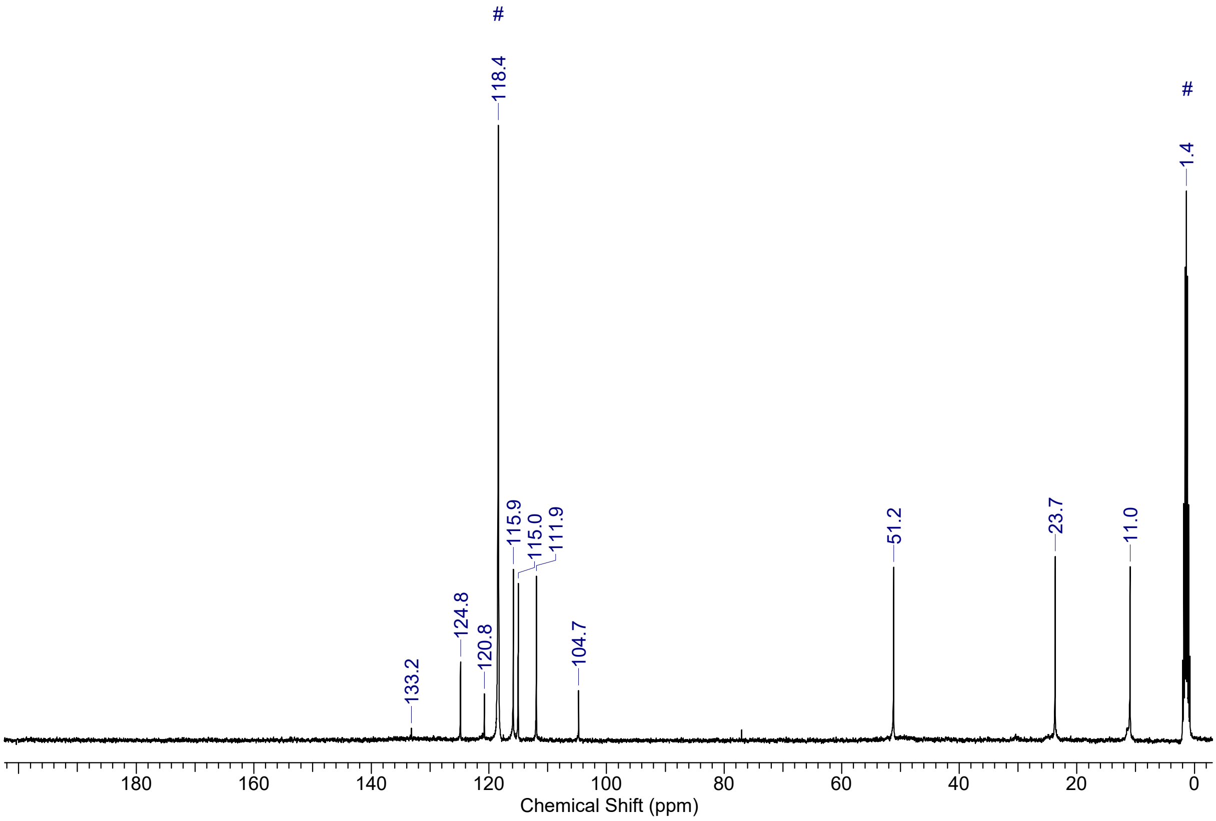

Figure S34. ^{13}C NMR spectrum (100 MHz, CD_3CN #) of **9**.

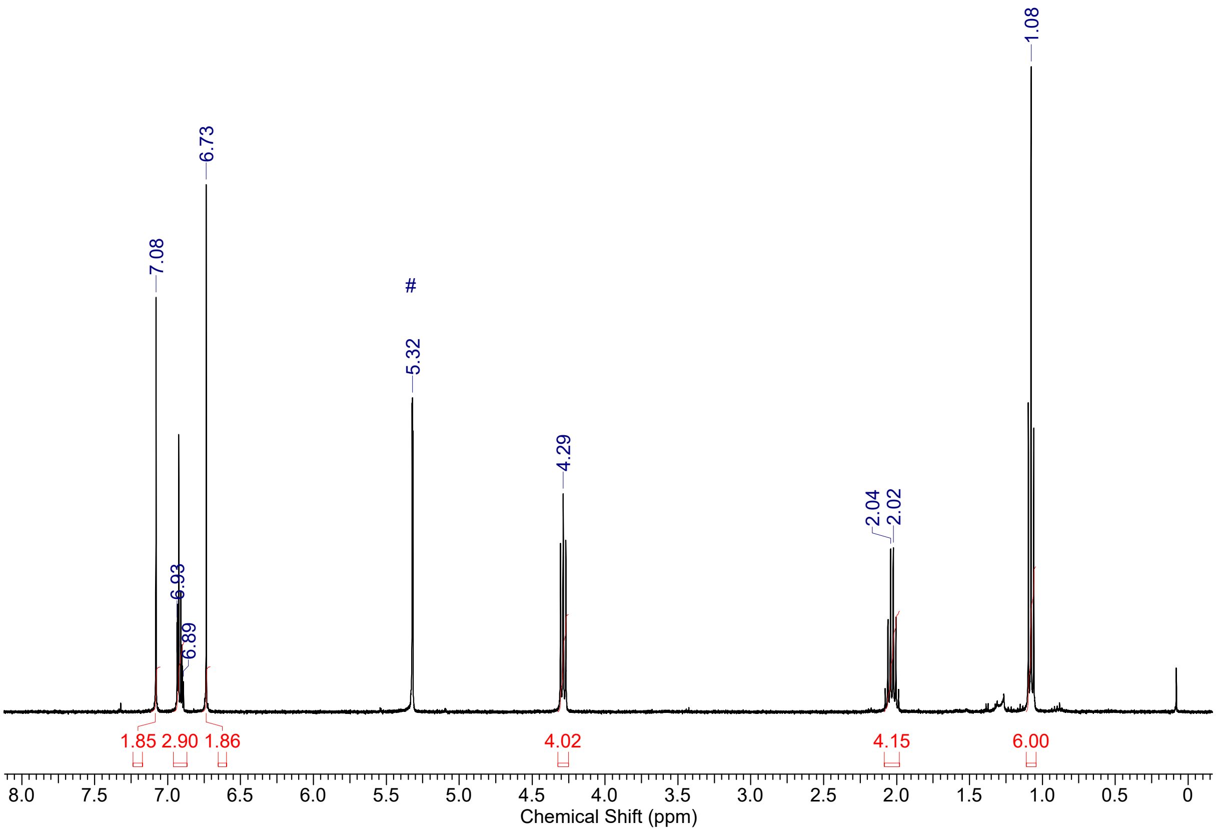

Figure S35. ^{1}H NMR spectrum (400 MHz, CD_2Cl_2 #) of **9**.

Spectra of **9-d$_2$**

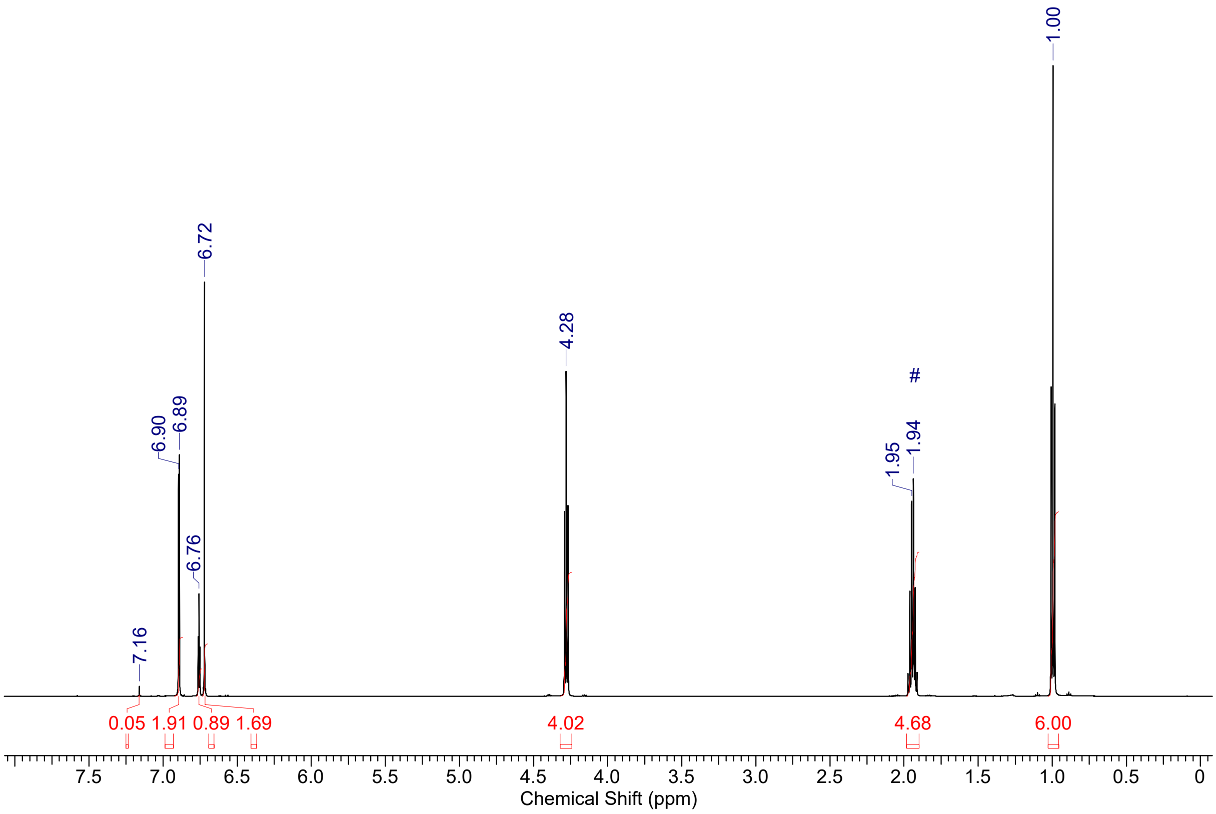

Figure S36. ^{1}H NMR spectrum (600 MHz, CD_2Cl_2 #) of **9-d$_2$**.

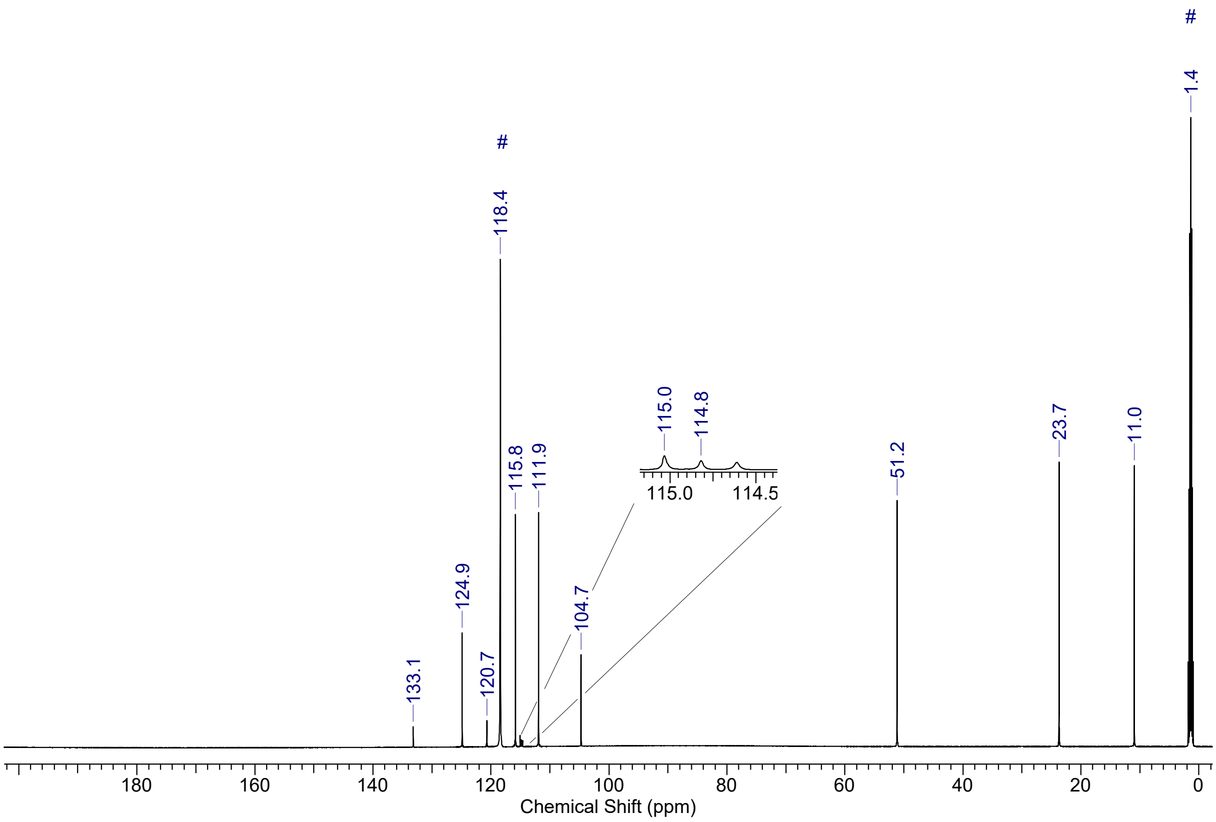

Figure S37. ^{13}C NMR spectrum (151 MHz, CD_3CN #) of **9-d$_2$**.

Spectra of 12 and 13

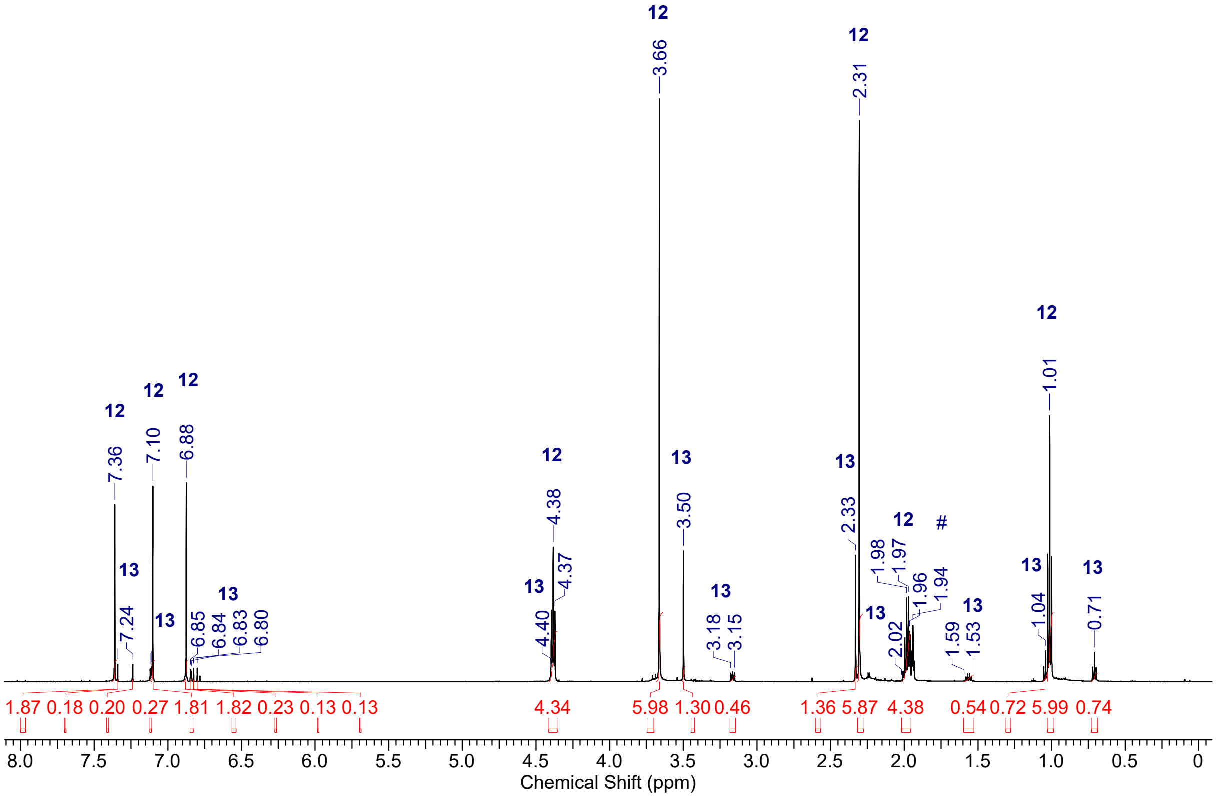

Figure S38. ^{1}H NMR spectrum (600 MHz, CD_3CN #) of **12** and **13**.

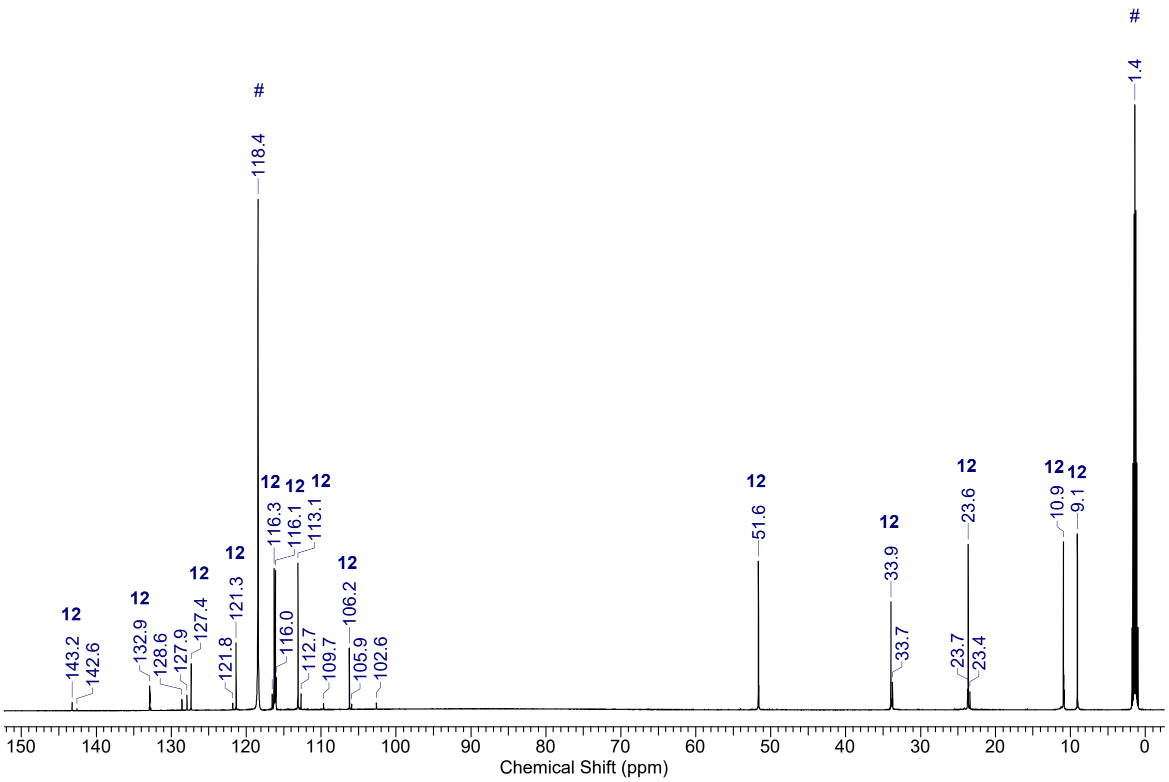

Figure S39. ^{13}C NMR spectrum (151 MHz, CD_3CN #) of **12** and **13**.

Spectra of 12-d_2 and 13-d_2

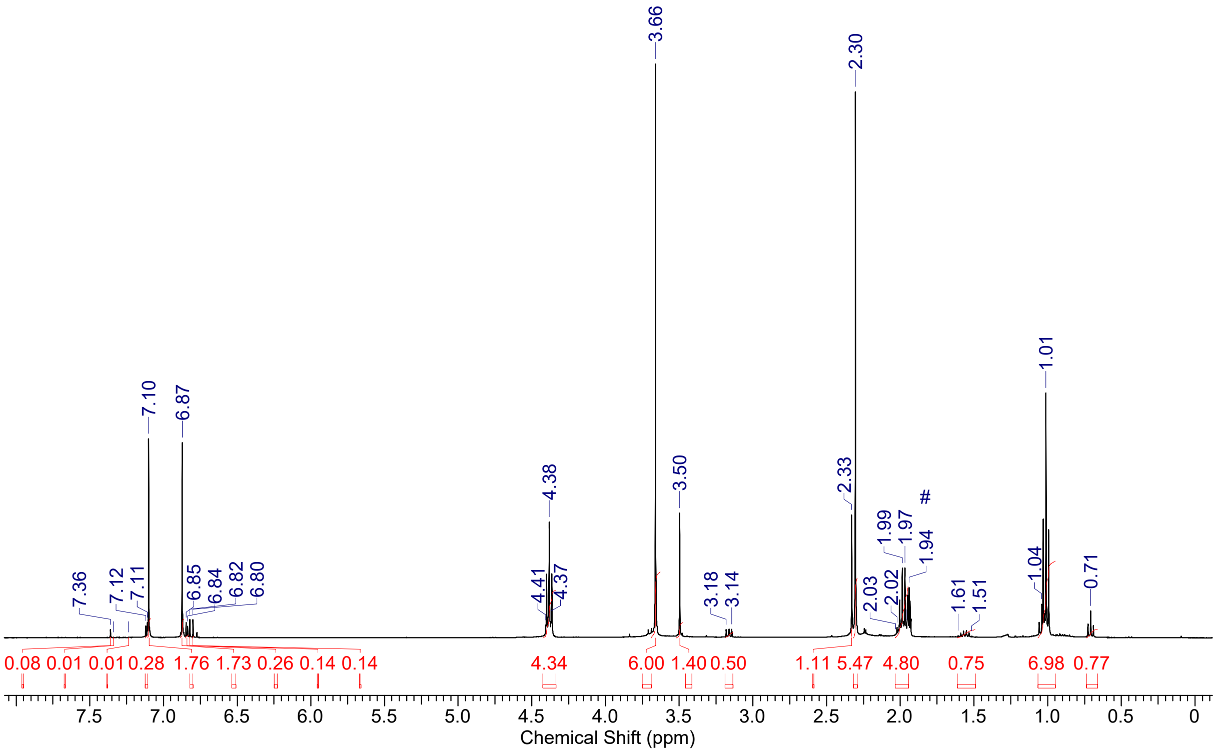

Figure S40. ^{1}H NMR spectrum (400 MHz, CD_3CN #) of **12-d_2** and **13-d_2**.

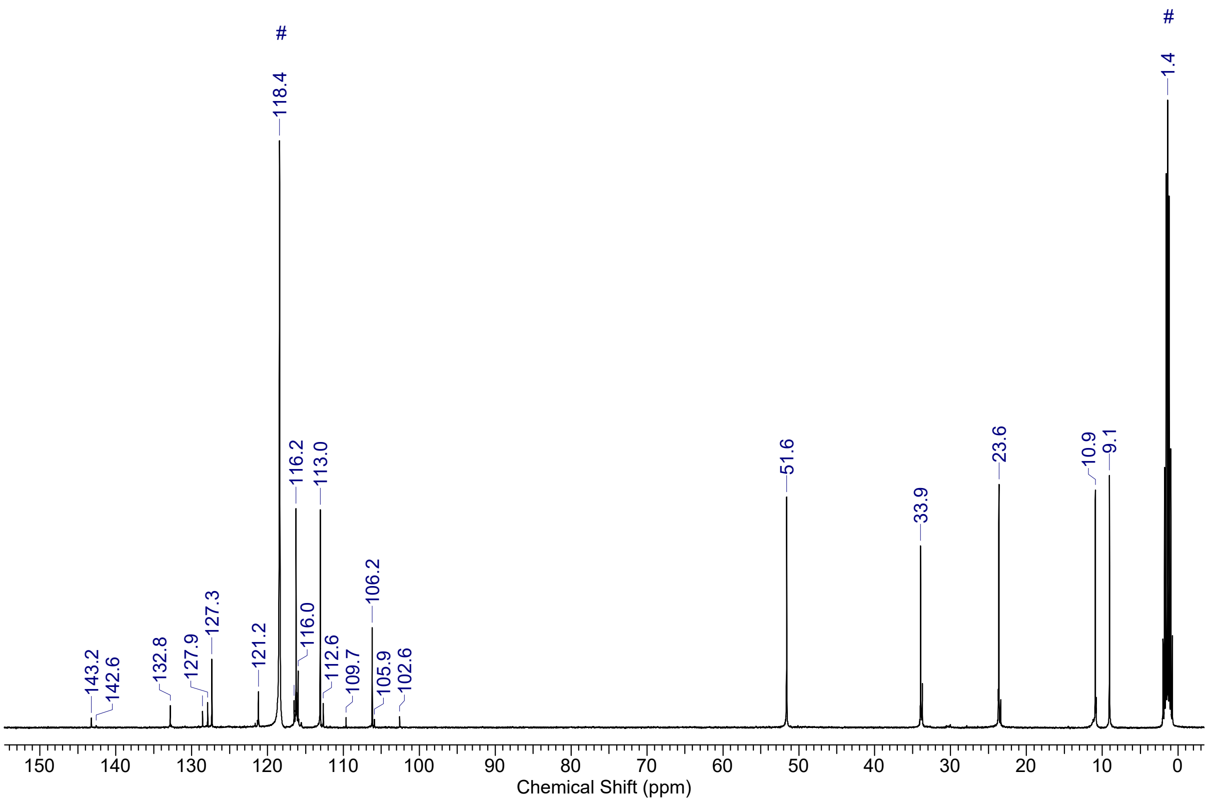

Figure S41. ^{13}C NMR spectrum (100 MHz, CD_3CN #) of **12-d$_2$** and **13-d$_2$**.

Spectra of 15

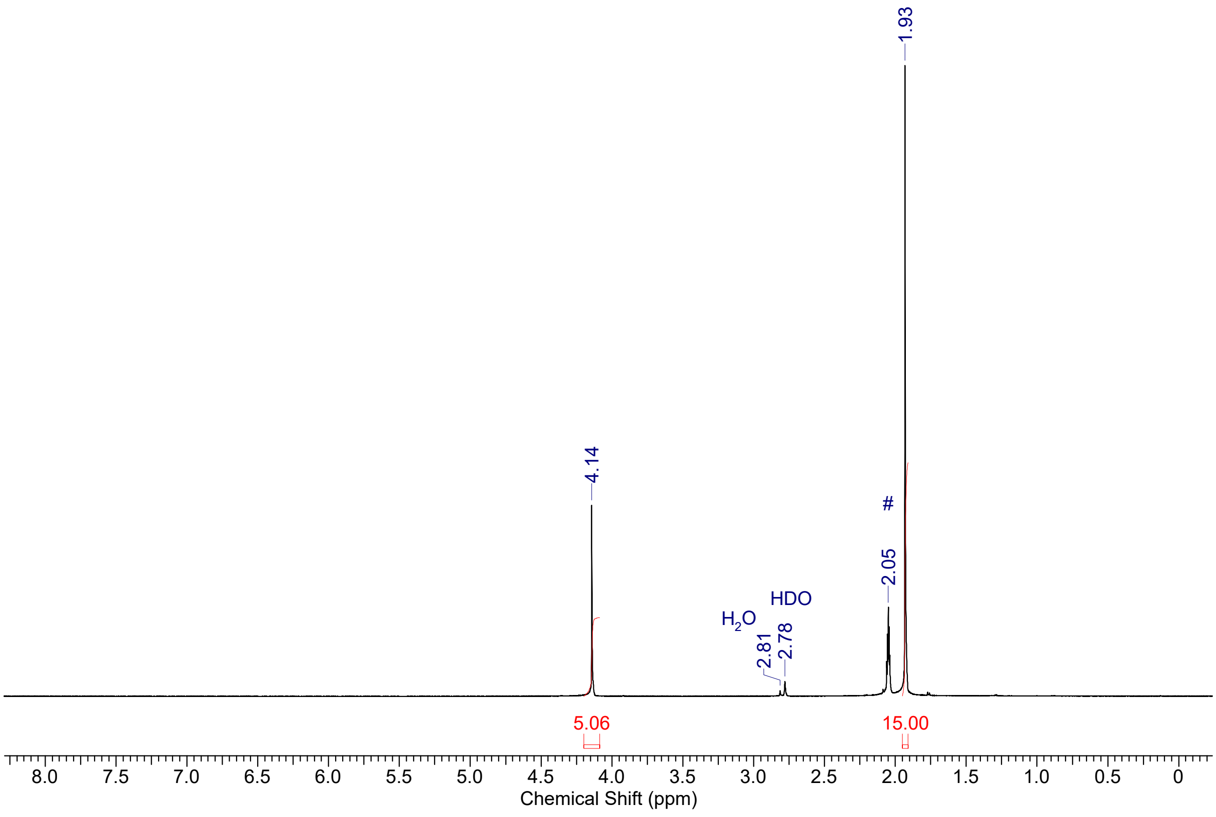

Figure S42: ^1H NMR spectrum (400 MHz, acetone-d$_6$ #) of **15**.

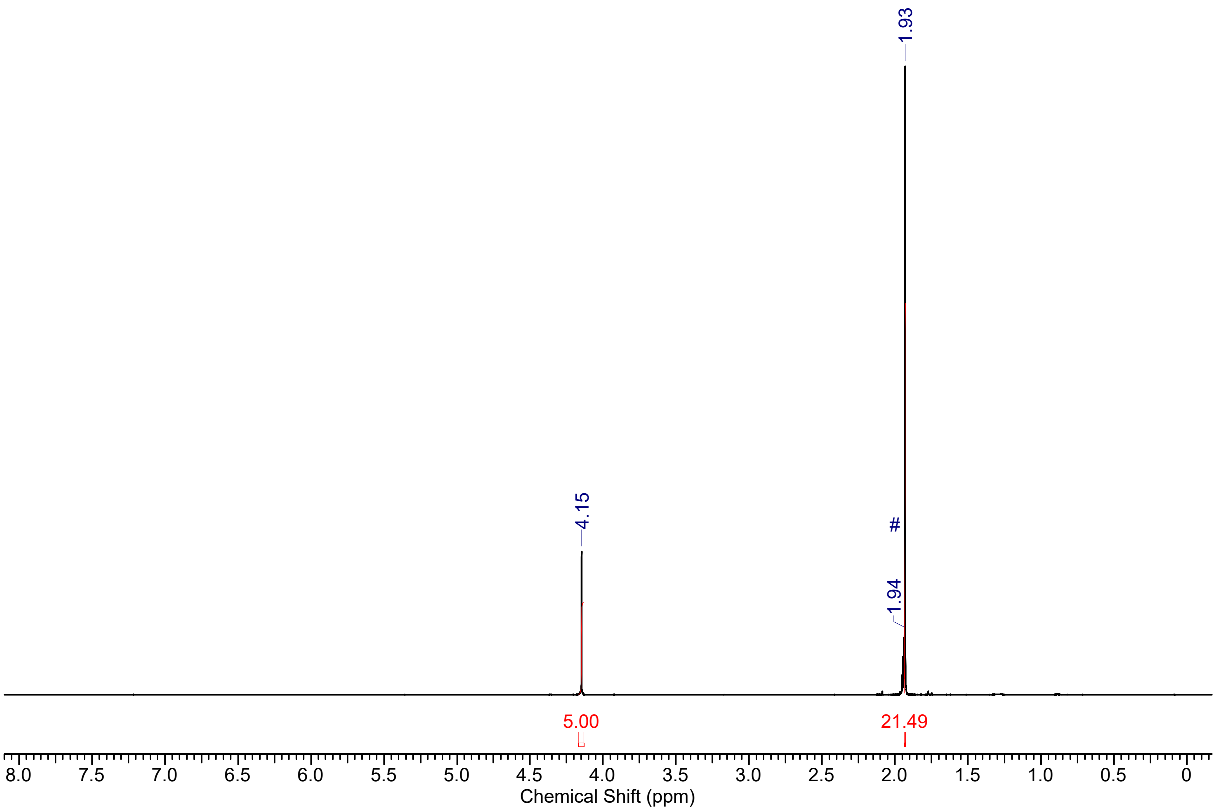

Figure S43: ^{1}H NMR spectrum (400 MHz, CD_3CN #) of **15**.

Spectra of 16

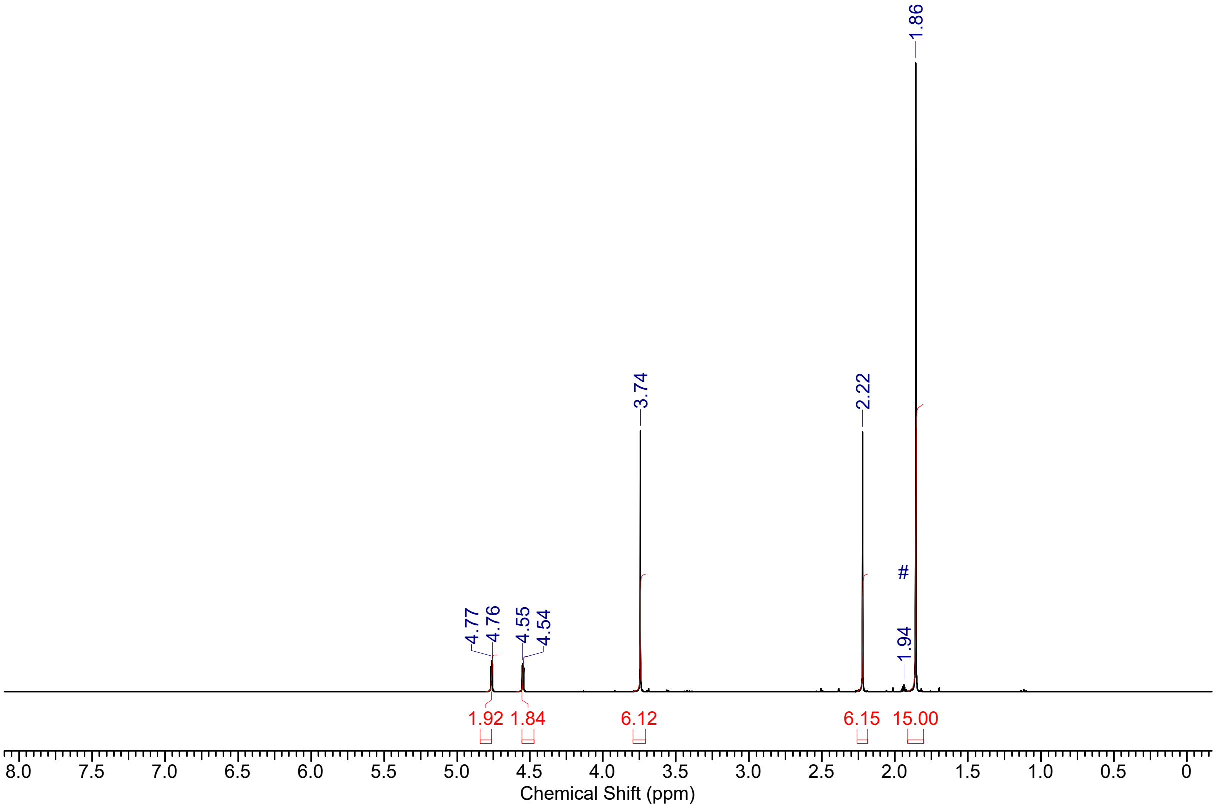

Figure S44: ^{1}H NMR spectrum (400 MHz, CD_3CN #) of **16**.

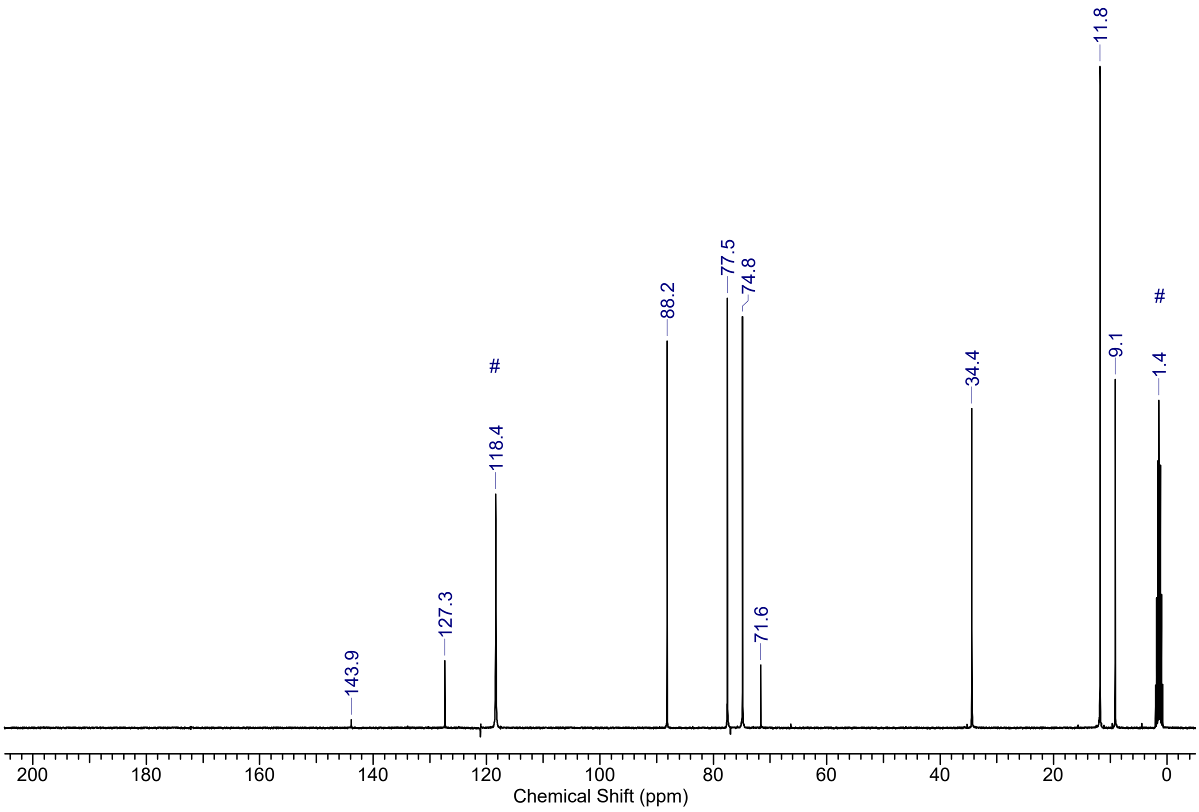

Figure S45: ^{13}C NMR spectrum (100 MHz, CD_3CN #) of **16**.

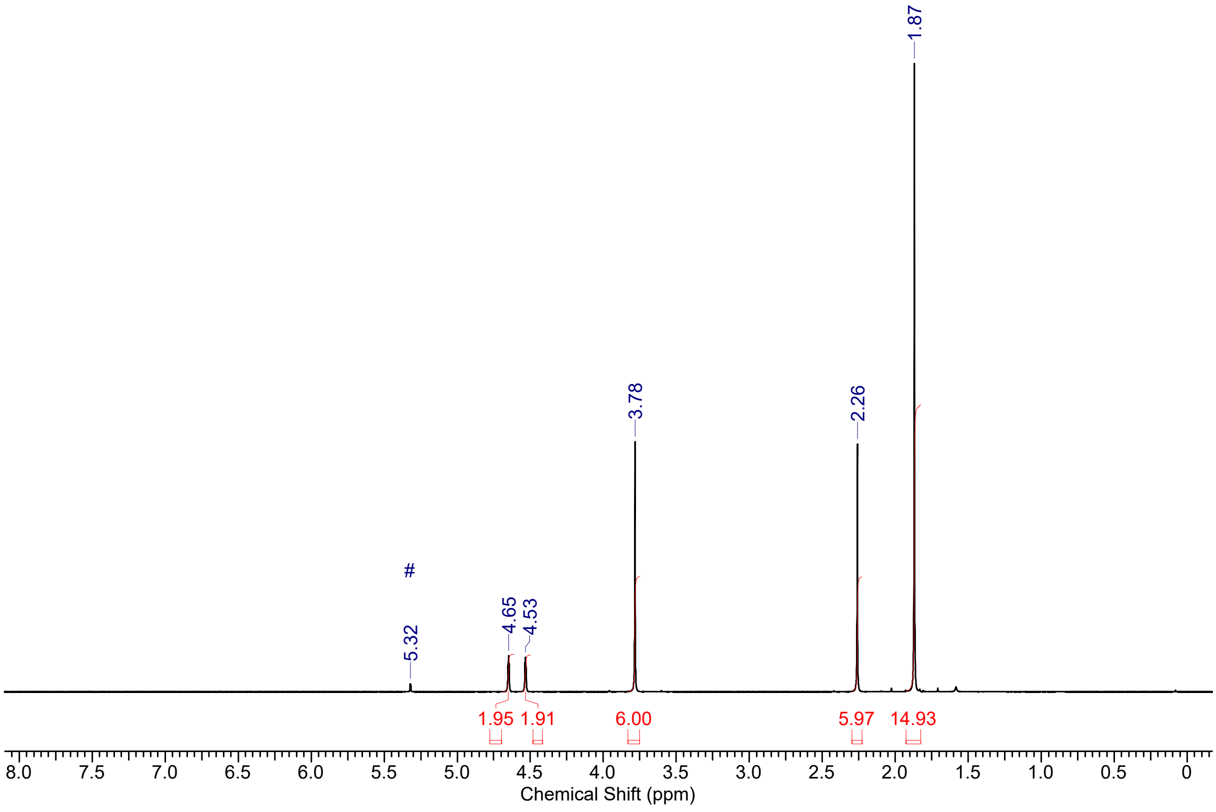

Figure S46: ^{1}H NMR spectrum (400 MHz, CD_2Cl_2 #) of **16**.

Spectra of **17**

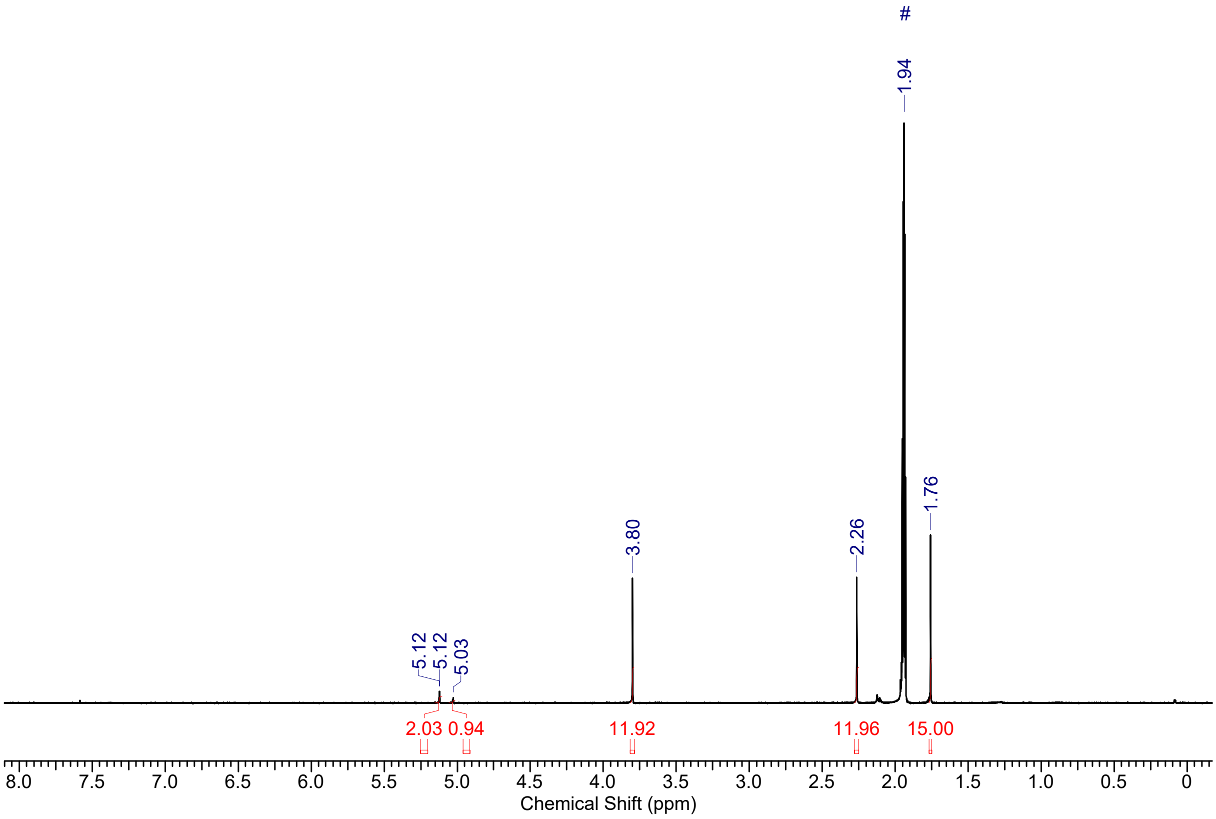

Figure S47: ^{1}H NMR spectrum (400 MHz, CD_3CN #) of **17**.

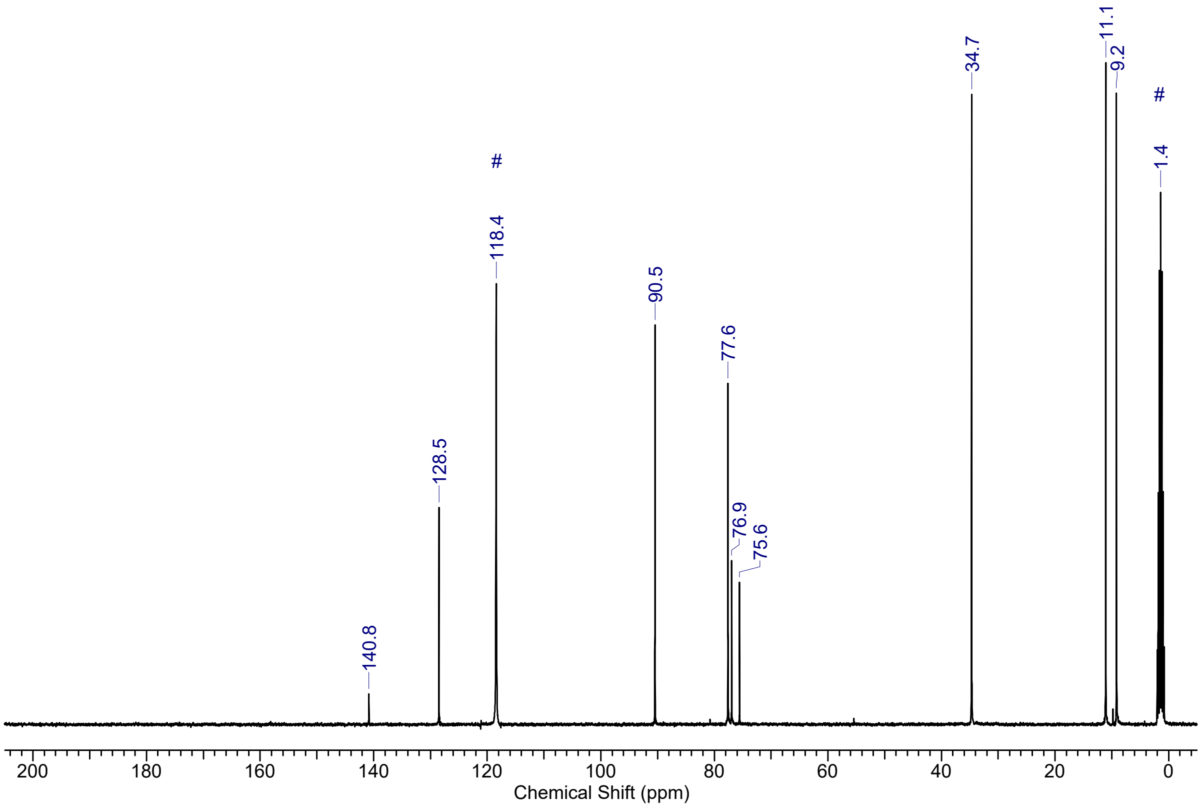

Figure S48: ^{13}C NMR spectrum (100 MHz, CD_3CN #) of **17**.

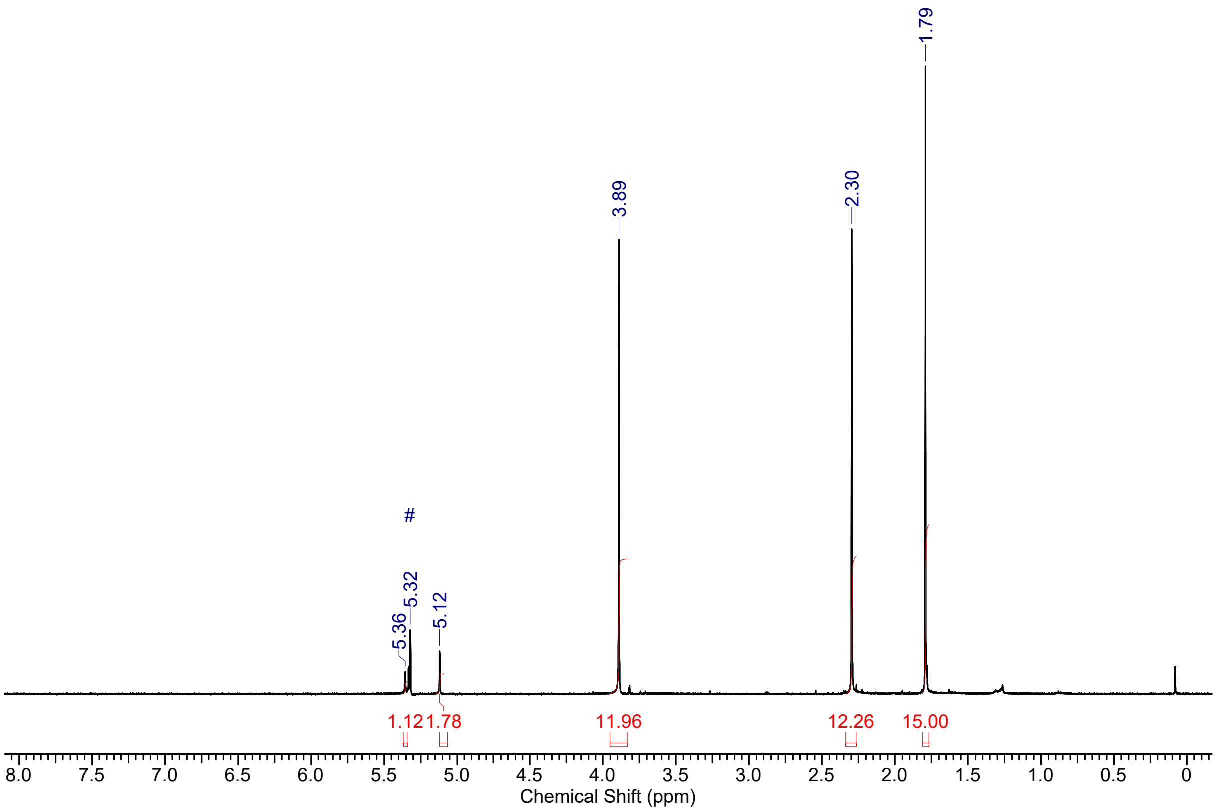

Figure S49: ^{1}H NMR spectrum (400 MHz, CD_2Cl_2 #) of **17**.

Spectra of 18

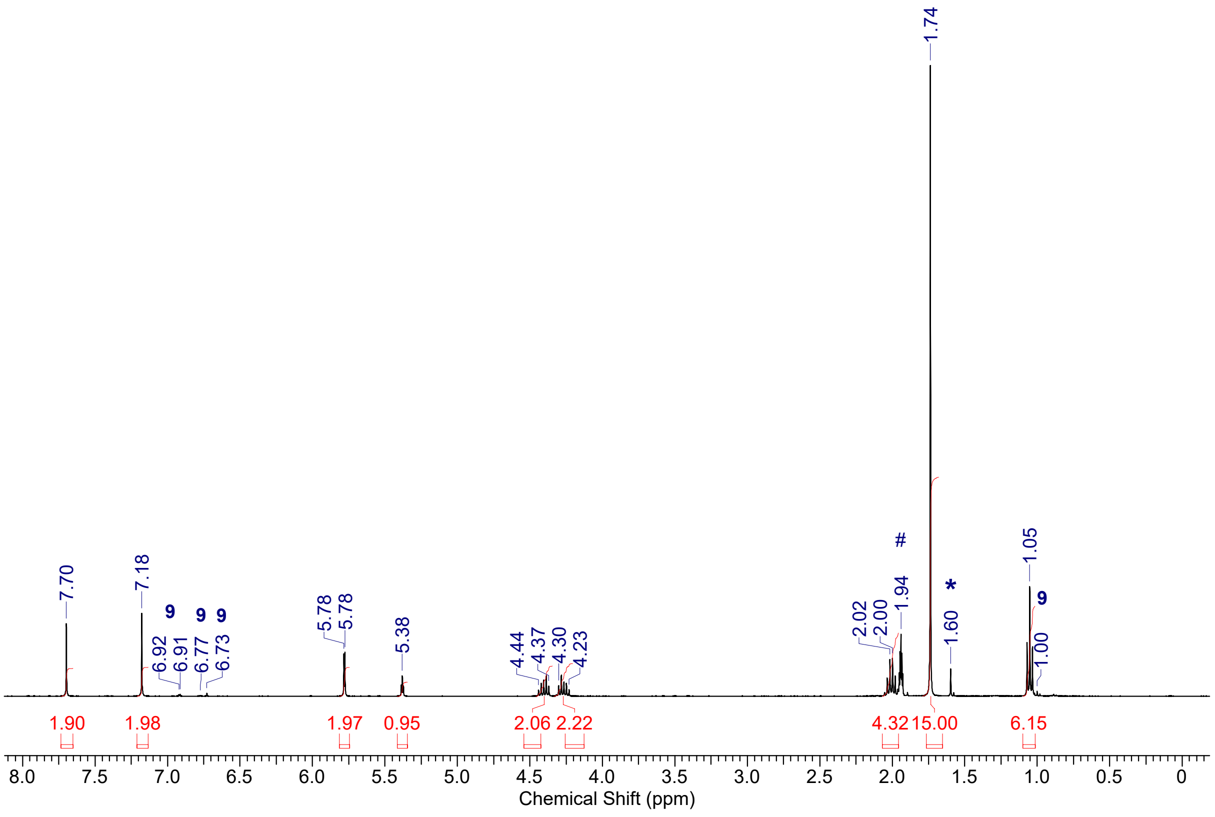

Figure S50. ^{1}H NMR spectrum (400 MHz, CD_3CN #) of **18** and formed **9** and [RuCp*(CD_3CN)$_3$](PF_6) *.

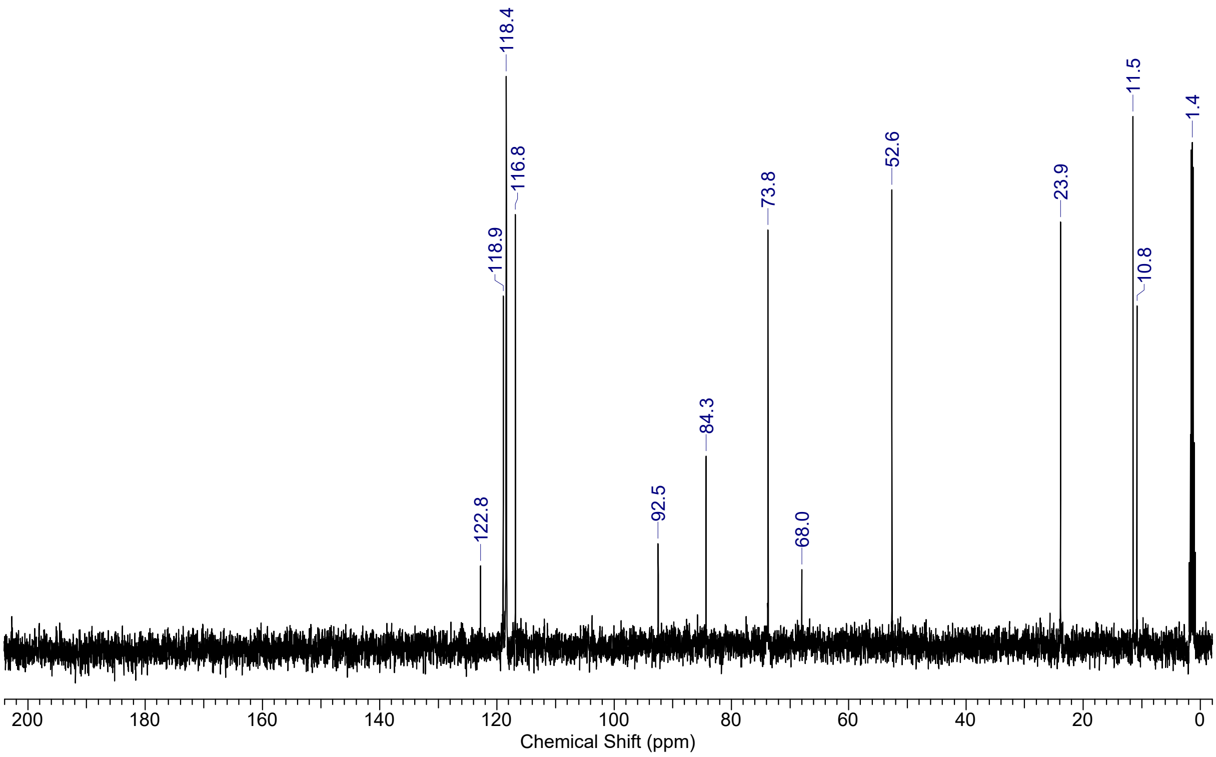

Figure S51. ^{13}C NMR spectrum (100 MHz, CD_3CN #) of **18**.

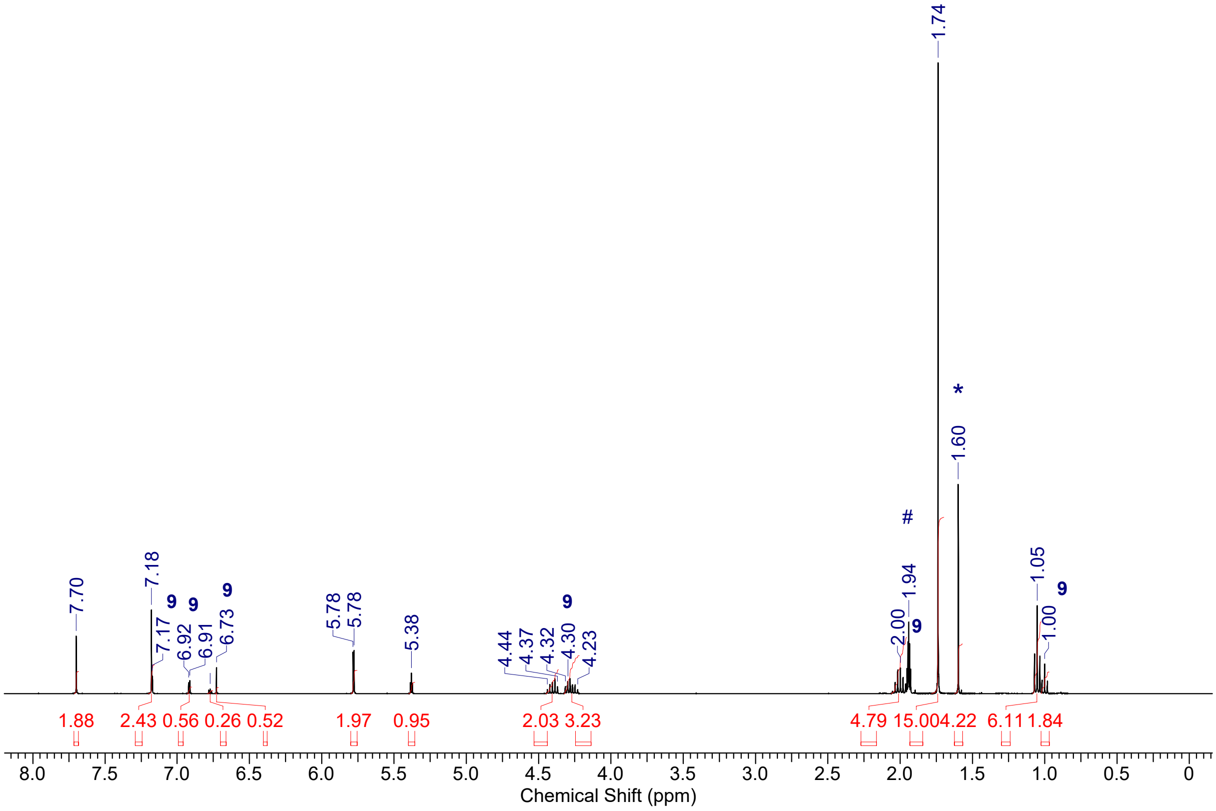

Figure S52. ^{1}H NMR spectrum (400 MHz, CD_3CN #) of the chemical equilibirum of **18** and **9** and $[RuCp^*(CD_3CN)_3](PF_6)$ *.

Spectra of **19**

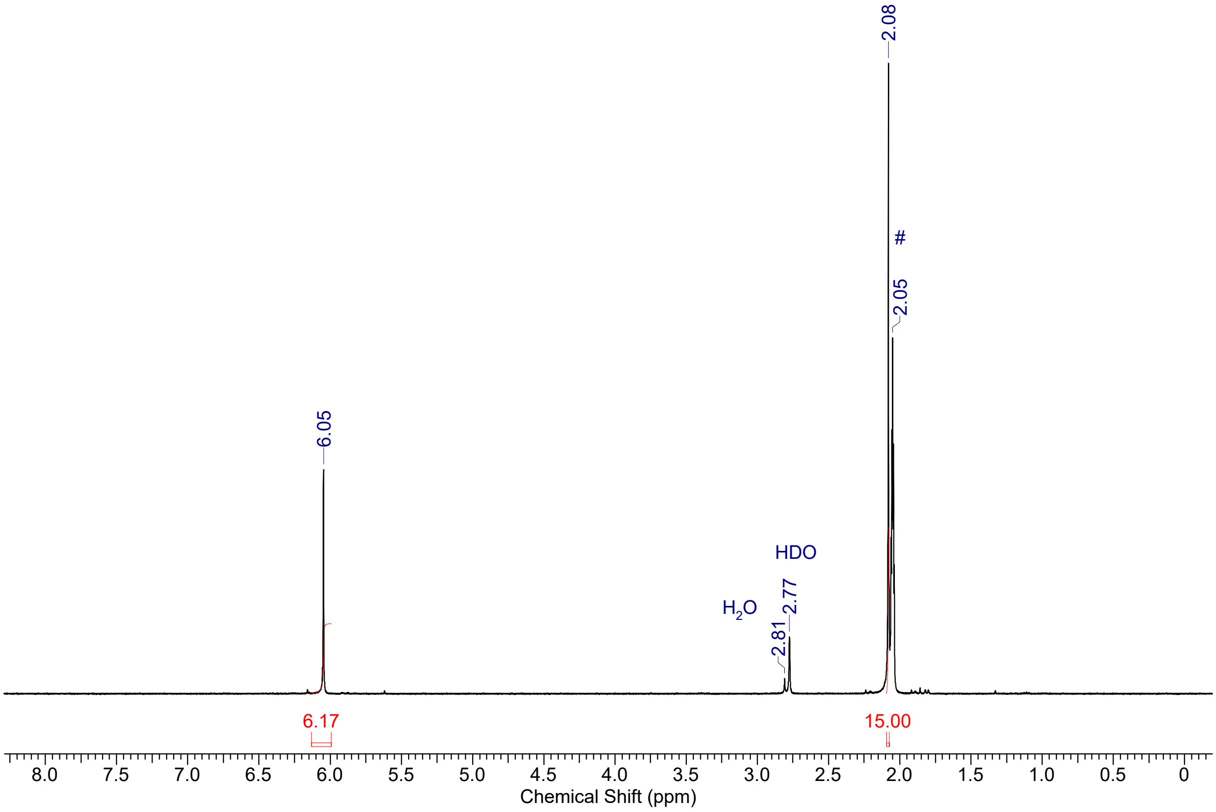

Figure S53. ^{1}H NMR spectrum (400 MHz, acetone-d_6 #) of **19**.

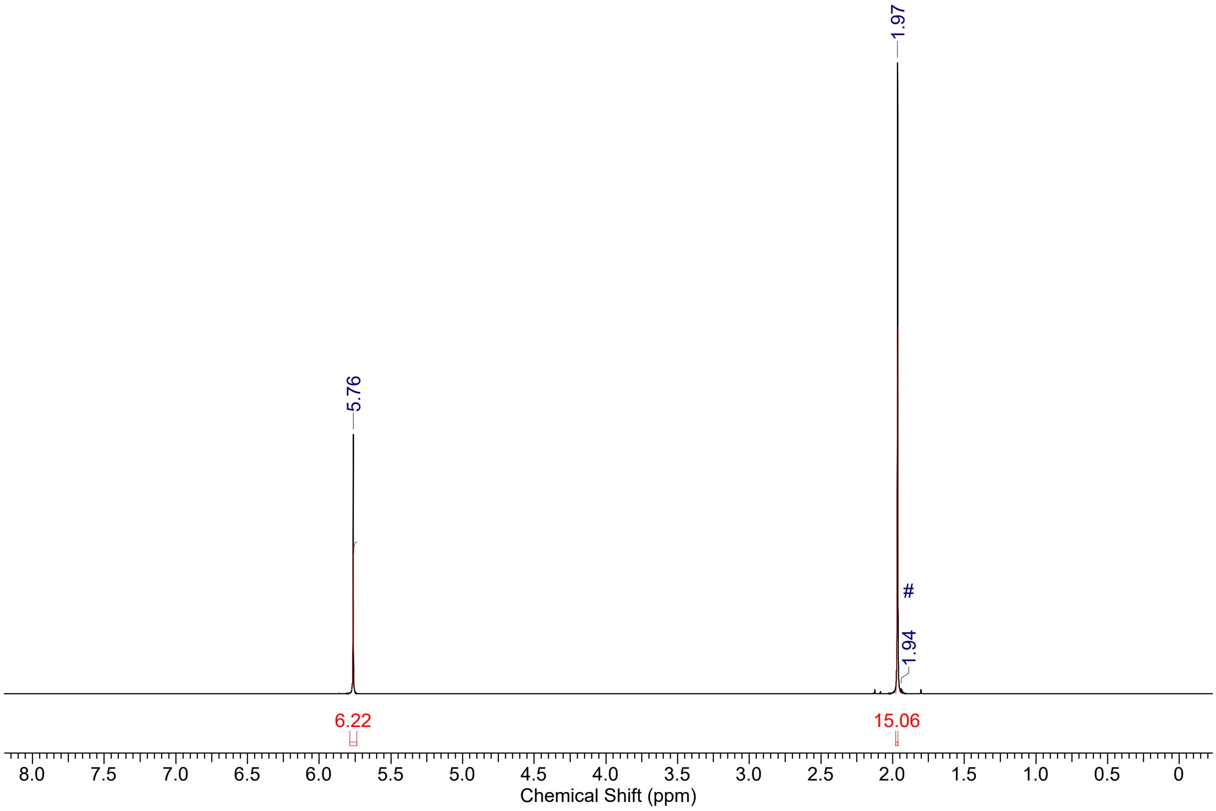

Figure S54. ^{1}H NMR spectrum (400 MHz, CD_3CN #) of **19**.

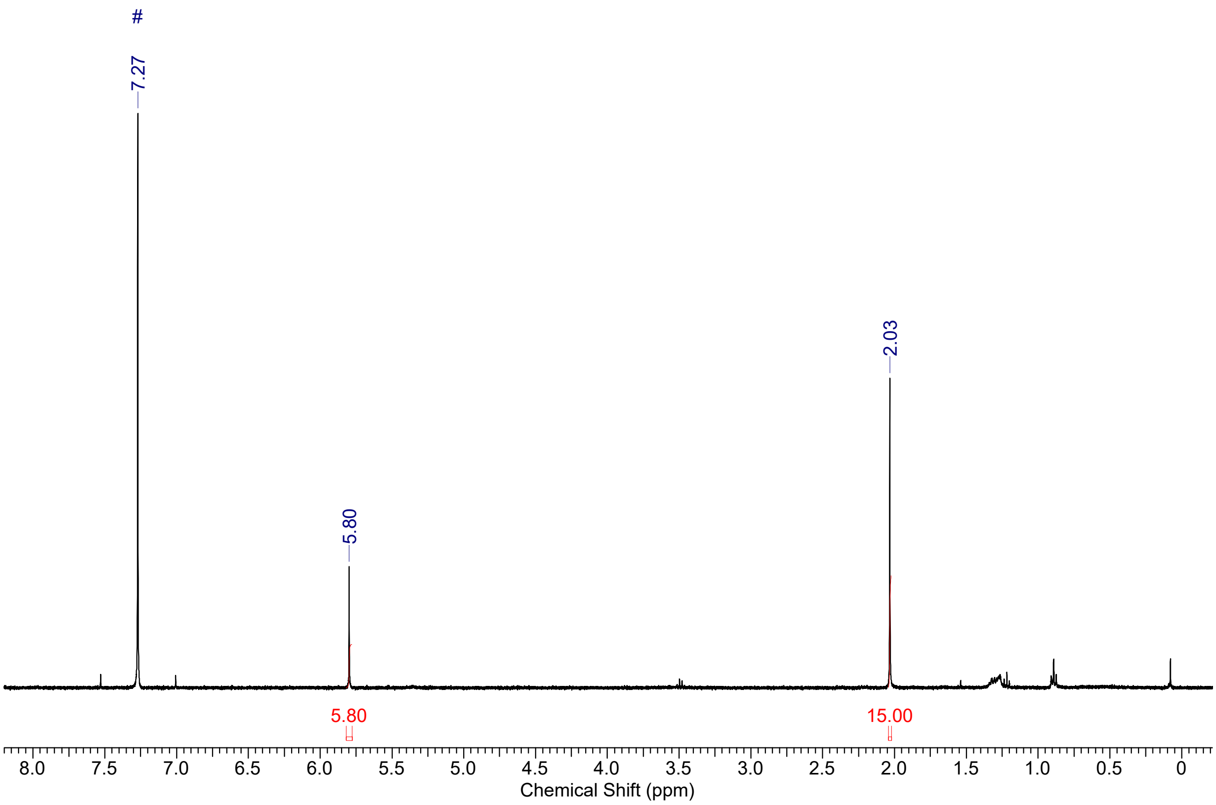

Figure S55. ^{1}H NMR spectrum (400 MHz, $CDCl_3$ #) of **19**.

Spectra of 20

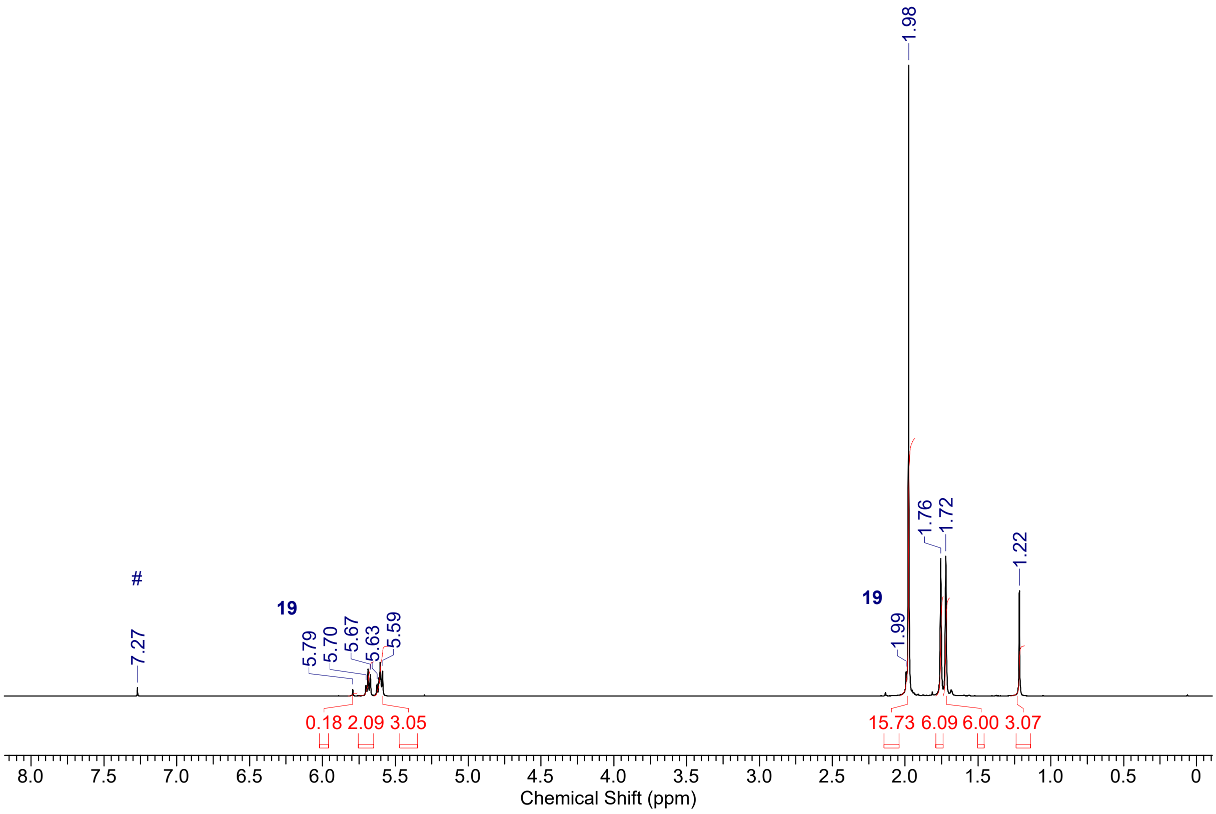

Figure S56. ^{1}H NMR spectrum (400 MHz, $CDCl_3$ #) of **20** with small amount of **19**.

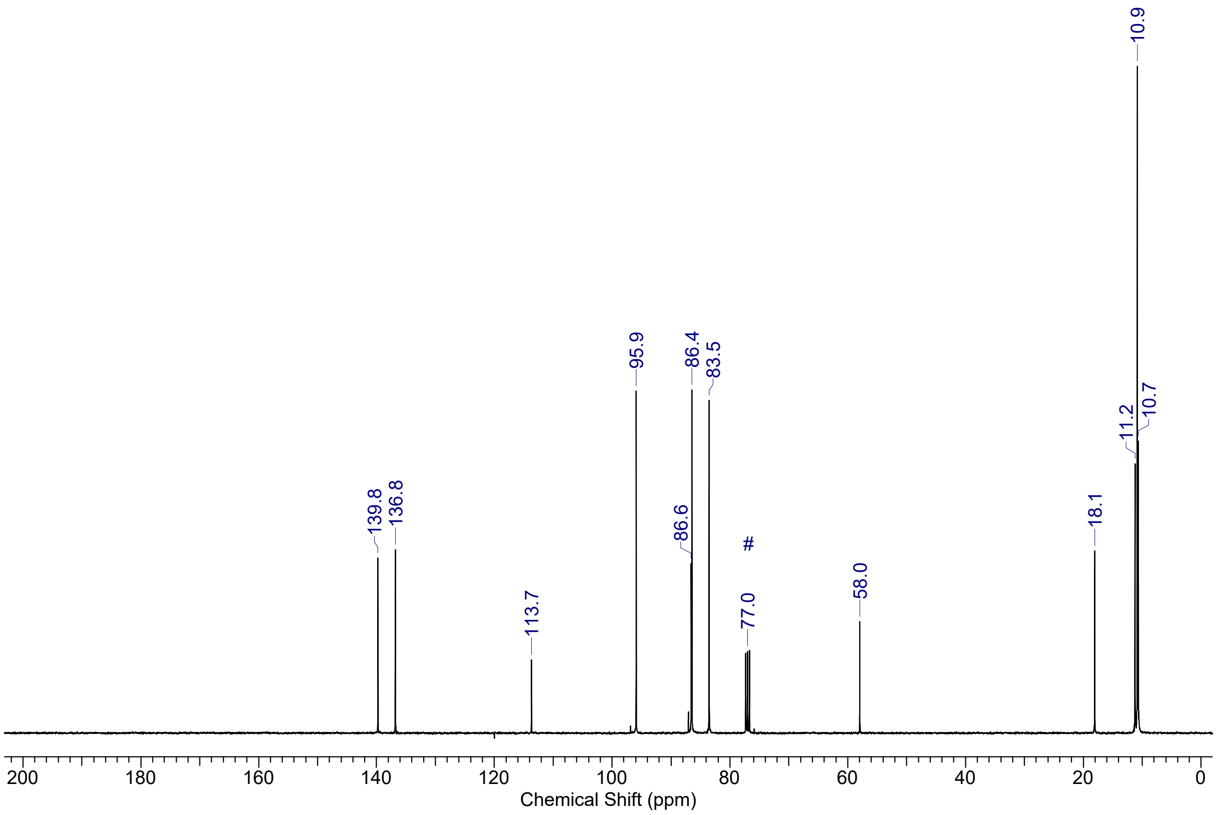

Figure S57. ^{13}C NMR spectrum (100 MHz, $CDCl_3$ #) **20**.

3. IR spectra

Spectrum of 5

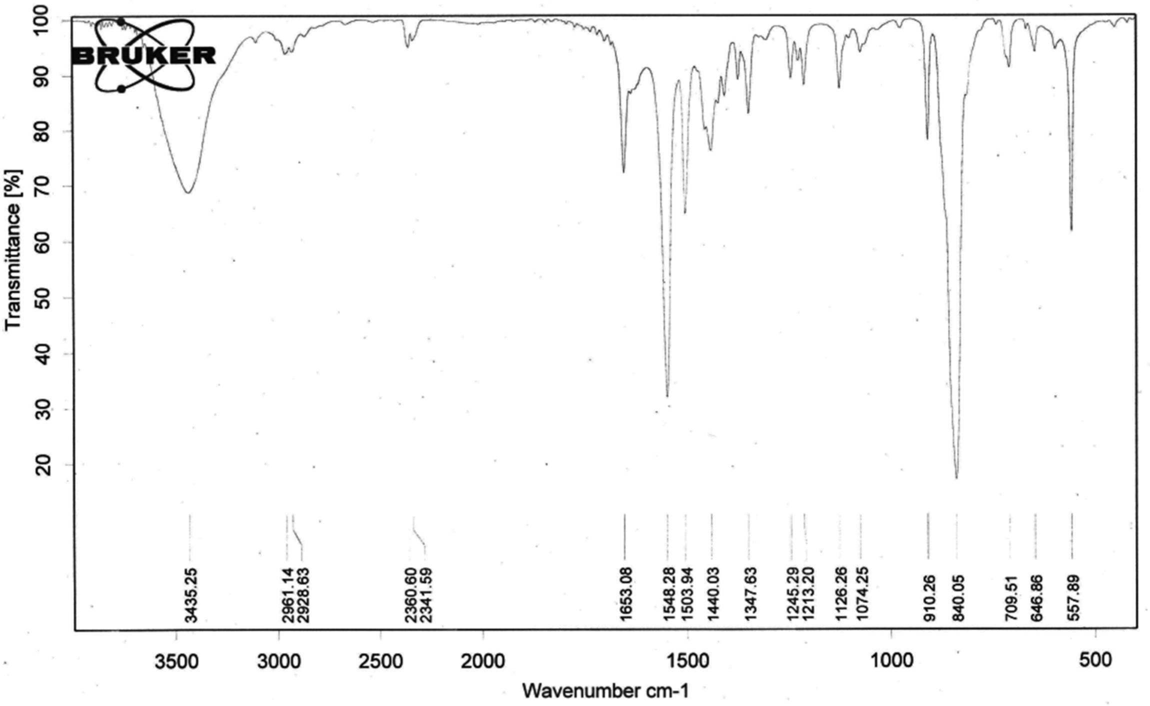

Figure S58. IR spectrum (KBr) of **5**.

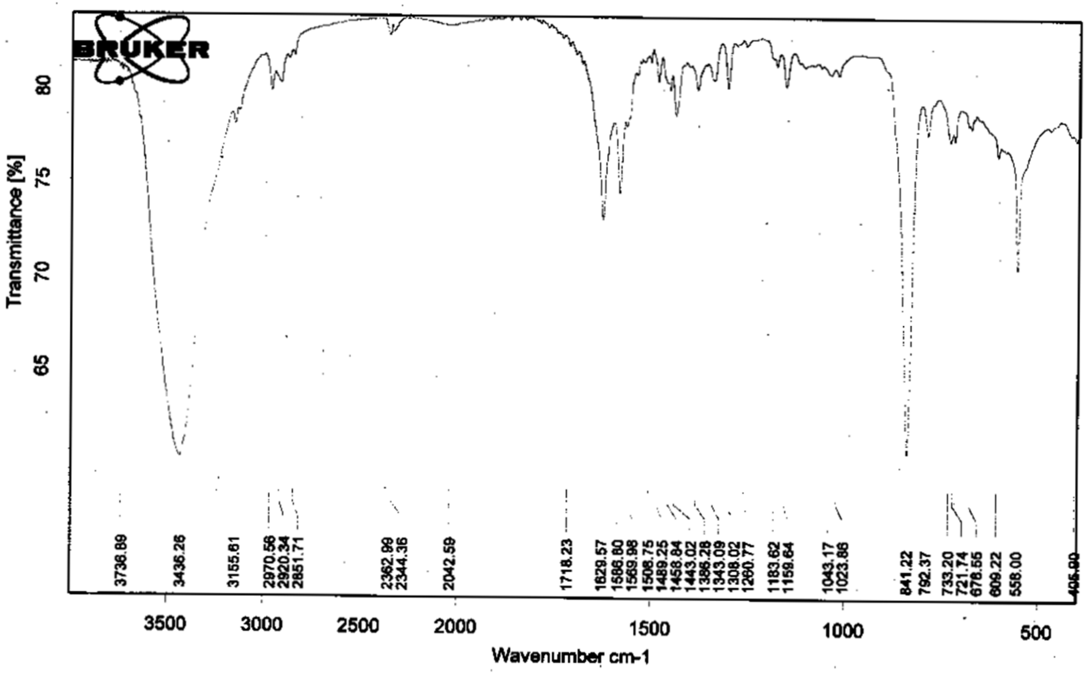

Figure S59. IR spectrum (KBr) of **9**.

Spectrum of 16

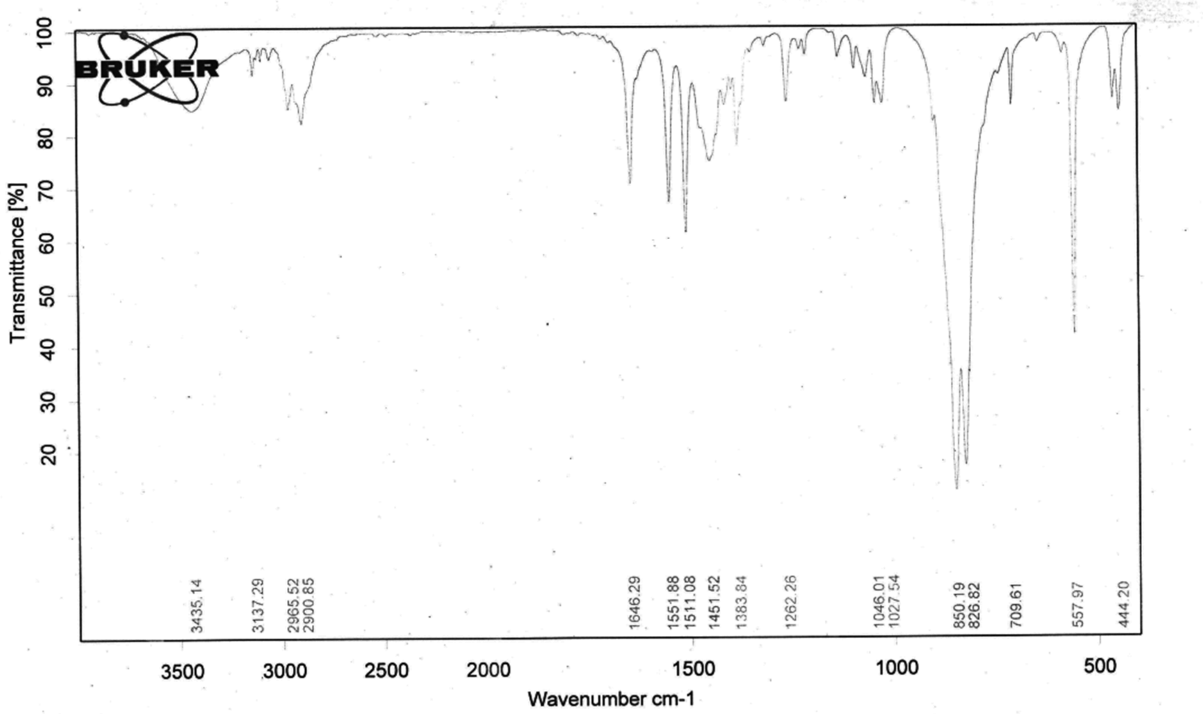

Figure S60. IR spectrum (KBr) of **16**.

Spectrum of 17

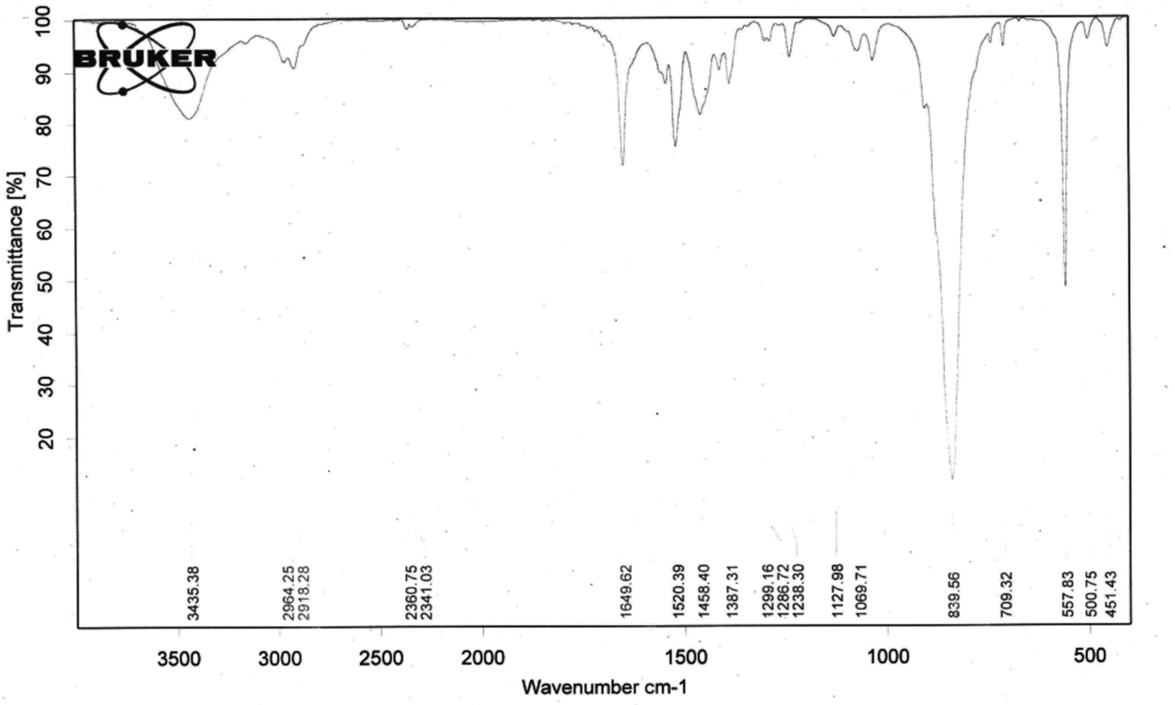

Figure S61. IR spectrum (KBr) of **17**.

4. UV/VIS spectra

Spectra of 2

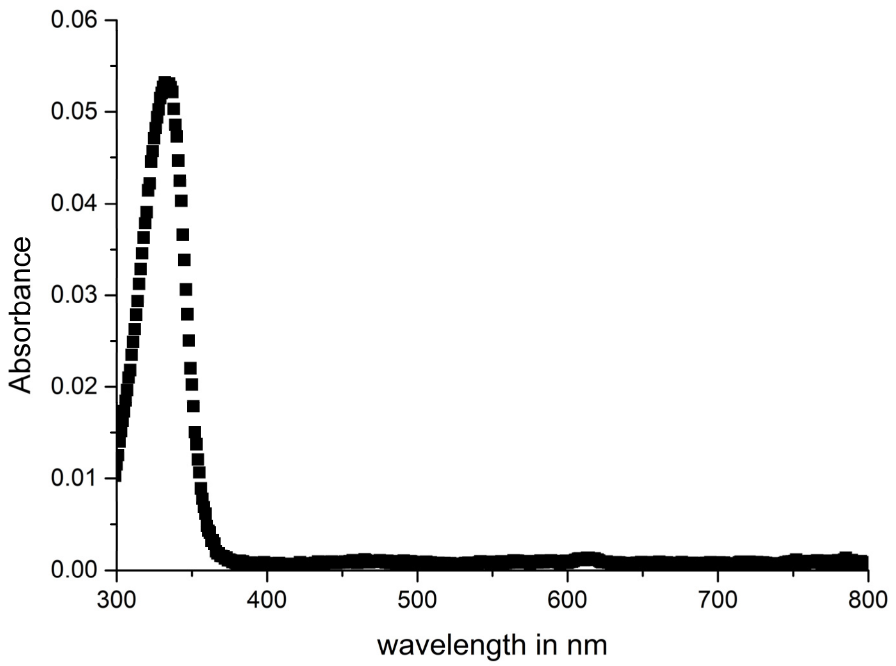

Figure S62. UV/VIS spectrum of **2** (MeCN, d = 1.0 cm, c = 2.1·10^{-6} mol/L).

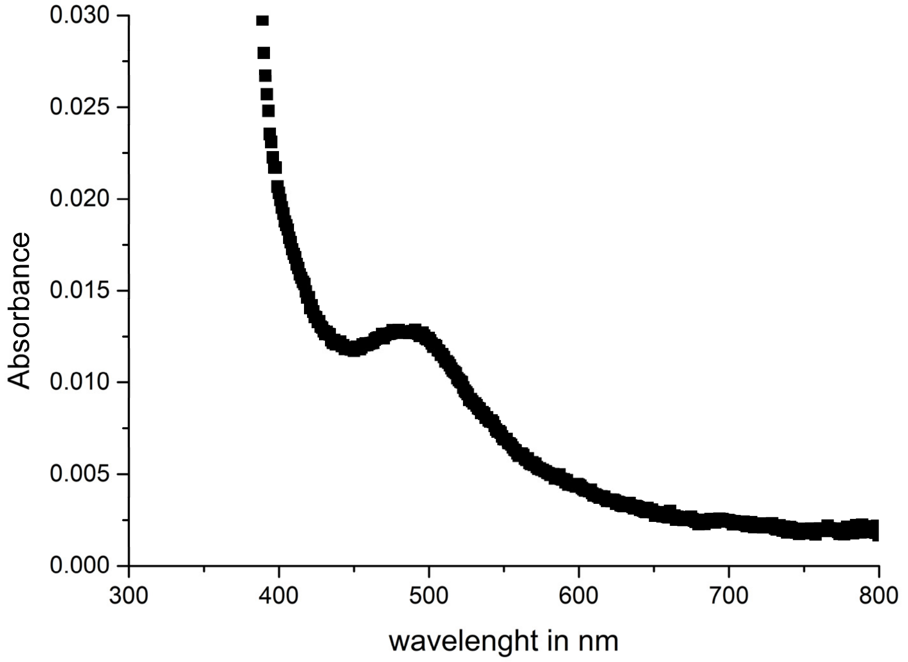

Figure S63. UV/VIS spectrum of **2** (MeCN, d = 1.0 cm, c = 9.0·10^{-4} mol/L).

Spectra of 5

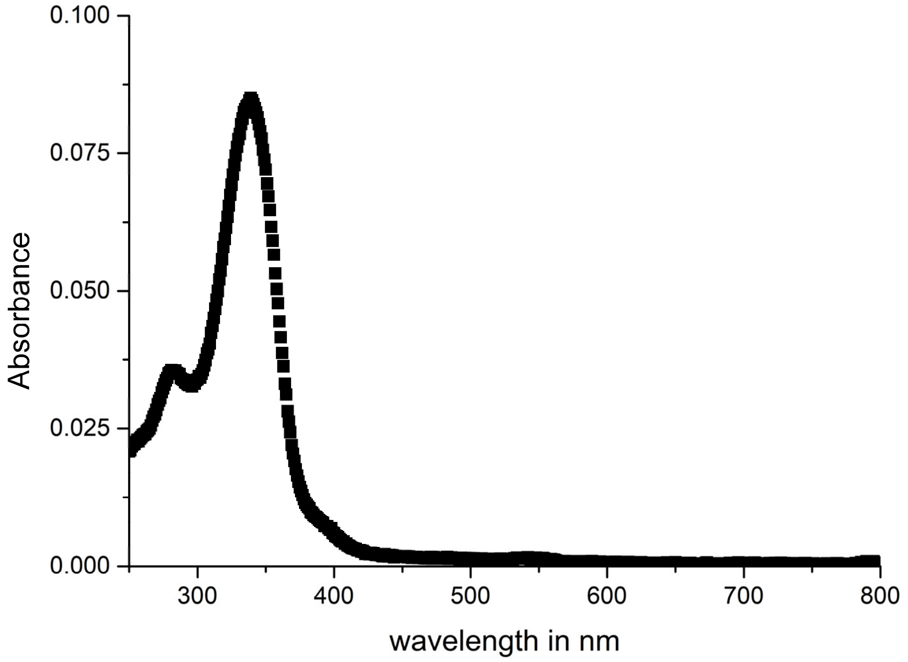

Figure S64. UV/VIS spectrum of **5** (DCM, d = 1.0 cm, c = $1.2 \cdot 10^{-5}$ mol/L).

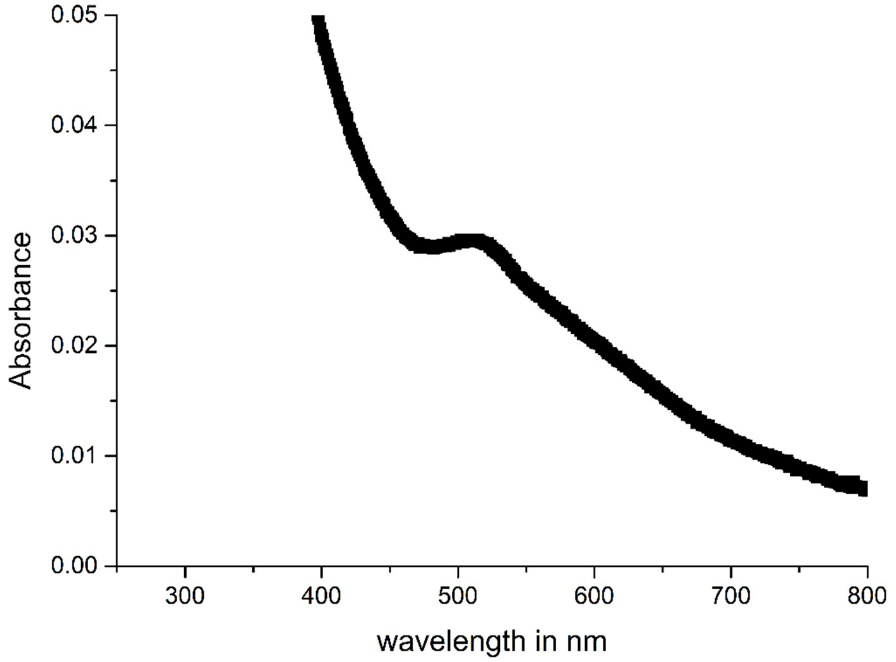

Figure S65. UV/VIS spectrum of **5** (DCM, d = 1.0 cm, c = $6.1 \cdot 10^{-4}$ mol/L).

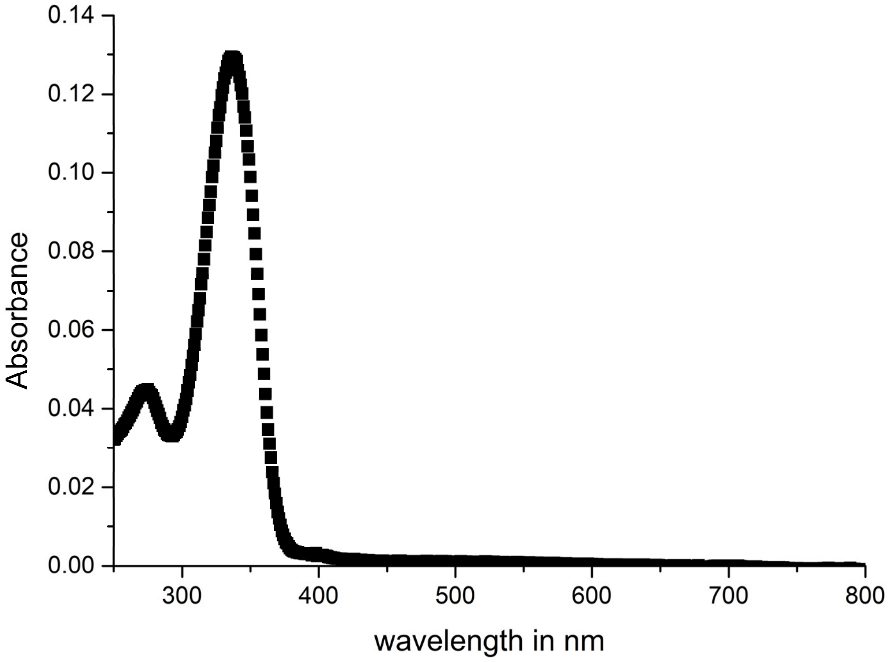

Figure S66. UV/VIS spectrum of **5** (MeCN, d = 1.0 cm, c = 1.3·10^{-3} mol/L).

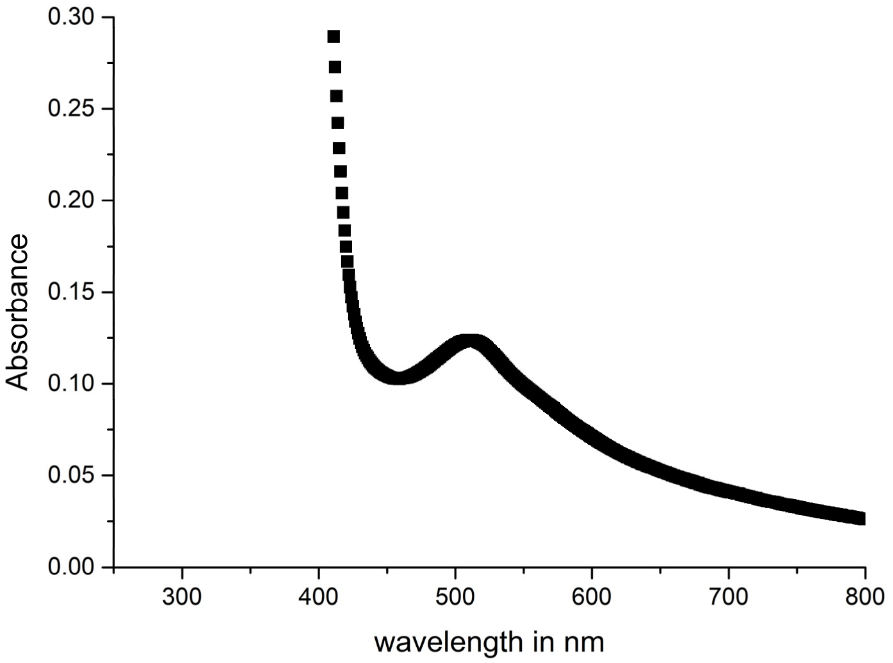

Figure S67. UV/VIS spectrum of **5** (MeCN, d = 1.0 cm, c = 3.7·10^{-6} mol/L).

Spectra of 9

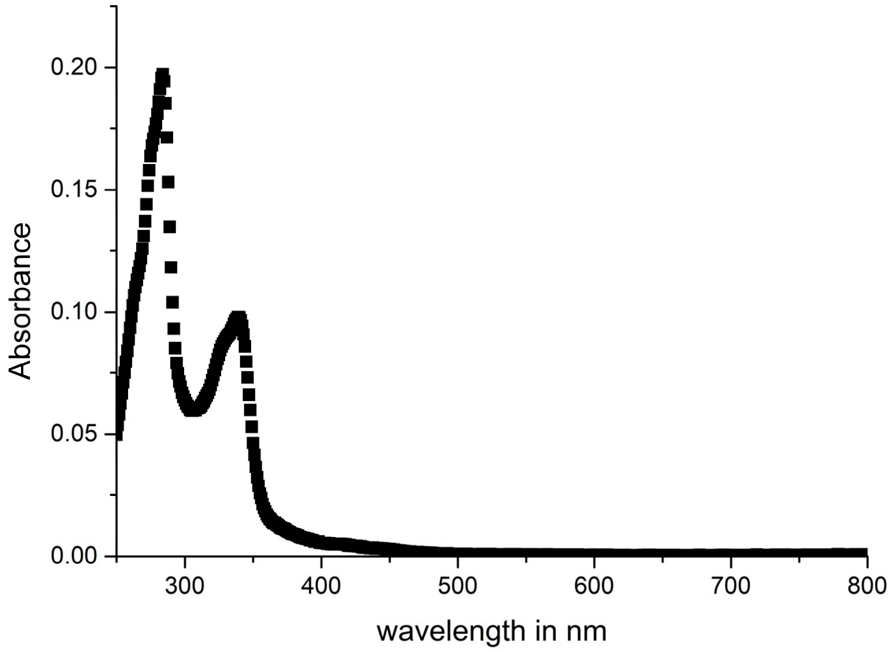

Figure S68. UV/VIS spectrum of **9** (MeCN, d = 1.0 cm, c = $9.0 \cdot 10^{-6}$ mol/L).

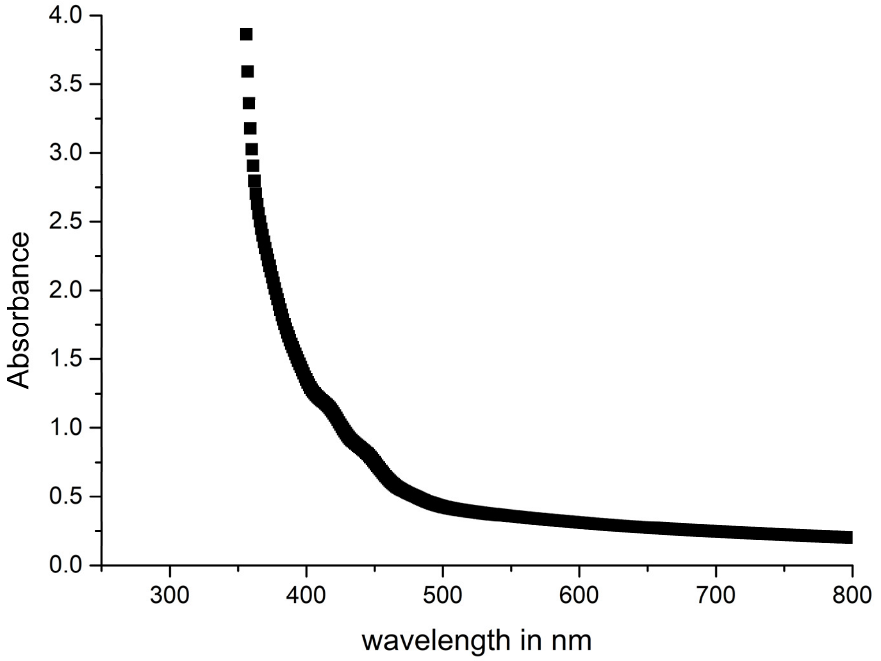

Figure S69. UV/VIS spectrum of **9** (MeCN, d = 1.0 cm, c = $9.7 \cdot 10^{-4}$ mol/L).

Spectrum of 16

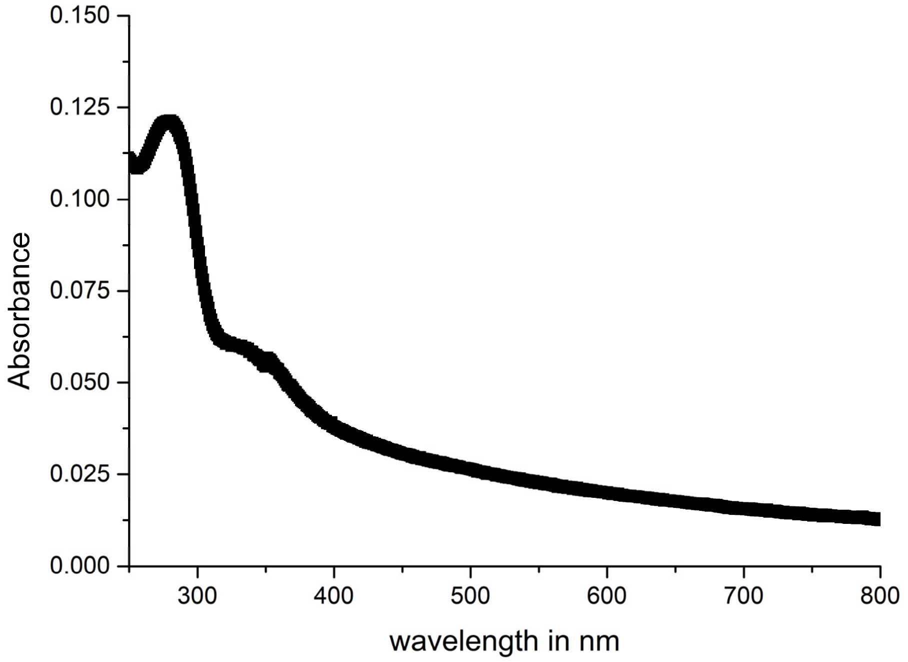

Figure S70. UV/VIS spectrum of **16** (MeCN, d = 1.0 cm, c = $3.5\cdot10^{-6}$ mol/L).

Spectrum of 17

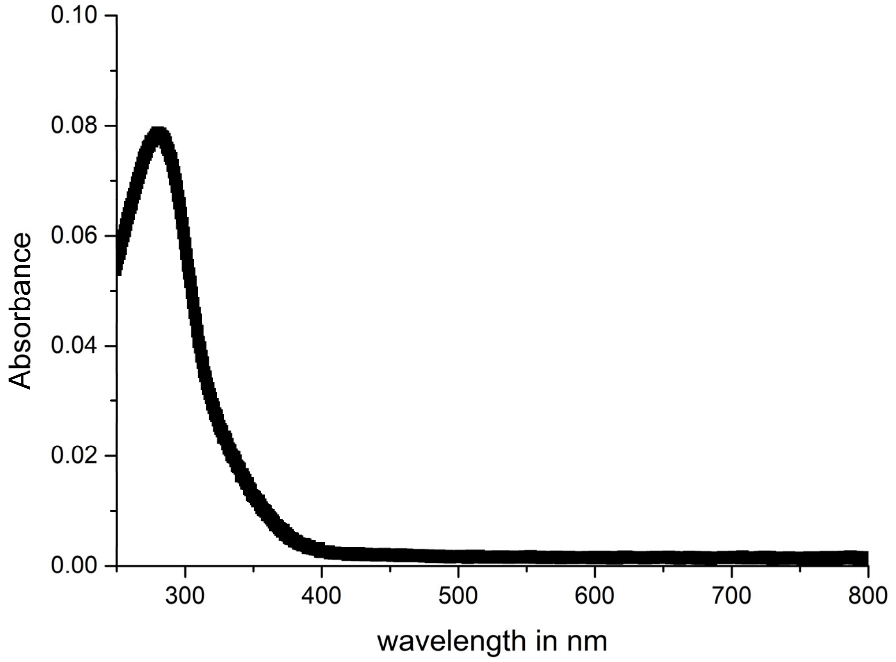

Figure S71. UV/VIS spectrum of **17** (MeCN, d = 1.0 cm, c = $3.6\cdot10^{-6}$ mol/L).

5. Cyclic voltammograms of the reductive potential region

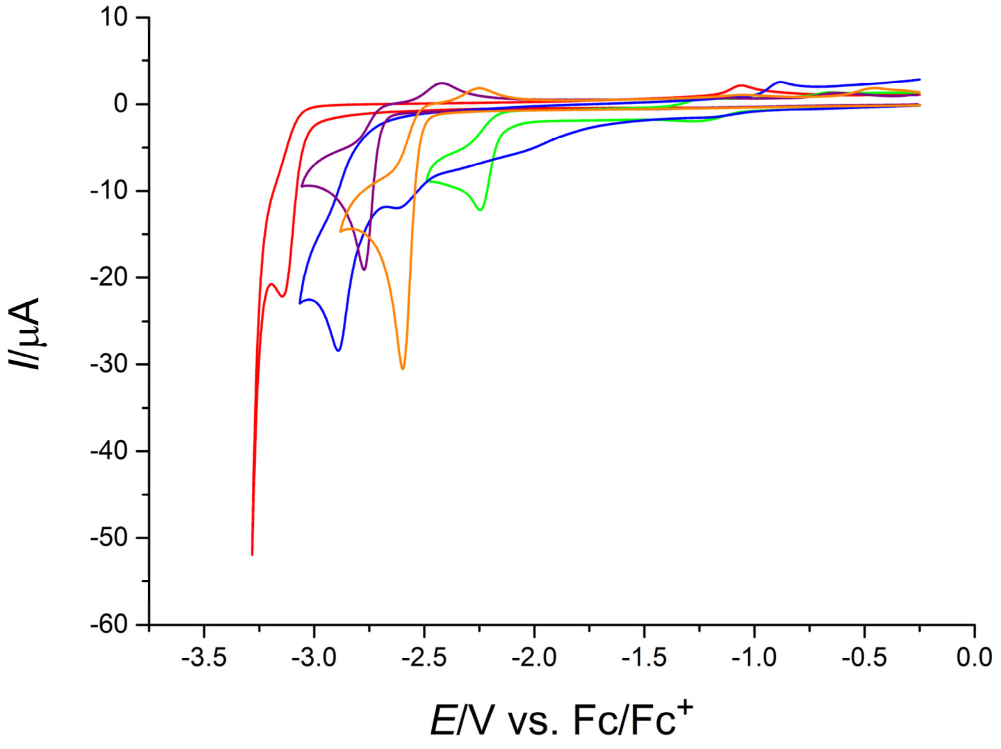

Figure S72. Cyclic voltammograms of **5** (red, 0.25 mM), **9** (green, 0.20 mM), **16** (blue, 0.34 mM), **17** (purple, 0.20 mM) and **19** (orange, 0.48 mM); reductive potential region in MeCN/0.1 M NBu_4PF_6 at a glassy carbon disk electrode; v = 0.1 V s^{-1}; the pre-peak in the cyclic voltammogram of **16** is an artifact of the background current.

6. Additional X-ray structure analyses

In addition to the X-ray structure analyses of new compounds presented and discussed in the manuscript, we provide here the molecular structures of starting materials or side products. The general remarks referring to the X-ray structure analysis from the experimental part of the manuscript apply here as well.

CCDC 2001145 (**1**), 2001141 (**7**) and 2001144 (**20**) contain the supplementary crystallographic data for this paper. These data can be obtained free of charge via www.ccdc.cam.ac.uk/data_request/cif, or by emailing data_request@ccdc.cam.ac.uk.

Molecular structure of 1

We observe four independent molecules of **1** in the asymmetric unit with Cl-Cl distances ranging from 3.038(1) to 3.298(1) Å, which are below the sum of the van der Waals radii (3.50 Å for Cl-Cl interactions[1]) indicating intermolecular halogen interactions. The C1-Cl1-Cl2 angles range from 169.0(1)° to 174.4(1)° and are close to a linear arrangement. The N1-C1-N2 angles range from 109.3° to 109.7° and the C1-Cl1 distances from 1.690(1) to 1.685(1) Å.

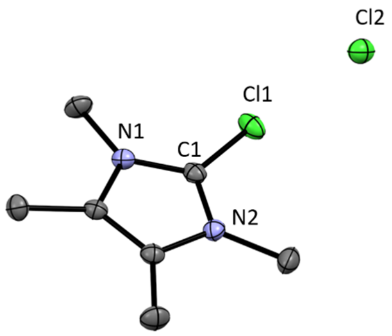

Figure 73. ORTEP plot of the molecular structure of **1** (anisotropic atomic displacement parameters at 50% probability). Hydrogen atoms, two acetonitrile molecules and three molecules of **1** are omitted for clarity. Selected bond lengths [Å] and angles [°]: Cl1-Cl2 3.066(1), C1-Cl1 1.686(1), C1-Cl1-Cl2 171.9(1), N1-C1-N2 109.3(1).

Molecular structure of 7

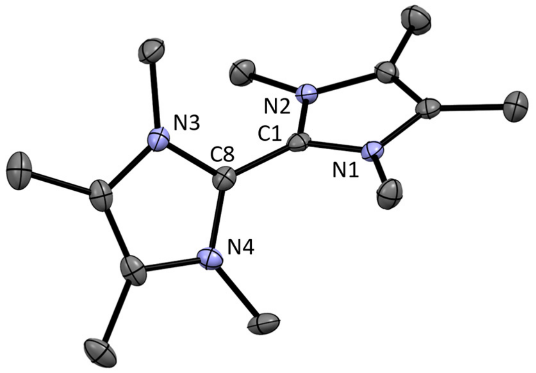

Figure S74. ORTEP plot of the molecular structure of **7** (anisotropic atomic displacement parameters at 50% probability). Hydrogen atoms, the PF_6^- anions and second molecules of **7** are omitted for clarity. Selected bond lengths [Å] and angles [°]: C1-C8 1.448(3), N1-C1-N2 107.8(2), N3-C8-N4 107.9(2). The values of the second molecule are within the standard deviation.

Molecular structure of 20

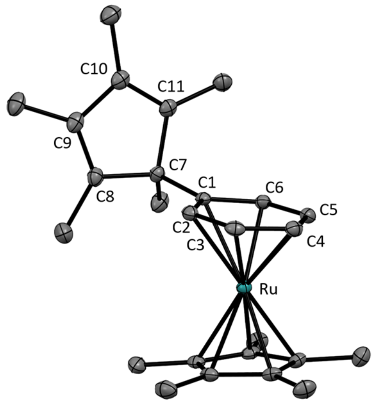

Figure S75. ORTEP plot of the molecular structure of **20** (anisotropic atomic displacement parameters at 50% probability). Hydrogen atoms and the PF_6^- anion are omitted for clarity. Selected bond lengths [Å]: C1-C7 1.534(2), C1-C2 1.421(3), C2-C3 1.417(3), C3-C4 1.409(3), C4-C5 1.413(3), C5-C6 1.418(2), C6-C1 1.423(2), C7-C8 1.524(3), C8-C9 1.344(3), C9-C10 1.473(3), C10-C11 1.346(3), C11-C7 1.536(3), Ru1-C1 2.296(2), Ru1-C2 2.265(2), Ru1-C3 2.223(2), Ru1-C4 2.213(2), Ru1-C5 2.200(2), Ru1-C6 2.209(2).

7. Frontier orbitals of the free ligands

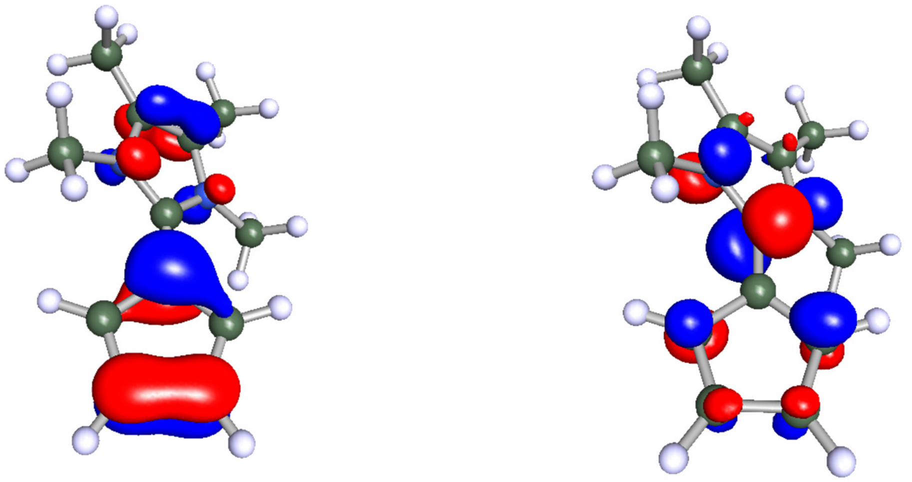

Figure S76. HOMO (left, E = -0.1608 Hartree) and LUMO (right, E = -0.0410 Hartree) of **2**.

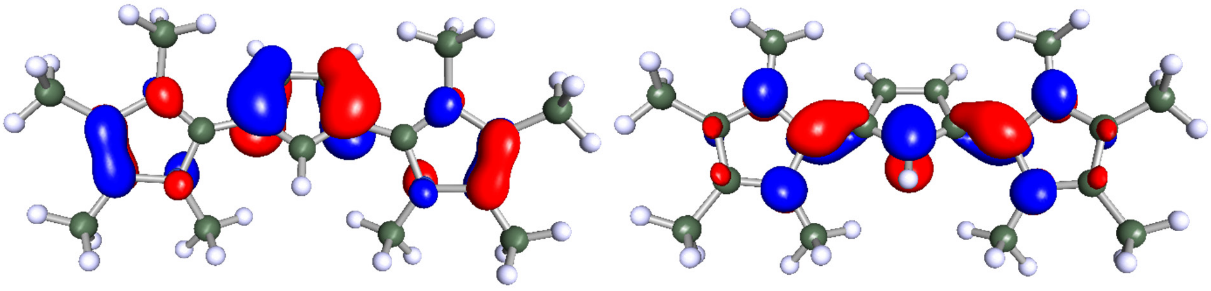

Figure S77. HOMO (left, E = -0.1761 Hartree) and LUMO (right, E = -0.0656 Hartree) of **5**.

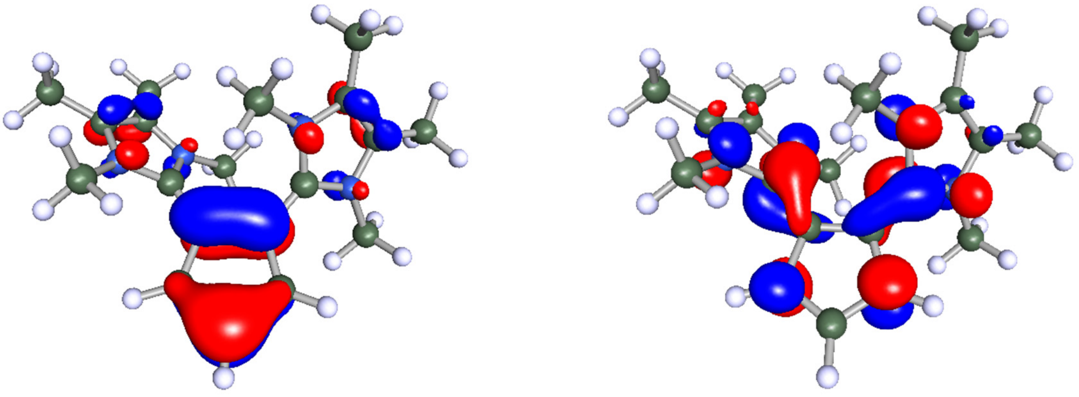

Figure S78. HOMO (left, E = -0.1825 Hartree) and LUMO (right, E = -0.0682 Hartree) of **14**.

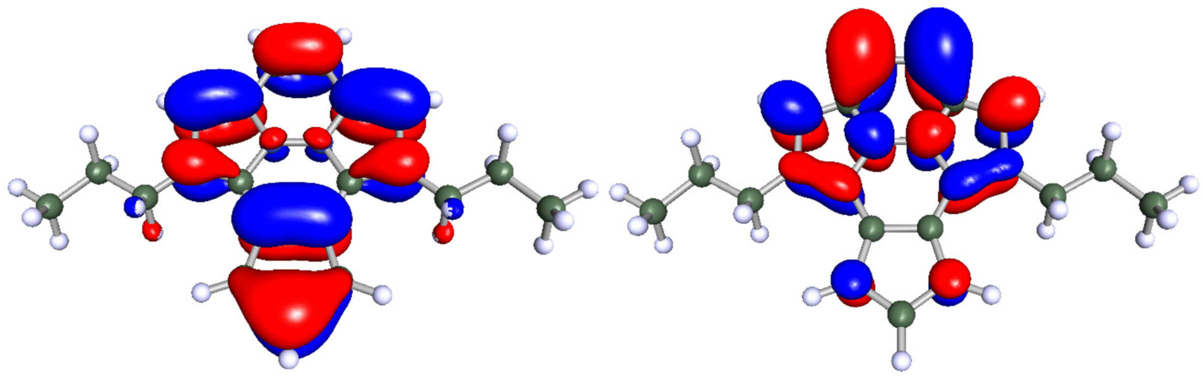

Figure S79. HOMO (left, E = -0.1871 Hartree) and LUMO (right, E = -0.1058 Hartree) of **9**.

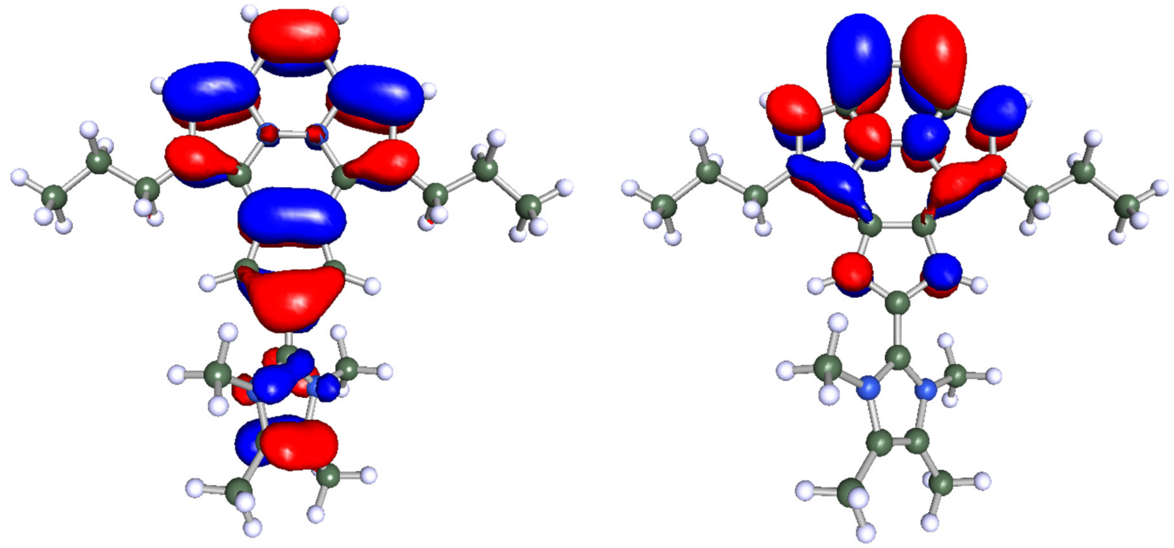

Figure S80. HOMO (left, E = -0.1967 Hartree) and LUMO (right, E = -0.1136 Hartree) of **12**.

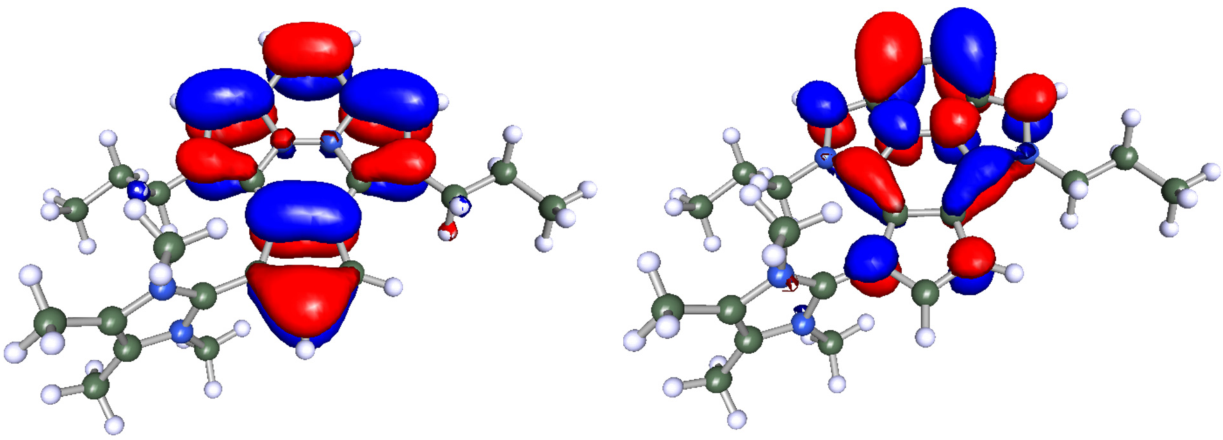

Figure S81. HOMO (left, E = -0.1996 Hartree) and LUMO (right, E = -0.1152 Hartree) of **13**.

8. Reference

[1] R. S. Rowland, R. Taylor, *J. Phys. Chem.* 1996, 7384–7391.
